# Pathways of Membrane Solubilization: A Structural Study of Model Lipid Vesicles Exposed to Classical Detergents

**DOI:** 10.1021/acs.langmuir.2c03207

**Published:** 2023-03-09

**Authors:** Victoria Ariel Bjørnestad, Reidar Lund

**Affiliations:** Department of Chemistry, University of Oslo, Sem Sælandsvei 26, 0371 Oslo, Norway

## Abstract

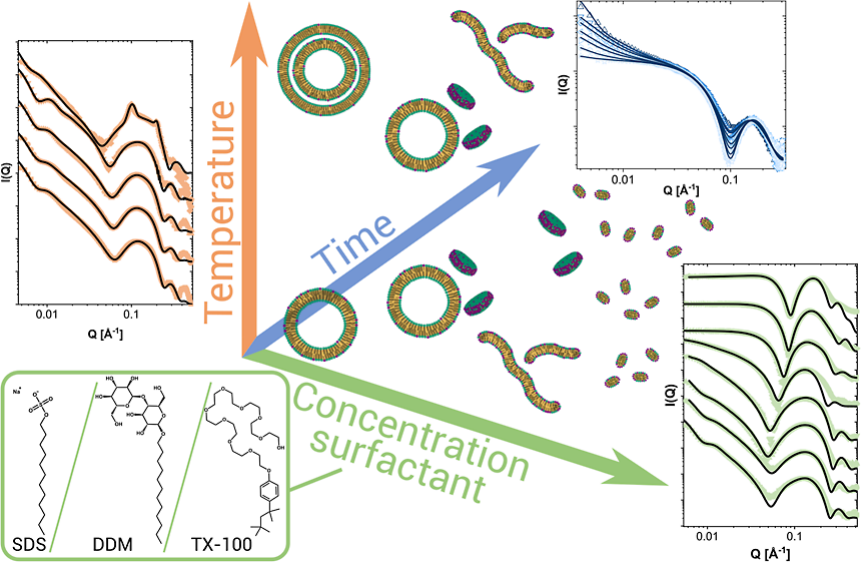

Understanding the pathways of solubilization of lipid membranes is of high importance for their use in biotechnology and industrial applications. Although lipid vesicle solubilization by classical detergents has been widely investigated, there are few systematic structural and kinetic studies where different detergents are compared under varying conditions. This study used small-angle X-ray scattering to determine the structures of lipid/detergent aggregates at different ratios and temperatures and studied the solubilization in time using the stopped-flow technique. Membranes composed of either of two zwitterionic lipids, DMPC or DPPC, and their interactions with three different detergents, sodium dodecyl sulfate (SDS), *n*-dodecyl-beta-maltoside (DDM), and Triton X-100 (TX-100), were tested. The detergent TX-100 can cause the formation of collapsed vesicles with a rippled bilayer structure that is highly resistant to TX-100 insertion at low temperatures, while at higher temperatures, it partitions and leads to the restructuring of vesicles. DDM also causes this restructuring into multilamellar structures at subsolubilizing concentrations. In contrast, partitioning of SDS does not alter the vesicle structure below the saturation limit. Solubilization is more efficient in the gel phase for TX-100 but only if the cohesive energy of the bilayer does not prevent sufficient partitioning of the detergent. DDM and SDS show less temperature dependence compared to TX-100. Kinetic measurements reveal that solubilization of DPPC largely occurs through a slow extraction of lipids, whereas DMPC solubilization is dominated by fast and burst-like solubilization of the vesicles. The final structures obtained seem to preferentially be discoidal micelles where the detergent can distribute in excess along the rim of the disc, although we do observe the formation of worm- and rodlike micelles in the case of solubilization of DDM. Our results are in line with the suggested theory that bilayer rigidity is the main factor influencing which aggregate is formed.

## Introduction

The process of solubilization of a lipid membrane into smaller micellar units is critical in many biotechnological applications ranging from routine cell lysis,^1^ to the purification, isolation, and characterization of membrane proteins^2^ as well as in the process of targeted drug delivery.^3^ Disruption of lipid membranes to yield smaller fragments can be achieved with a range of different substances and methods; however, the most widespread technique is to use detergents/surfactants. The solubilization of lipid bilayers by detergents has been studied by many techniques on a variety of lipids previously. The process was described by a three-step model by Helenius and Simons,^4^ and most authors have interpreted their results in light of this model.^5^ The three-step model divides the solubilization process into three steps depending on the concentration of detergent. In the first step, the surfactant partitions into the bilayer structure. When the concentration of the surfactant is increased, the membrane will eventually become saturated and mixed micelles of surfactant and lipids will start to form, referred to as the onset of solubilization.^6^ As the amount of the surfactant is increased further after saturation, the amount of mixed micelles will increase at the expense of the lipid bilayer structure until all bilayer structures are solubilized into micelles, referred to as the completion of solubilization. Using light scattering, these three critical steps should be seen as (1) an increase in scattered light due to the growth of vesicles as detergent molecules insert and increase the surface area, (2) a decrease in light scattering after saturation as the onset of solubilization progressively forces more lipids into a micellar state which continues until (3) complete solubilization, whereby the scattering will flatten out.^6^ Detergent surfactants, however, constitute a wide variety of different chemical structures, their defining feature simply being that they are composed of one hydrophobic hydrocarbon moiety and one hydrophilic moiety, often referred to as the tail and head of the surfactant, respectively. Different surfactants in themselves form a very diverse set of structures in aqueous solution which also change with on environmental factors such as pH, salinity, and temperature.^7^ One would therefore expect a large variety in the efficiency of partitioning and micellar structures that different surfactants can form in contact with lipid bilayers. These differences have been investigated and discussed previously, particularly in terms of the efficiency of a particular surfactant. Regarding the classical first step of solubilization, the partitioning step, there has been a focus on whether the partitioning occurs solely in the outer leaflet or if the surfactant can flip across the bilayer to partition also in the inner leaflet of a membrane.^5,8^ Literature studies suggest that this could be the main difference between so-called “fast” and “slow” solubilizers,^5^ where the fast solubilizers efficiently can flip across the membrane, thereby saturating the full bilayer and possibly solubilizing the bilayer via open vesicular structures. In contrast, the slow solubilizers will be more limited in their ability to flip across the membrane, thereby having to solubilize the membrane via direct extraction of lipids from the outer bilayer into micelles or a mechanism where pieces are “pinched off” in a fragmentation process.^9^ Triton X-100 and other short ethylene-oxide surfactants are considered fast solubilizers and can equilibrate across both leaflets within milliseconds or seconds after mixing, whereas surfactants such as SDS and DDM are considered slow solubilizers which need minutes or hours to cross the membrane.^10,11^ The differences in the efficiency of transbilayer motion of the surfactants are expected to result in different mechanisms and intermediate structures in the solubilization process.

Factors other than the type of detergent also play a significant role into the efficiency of solubilization, importantly the type of lipid and the temperature. The phospholipids that constitute a biological membrane can vary in their hydrocarbon chain length and saturation as well as in the nature of the headgroup. In addition, other lipid molecules such as sterols can participate in the bilayer structure. The membrane structure and dynamics may also change drastically with temperature as the lipids will transition from a gel state to a liquid crystalline phase as the temperature is increased, with the possibility of different transient phases at intermediate temperatures. It seems intuitive that the solubilization efficiency should increase with temperature, and it has indeed been found that the solubility increases with temperature when the lipids are still in the gel state.^12^ However, it was discovered that for solubilization of saturated lipids with the detergent TX-100, the relationship between temperature and solubility changes direction at the transition temperature,^13^ making lipids in the liquid crystalline phase less readily solubilized with increasing temperature.

Understanding the interactions of detergents with lipid membranes is important for the rational use of these systems in many biomedical, cosmetic, and technical applications.^10^ Despite extensive investigation of the problem, studies of solubilization have mainly utilized methods of turbidimetry,^14−17^ calorimetry,^18^ optical microscopy,^19^ and light scattering.^15,20^ Notably, very few have used methods to directly deduce the structure of metastable intermediates and end products of solubilization, although there are some exceptions where cryo-electron microscopy^21−26^ and small-angle scattering^27,28^ techniques have been used. NMR can provide information on structure and has been complemented to many studies,^15,16,29^ but the broad signals from vesicles and larger aggregates make it difficult to distinguish between different larger structures and possible coexistence between larger and smaller structures. Small-angle X-ray scattering (SAXS) has the potential to reveal information both on perturbations in the vesicle structure on the large scale, the structures of smaller solubilized aggregates, as well as the identification of regions where we have coexistence between structures. The technique is quite sensitive and very non-invasive as the structures can be studied in solution. We therefore considered SAXS to be optimal for investigating the structures involved in solubilization of lipid membranes by detergents.

In this study, we have used SAXS to study the solubilization of DMPC and DPPC lipid vesicles using a selection of three different surfactants: the non-ionic fast solubilizer Triton X-100, the non-ionic slow solubilizer DDM, and the anionic slow solubilizer SDS. The interaction of and solubilization by these different surfactants are expected to vary with the surfactant/lipid ratio as well as temperature, and both these parameters were investigated. The goal of the study was to shed light on the intermediate and final structures involved in solubilization and see if we could further corroborate earlier assumptions. From the SAXS measurement, we could get structural information on the nanometer size domain on the structures still in their native solution. The choice of both DMPC and DPPC as lipids allowed us to investigate the effect of the physical state and lipid mobility on the surfactant interactions more thoroughly.

## Experimental Section

### Sample Preparation

The lipids used (1,2-dimyristoyl-*sn*-glycero-3-phosphocholine powder and 1,2-dipalmitoyl-*sn*-glycero-3-phosphocholine) and the detergent *n*-dodecyl-β-d-maltopyranoside were purchased in the powder form from Avanti Polar Lipids, Inc. Other detergents (Triton X-100, SDS) were purchased from Sigma-Aldrich.

The liposomes were prepared following a protocol alike that recommended by Avanti Polar Lipids. Phospholipid was weighed in and dissolved in a chloroform and methanol mixture (3:1) in a round-bottom flasks. The solvent was evaporated under a flow of nitrogen gas followed by leaving the lipid films under a vacuum to ensure a dry lipid film. The lipid films were hydrated using 50 mM Tris buffer (pH 7.4) in the appropriate volume to form polydisperse multilamellar liposomes. The solutions were sonicated for 20–30 min to reduce the multi-lamellarity of the vesicles. Lastly, the solutions were extruded 21 times through 100 nm filters followed by 21 times though a 50 nm filter at a temperature above the melting point of the lipid to yield unilamellar vesicles of 50 nm in diameter.

The surfactant solutions were prepared by weighing out and dissolving the detergent powder or liquid in the same Tris buffer. The surfactant solutions were mixed into an equal volume of liposome solution at the same temperature as they would be measured later and equilibrated for 4 h. The only exception is the 10 °C measurements that were equilibrated at 5 °C before being measured at 10 °C. For the kinetic measurements, all the samples would be mixed at the same temperature they would be measured at. Density measurements were performed at different temperatures on the different surfactant to accurately determine the molecular volumes for the later fit analysis.

### Small-Angle X-ray Scattering Measurements

The measurements presented in this article result from several different synchrotron beamlines: the BioSAXS beamline BM29^30^ and the Time-Resolved Ultra SAXS (TRUSAXS) beamline ID02^31^ at the European Synchrotron Radiation Facility (ESRF) and the Small- and Wide-Angle X-ray scattering beamline Swing^32^ at Soleil (Paris, France).

All the static, pre-equilibrated data sets were collected at BM29, however, also the slow kinetic data for the TX-100 and DPPC mixtures at 10 °C were collected here. The storage and exposure temperatures were set to one of the temperatures used in the study: 10, 20, 25, 30, or 45 °C before the samples were put into the sample holder. The automated sample changer was set to load 50 μL of the sample for each in-flow measurement and inject into a quartz glass capillary of 1 mm diameter. 10 scattering frames of 0.5 s each were detected for each sample, with an energy of 12.5 keV. The distance from the capillary to the Pilatus3 2M detector was 2.867 m. The background sample (Tris buffer) was measured between each sample measurement, and the capillary was cleaned between sample measurements. Software at BM29 was used for the data scaling and azimuthal integration of the 2D detector images to 1D curves. Water was used as a primary standard to scale the data to absolute intensity. Each individual SAXS frame in each sample and buffer was checked for radiation damage before performing averaging of frames and subtraction of backgrounds in SAXSutilities2 software to give the final SAXS curves presented in this paper.

The data collected from ID02 include the kinetic data from TX-100 mixed with DPPC at 20 and 30 °C and TX-100 mixed with DMPC at 10 °C. The two components were mixed by the stopped-flow device and transferred to the measurement capillary. The measurements presented here were performed at a SAXS detector distance of 2 m and a wavelength of 0.995 Å. The background was measured by injecting a large volume of Tris buffer into the mixing chamber of the stopped-flow device from a third syringe, and the system was cleaned with buffer between measurements. The 2D measurements were normalized and azimuthally averaged to obtain the 1D curves using ID02 software. Averaging of curves, background subtractions, and binning of data were carried out in SAXSutilities2 software.

The data collected from the Swing beamline include all the kinetic data involving DDM and SDS. The two components were mixed by the stopped-flow device and transferred to the measurement capillary. The measurements presented here were performed at a SAXS detector distance of 2.6 m and a wavelength of 1.033 Å. The background was measured by injecting a large volume of Tris buffer into the mixing chamber of the stopped-flow device from a third syringe, and the system was cleaned with buffer between measurements. The 2D measurements were normalized and azimuthally averaged to obtain the 1D curves, and averaging of curves, background subtractions, and binning of data were carried out in Foxtrot software.

### Density Measurements

For the density measurements of the different surfactants, a DMA 5000 density meter from Anton Paar was used, located at the Department of Chemistry, University of Oslo. Water, buffer, and the respective surfactant in the buffer were measured at 10, 20, 25, 30, and 45 °C, and the density and apparent molecular volumes were calculated. The result of the density measurements can be found in the Supporting Information (Section S1, Figure S1, Table S1).

### Data Analysis

QtiSAS software developed and maintained by Dr. Vitaliy Pipich was used to implement the analytical scattering models described in detail in the Supporting Information and to fit the models to the experimental data. BioXTAS RAW software was used to calculate the inverse Fourier transforms (IFTs) via GNOM. A description of the different analytical scattering models can be found in Section S2 of the Supporting Information, and all the resultant fit parameters of the analysis of all the SAXS data can be found in Sections S3 and S4.

## Results and Discussion

### Characterization of Lipid Vesicles and Surfactant Micelles

SAXS measurements at different temperatures were performed on DMPC and DPPC vesicle structures prepared, as described in the Experimental section. The characterization of the structure was performed by fitting an analytical SAXS model to the data. The model used in this article is the simple three-shell model, where the lipid bilayer is modeled as consisting of a hydrocarbon layer surrounded by an inner and outer layer of polar headgroups. A detailed description of the model can be found in the Supporting Information Section S2.1, with an illustration shown in Figure S2. More complex models can certainly be used to attempt to extract further molecular detail from lipid vesicle SAXS patterns,^33^ but since the solutions studied here are expected to yield complex intermediate structures and coexistence of structures, a simpler model is preferred in this case. The bilayer is often modeled to include a separate methyl region of the bilayer which has a lower electron density compared to the methylene regions. We find, however, that the three-shell model gives an equally accurate description of the data in this *Q*-range as compared to a detailed scattering density profile model which includes a lower electron density of the methyl groups in the center of the bilayer. The complexity of the model is necessarily kept low since the model will be further used to analyze vesicles in coexistence with other structures in the surfactant/lipid mixtures. The SAXS measurements for DPPC and DMPC vesicles at different temperatures with the acquired fits of the three-shell model can be found in the Supporting Information (Figure S5). Changing the temperature mainly causes changes in the volume occupied by the lipid tails which increases with the temperature (a decrease in the density of the lipids) and the thickness of the hydrocarbon layer which decreases with rising temperature. Both show a drastic change at the transition temperature where the lipids go from being in the tightly packed, fully extended gel state to the more disordered liquid-crystalline phase. Other parameters remain largely unchanged (see full parameter list in Tables S2 and S3). The model overall describes the data well, although it does deviate more for the measurements of vesicles in the gel state. This could be due to failing to accurately describe possible asymmetry across the bilayer which would cause a shallower minimum at intermediate *Q*-values, and notably this minimum is less shallow in previously characterized 100 nm DMPC liposomes^34^ compared to the 50 nm used in this study. Although the vesicles were extruded to 50 nm, there is still a small fraction of multilamellar vesicles present which varies slightly with the preparation (∼0.25 on average, see Tables S2 and S3). Zwitterionic PC liposomes are also known to somewhat cluster together in solution, which might explain deviations at the lower Q values. The model should allow for any effects of the detergents, such as partitioning into and particularly solubilization of the membrane, to be modeled easily.

The different surfactant micelles were characterized using different variations of a core–shell model.^35^ For the SDS micelles, a simple ellipsoidal micelle model with a hard-sphere structure factor has been used to account for the inter-micellar repulsion (see Figure S6 for fits of neat micelles and fit parameters in Table S4). A triaxial ellipsoid micelle model has been used for the DDM micelles (see Figures S4 and S7 and Table S5). This has been shown to be the most appropriate to describe the scattering from this surfactant apart from direct calculation from molecular modeling.^35^ For the TX-100 micelles, a fuzzy spherical core–shell model that permitted mixing of the tail group into the shell allowed an accurate representation of the data; however, the TX-100 micelles show a complicated structure as described elsewhere,^36^ so the analytical model is only assumed to uniquely describe the micelle scattering for comparison with the mixed solutions and not necessarily to give an accurate description of the pure TX-100 conformation (see Figure S8 and Table S6). A detailed description of all the analytical scattering models can be found in the Supporting Information (Sections S2.2–S2.5). These models have also been applied in several other publications.^35,37,38^ We note that the CMC for SDS is lowered in the Tris solution, with micelles existing down to 0.7 mg/mL (2.4 mM), whereas for Triton X-100 and DDM, they are very close to their common values in water of 0.3 mg/mL (0.48 mM) and 0.08 mg/mL (0.2 mM), respectively (see Section S3.1 for more details). A decrease in CMC for ionic surfactants is expected in solutions of higher ionic strength,^39^ which is in line with our observations here, although we note that for Tris buffers, there has previously been reported a no linear trend, with a decrease at lower Tris concentrations followed by an increase at higher concentrations.^40^ Our concentration of Tris (0.05 M), however, places us well within the lower concentrations; thus, a decrease is still expected.

### Interactions of SDS with DPPC and DMPC Bilayers

**Figure 1:**
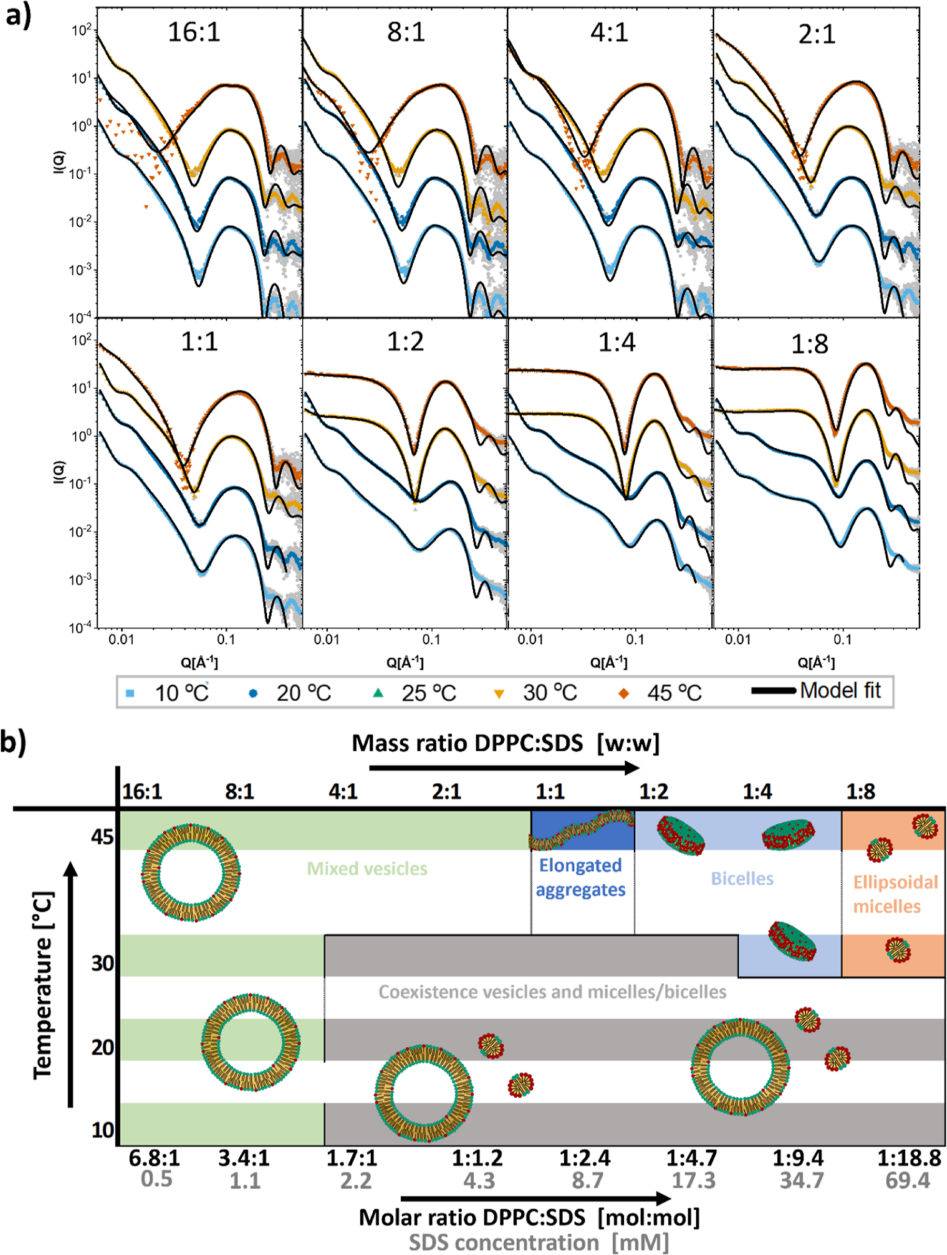
Structures of solubilization of DPPC by SDS. (a) SAXS curves of mixtures of DPPC and SDS at indicated mass ratio DPPC/SDS with model fits. Mass concentration of lipid was always 2.5 mg/mL (molar concentration: 3.4 mM). Mass concentrations in mg/mL of detergent were from lowest to highest: 0.16, 0.31, 0.63, 1.25, 2.5, 5, 10, and 20 (molar concentrations in mM: 0.5, 1.1, 2.2, 4.33, 8.7, 17.3, 34.7, and 69.4). (b) Overview of the resultant structures deduced from the analysis of the SAXS measurements of DPPC mixed with SDS in the indicated mass ratios at different temperatures.

Figure 1a displays the SAXS data for the longer tailed lipid DPPC (16 carbon acyl chains) mixed with the anionic surfactant SDS along with the obtained model fits. Fit parameters can be found in Tables S6–S13. An overview of the morphologies obtained from the analysis is shown in Figure 1b. The first clear observation from the scattering data is that DPPC is very resistant to complete solubilization at the lower temperature. Even at the 1:8 DPPC/SDS mass ratio, corresponding to approximately 20 SDS molecules per lipid molecule, we still see a very significant scattering contribution from lipid vesicles, apparent from the low *Q* data showing the characteristics of vesicle scattering. The results from the fit analysis show that there is partial solubilization of the bilayer at 10 and 20 °C starting at the 4:1 ratio, and the bilayer is then very gradually solubilized more as more surfactant is added. The model that fits best with the data in this case is a gradual solubilization into ellipsoidal micelles. At the higher temperatures however, the onset of solubilization is first at the 2:1 ratio for 30 °C and at the 1:1 ratio for 45 °C. For 30 °C, we then have a gradual solubilization with coexistence between saturated vesicles and bicelles until the 1:2 ratio where a pure bicelle model with SDS accumulated in the rim of the disc fits the data best. Bicelles or disc-like micelles have also been observed for SDS solubilization under low salt concentrations previously.^26^ For the 45 °C, we do not seem to have the same coexistence stage, instead it seems that we have an intermediate stage with elongated aggregates at the 1:1 ratio followed by discs at higher ratios. This observation fits well with the proposed idea of Johansson et al.^41^ that the higher bending rigidity of the DPPC lipids in the gel state promotes direct formation of discs rather than intermediate, thread-like structures: below the melting temperature at 30 °C, we only see gradual transformation from vesicles to bicelles, while the reduced rigidity above the melting temperature at 45 °C allows the formation of elongated aggregates. However, at the very low temperatures, we do not see the formation of discs at all. Presumably, we here have a different mechanism of solubilization all together, with SDS micelles possibly extracting lipids from the bilayer gradually. This is corroborated by the observation that the onset of solubilization in these samples is close to the point where we start to observe micellar structures in the pure SDS samples.

**Figure 2:**
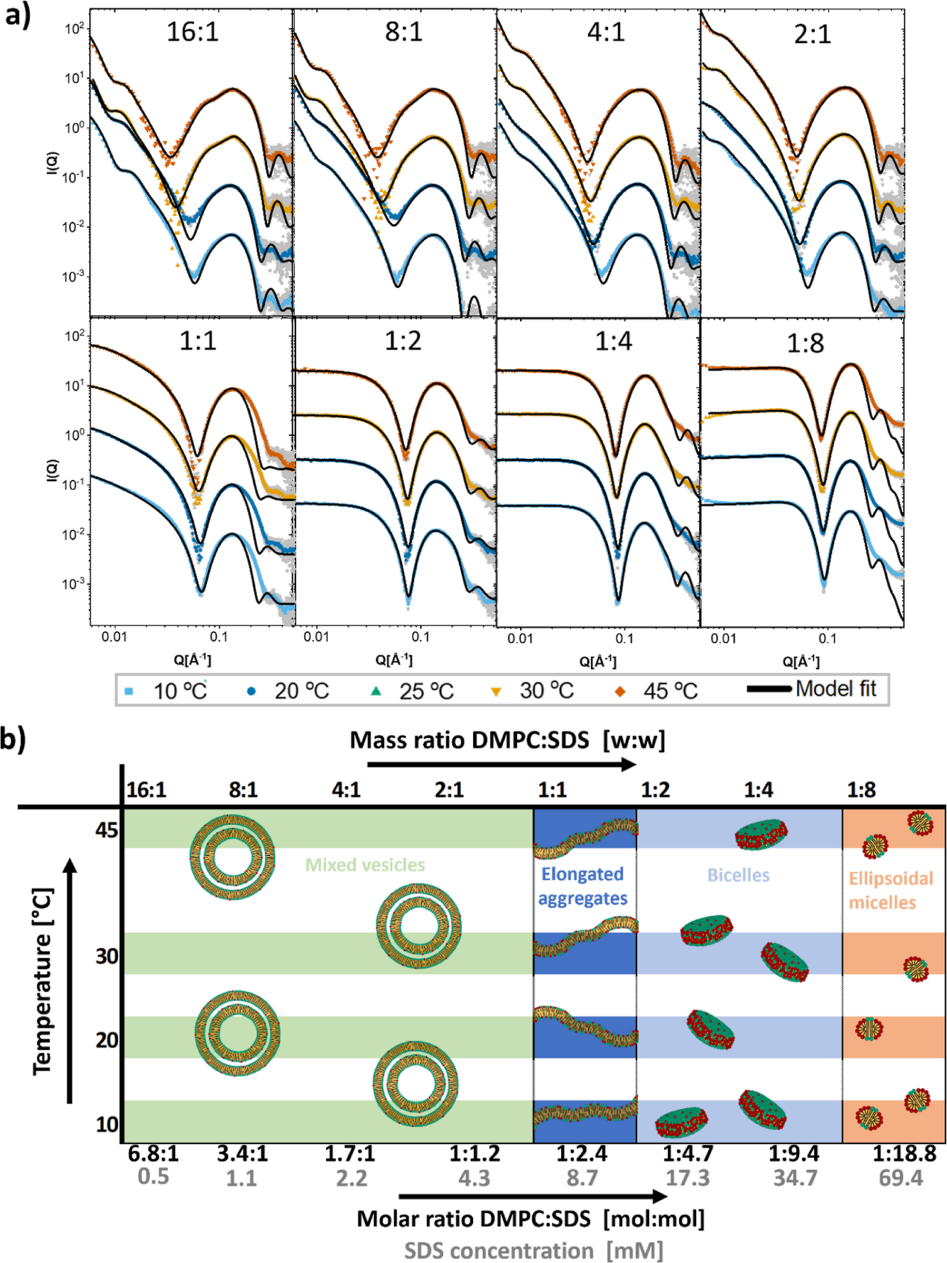
Structures of solubilization of DMPC by SDS. (a) SAXS curves of mixtures of DMPC and SDS at indicated mass ratio of DMPC/SDS with model fits. Mass concentration of lipid was always 2.5 mg/mL (molar concentration: 3.7 mM). Mass concentrations in mg/mL of detergent were from lowest to highest: 0.16, 0.31, 0.63, 1.25, 2.5, 5, 10, and 20 (molar concentrations in mM: 0.5, 1.1, 2.2, 4.33, 8.7, 17.3, 34.7, and 69.4). (b) Overview of the resultant structures deduced from the analysis of the SAXS data of DMPC vesicles mixed with SDS in the indicated mass ratios at different temperatures.

Figure 2a shows the SAXS patterns of different mixes of DMPC bilayers with SDS at different temperatures and mass ratios. Fit parameters from the analysis can be found in Tables S14–S20. An overview of the obtained morphologies at different mass ratios and temperatures is displayed in Figure 2b. From general observations regarding the scattering patterns, the structures seem to be quite independent of the temperature. At the lowest concentrations, there is a change in the position of the low intensity Bragg peaks of the vesicles, where the reasonable assumption would be that SDS increases the lamellar spacing of the bilayers due to repulsion between inserted SDS molecules, and this is also confirmed through the fit analysis. An increase in lamellar spacing is expected if the SDS is present in the adjacent bilayers of the vesicles.^42^ All the samples were equilibrated for 4 h, so we can assume that many of the SDS molecules have had time to flip across the bilayer. Although the equilibration across the leaflet has been previously found to be more than 270 min for higher ratios of SDS/lipid when using egg-PC lipids,^11^ it seems from our results that the SDS has not only equilibrated across the outer bilayer but also across the adjacent multilayers in this time, seen from the increased lamellar spacing. Equilibration across multilayers has been observed at the short time scale for other detergents but not for SDS.^22^ The disappearance of lipid vesicles occurs at the 1:1 ratio for all the different temperatures, corresponding to approximately a 1:5 molar ratio, and after this, we have solubilization of the vesicle structures into smaller micellar units. There is no change in the overall structure of the solubilized aggregates with the temperature; most of the differences can be accounted for by the expected changes in the density of the lipid/surfactant assembly. Analytical modeling shows that small bicelles are the best fit to the data above the 1:1 ratio. These bicelles are very small, with a radius that is approximately the same size as the thickness, but although one might think that a highly oblate ellipsoidal micelle might fit equally well, this type of structure would give a broader minimum at intermediate *Q*. The bicelle structure fit the data until the 1:8 mass ratio, where an oblate ellipsoid model fits the data better. There is, however, also the possibility that bicellar structures would coexist with pure ellipsoidal SDS micelles at these concentrations. At the point of solubilization at the 1:1 ratio, the bicelle model does not fit to the data, and like DPPC at 45 °C, we instead observe a type of elongated structure. Although a rodlike micellar structure fits the data at the low *Q* very well, as can be seen in Figure 2a, it does not manage to fit the scattering at high *Q* values. It is very possible that at this ratio, we have a coexistence of many different structures; this has been observed for many different surfactant-lipid mixtures,^25,27,38,41,43−46^ and so, the same scenario is highly probable for our mixture. Although coexistence between bicelles and vesicles would seem appropriate since these structures fit well with the mixing ratios around 1:1, the scattering pattern is characteristically different than that for this type of coexistence and rather suggests that the coexistence is between disc and rodlike aggregates which can account for the scattering at low *Q*. Such rodlike structures could be simple micellar rod, but elongated sheet or tubular vesicle is also a possibility.^21^

### Interactions of DDM with DPPC and DMPC Bilayers

**Figure 3:**
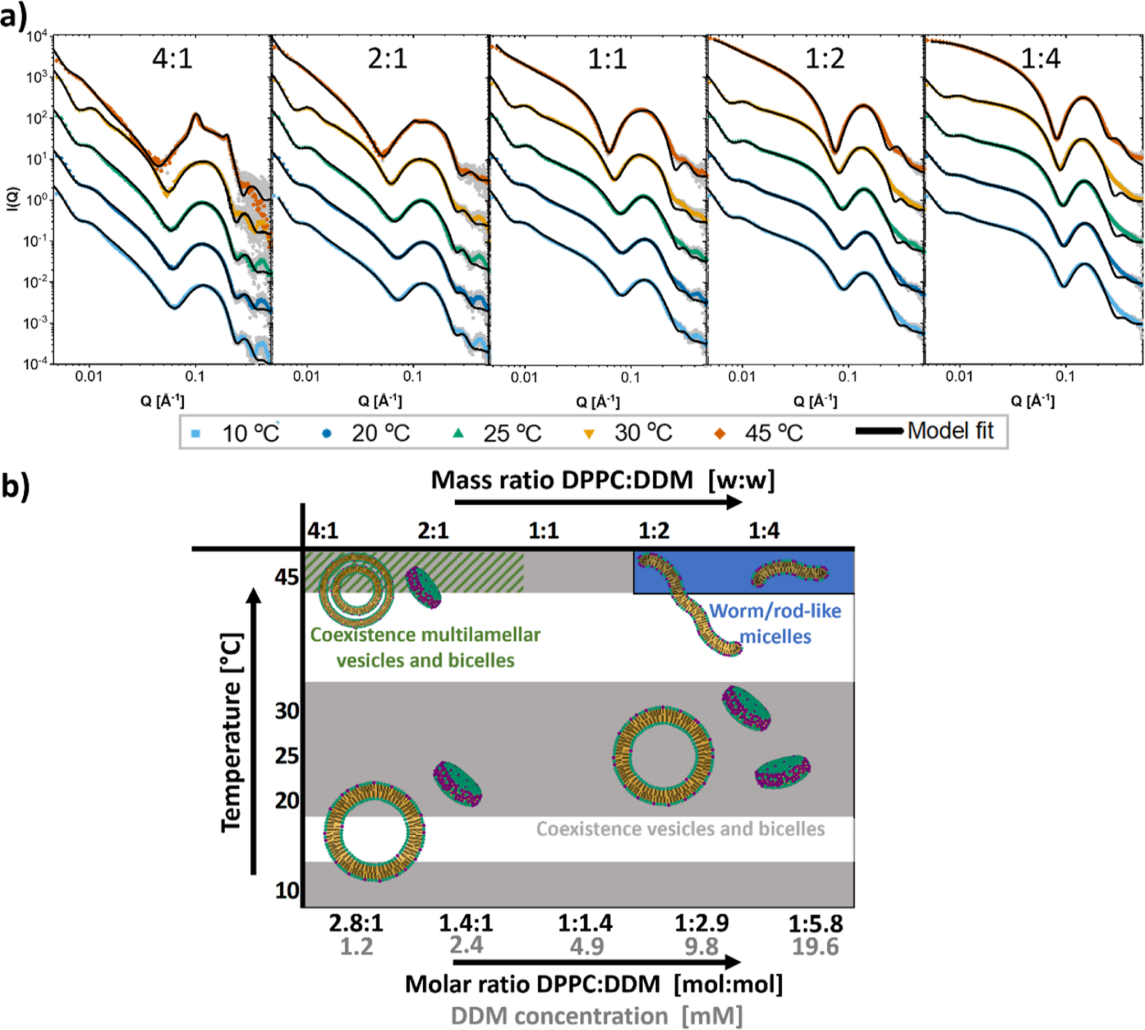
Structures of solubilization of DPPC by DDM. (a) SAXS curves of mixtures of DPPC and DDM at indicated mass ratio of DPPC/DDM with model fits. Mass concentration of lipid was always 2.5 mg/mL (molar concentration: 3.4 mM). Mass concentrations in mg/mL of detergent were from lowest to highest: 0.63, 1.25, 2.5, 5, and 10 (molar concentrations in mM: 0.6, 1.2, 2.4, 4.9, 9.8, and 19.6). (b) Overview of the different structures found at different ratios of DPPC/DDM and different temperatures. (c) Kinetic measurement of DPPC mixed with DDM at 20 °C in a 1:1 mass ratio with model fits (line).

Figure 3a displays the SAXS measurements of DPPC mixed with DDM at different mass ratios and temperatures along with the obtained model fits. Fit parameters can be found in Tables S22–S27. In Figure 3b, an overview of the morphologies deduced from the SAXS analysis is displayed. At temperatures below the melting point of DPPC, we exclusively see partial solubilization of the bilayer, and a coexistence model with a mixture of vesicles and bicelles fits well with the data. Only at the two highest concentrations of DDM at 45 °C, there is complete solubilization into worm or rodlike micelles. At the lowest concentrations of DDM at 45 °C, we also observe a transition to multilamellar vesicles. Growth of lipid vesicles in the presence of DDM has been observed previously, where it was suggested that DDM could act as a fusogenic agent by interacting with the outer bilayer.^47^

For the fits of DPPC with DDM, an asymmetric four-shell model was used to give a better fit to the experimental data, so that the ratio of lipid/surfactant is not equal in the two leaflets (see eqs S22 and S23). Particularly from the fits of the lower ratios, we can deduce that very little of the DDM has managed to penetrate through to the inner leaflets of the bilayer. This, in addition to the high resistance to solubilization and the fact that it is a much more continuous process at the lower temperatures with no abrupt solubilization point, highly suggests that DDM solubilization of DPPC bilayers works mainly through extraction of lipids from vesicles into bicelles, which has been suggested as the main mechanism for “slow” surfactants such as DDM.

**Figure 4:**
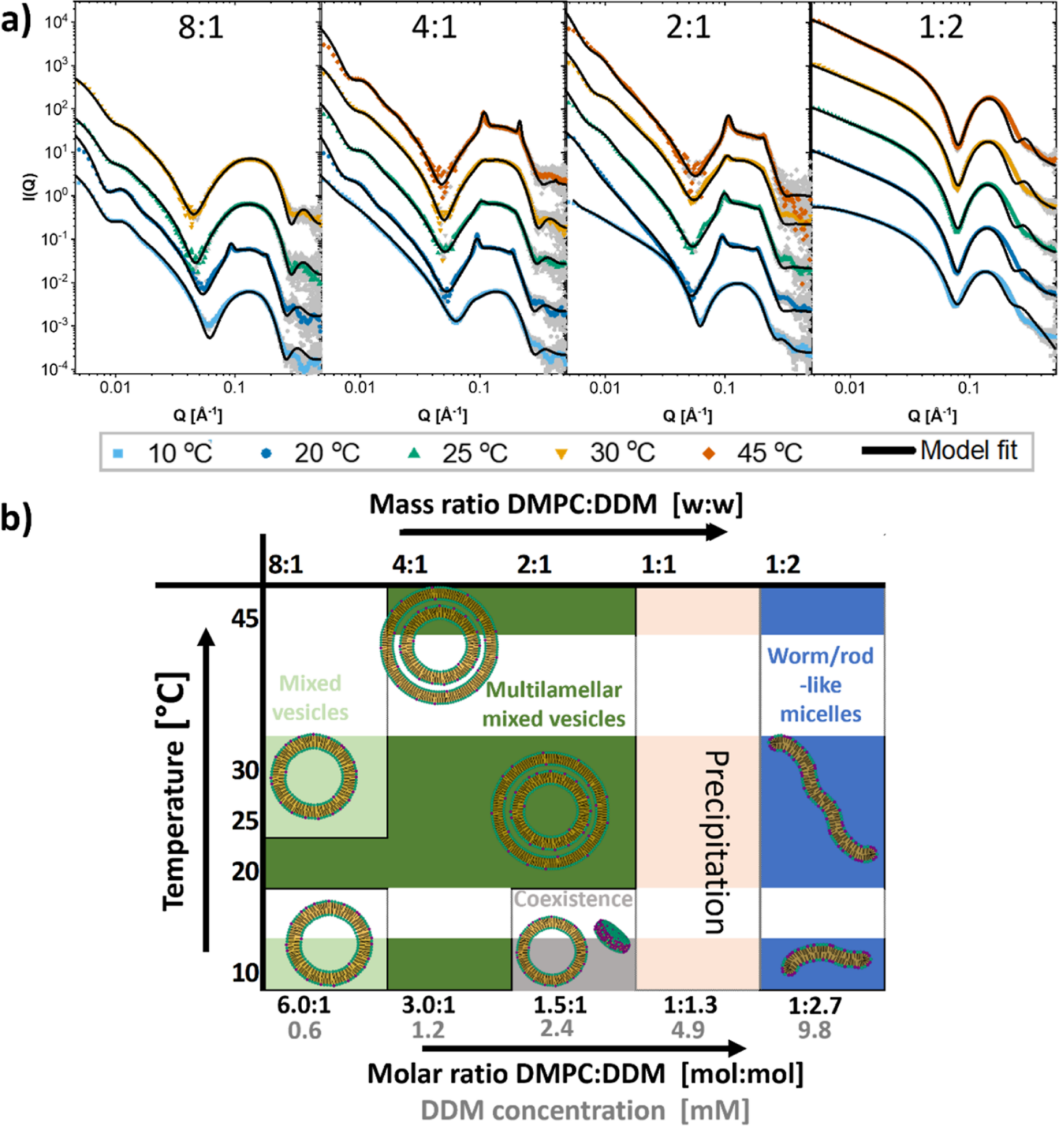
Structures of solubilization of DMPC by DDM. (a) SAXS curves of mixtures of DMPC and DDM at indicated mass ratio of DMPC/DDM with model fits. Mass concentration of lipid was always 2.5 mg/mL (molar concentration: 3.7 mM). Mass concentrations in mg/mL of detergent were from lowest to highest: 0.31, 0.63, 1.25, 2.5, and 5 (molar concentrations in mM: 0.6, 1.2, 2.4, 4.9, and 9.8). (b) Overview of the different structures found at different ratios of DMPC/DDM and different temperatures.

Figure 4a displays the SAXS data from different mixtures of DDM with the shorter chained lipid DMPC at different temperatures along with the obtained model fits. Fit parameters can be found in Tables S28–S31. Figure 4b shows an overview of the morphologies deduced from the SAXS analysis. DDM induces multi-lamellarity also in DMPC vesicles, although at a wider range of temperatures than it does in DPPC, again suggesting that the state of the lipid phase plays an important role in this phenomenon. The DMPC vesicles reach saturation at a lower concentration at 10 °C compared to higher temperatures. At the 1:1 ratio, we have precipitation in all the solutions except at 10 °C (see Supporting Information Figure S10), suggesting that at this ratio, the vesicles must rapidly grow to become unstable in solution. At the highest ratio, we have formation of worm-like micelles at all temperatures except 10 °C, where the aggregates are smaller and better described as shorter, rodlike micelles.

### Interactions of Triton X-100 with DPPC and DMPC Bilayers

**Figure 5:**
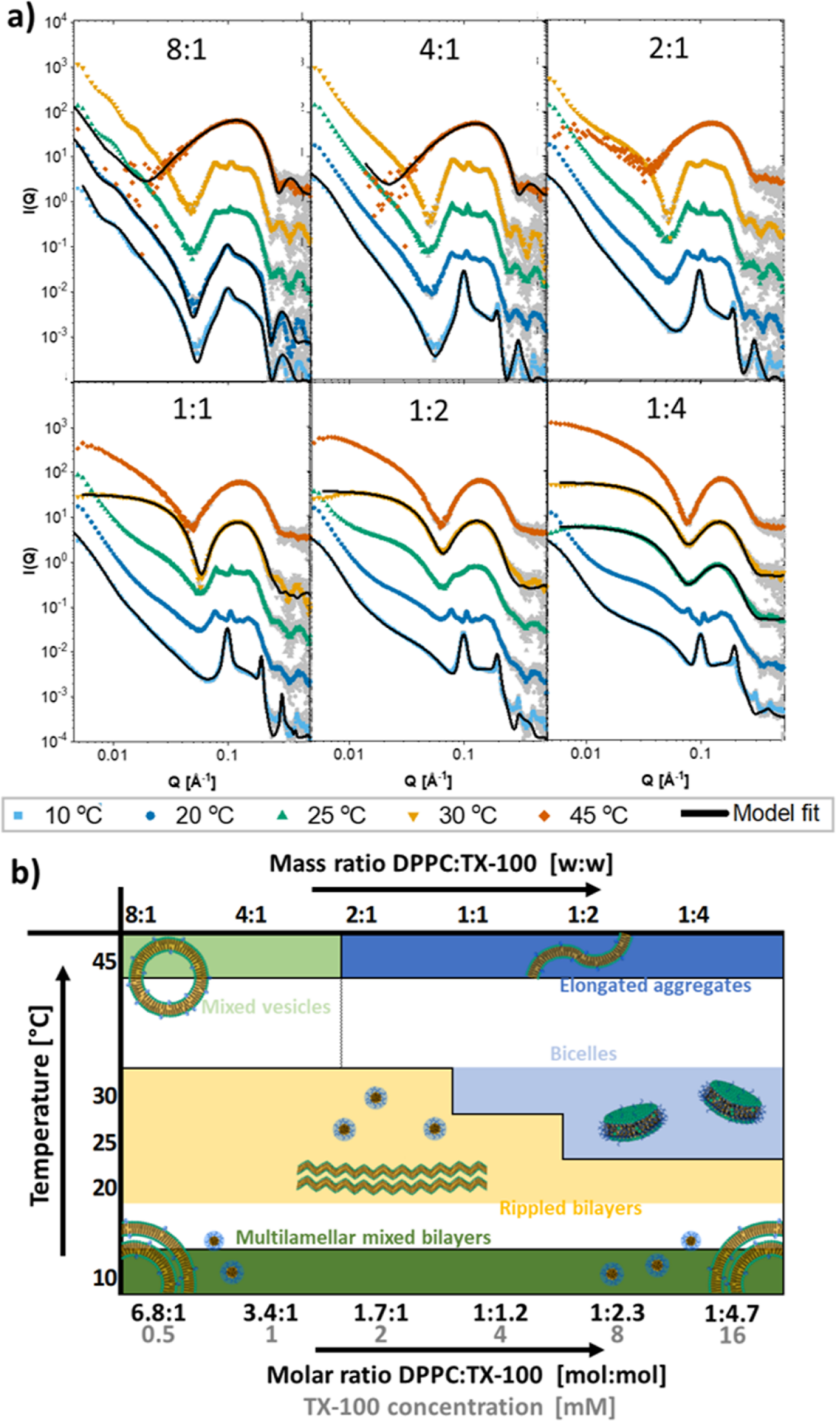
Structures of solubilization of DPPC by TX-100. (a) SAXS curves of mixtures of DPPC and TX-100 at indicated mass ratio DPPC:TX-100 with model fits. Mass concentration of lipid was always 2.5 mg/mL (molar concentration: 3.4 mM). Mass concentrations in mg/mL of detergent were from lowest to highest: 0.31, 0.63, 1.25, 2.5, 5, and 10 (molar concentrations in mM: 0.5, 1.0, 2.0, 4.0, 8.0, and 16.0). (b) Overview of the different structures found at different ratios of DPPC:TX-100 and different temperatures.

When TX-100 is mixed with vesicles of DPPC lipids at different lipid/detergent ratios and temperatures, a wide range of different structures are observed by SAXS. The SAXS data and an overview of the results from the analysis are shown in Figure 5a,b. Fit parameters can be found in Tables S32–S36. Notably, model fits were not possible for all structures, as further described below. At low temperature, below 10 °C, we have formation of multilamellar structures for all the different ratios of DPPC:TX-100, as indicated by strong Bragg peaks in the bilayer scattering at higher *Q*-values. These measurements can be quite well described by a coexistence model where we have multilamellar lipid vesicles, and the TX-100 is distributed either into the lipid phase or existing as pure TX-100 micelles. From the model fits, we can see that the amount of TX-100 that is inserted into the bilayer does increase with the increasing concentration of TX-100, as plotted in Figure S12, but there does not seem to be any solubilization of the lipid vesicles, even at the highest concentration where the molar ratio of TX-100:lipid in the vesicle is approaching 1:1. Whether the structures at 10 °C are actually spherical vesicle structures for all these ratios, however, is not entirely clear from the SAXS data since the characteristic form factor oscillation is not present above the 8:1 ratio. In a separate study on these unique structures, we found by cryo-TEM that the structures at 10 °C are in fact stacks of flat bilayers, potentially formed from collapsing vesicles.^48^ Regardless, TX-100 must cause bilayers to adhere to one another in a stronger manner forming the multilamellar structures. A collapse of the vesicle structure could then be expected since TX-100 is expected to flip into the inner leaflet of the vesicle within milliseconds to seconds,^10^ and so any attractive force caused by TX-100 would be present also on the inside of the vesicle. Moreover, addition of TX-100 and a momentary imbalance of the detergent concentration may in certain intervals lead to osmotic pressure that promotes a deformation.

As we increase the temperature to 20 °C, moving into the yellow marked area of Figure 5b we see that while the scattering in the low *Q*-region largely remains the same, the pattern of Bragg peaks in the bilayer scattering changes drastically for the ratios exceeding 8:1. This pattern of peaks is very characteristic for TX-100 mixed at these temperatures, and the structures have proven to be much too complex to be described by any simple analytical SAXS model. We previously found^48^ that the peak pattern overlaps well with what has been characterized for the metastable ripple phase of DPPC.^49^ We have further investigated this structure using cryo-TEM in a separate study^48^ where we found the structures to be collapsed and rippled bilayers, yielding nanostructures that resemble ridged potato chips. The formation of the ripples is also extremely slow, having time scales equivalent to that of the mixtures at 10 °C to reach the multilamellar end-state, suggesting that the formation follows a similar mechanism, although the end-structure of the bilayer itself is different. The findings in this other study support the theory that a collapse of the vesicles also occur at 10 °C, forming flat and multilamellar structures^48^ but not ripples. Solubilization of DPPC vesicles at 20 °C requires very high amounts of TX-100, as will be shown later in the presentation of the kinetic data where the solubilization process can be observed at a mass ratio of 1:16.

When increasing the temperature to 25 °C, the same structures as at 20 °C persist at ratios below 1:2, but at this ratio, we do start to see the bilayers solubilizing into smaller structures. At 30 °C, solubilization is apparent for even lower ratios. At the 1:1 ratio at 30 °C, we can model the data with a bicelle (discoidal) structure where an excess of the TX-100 distributes around the rim of the discs, but coexistence with spherical TX-100 micelles is required for the higher ratios. This suggest that there is in fact a strong resistance to incorporate the lipids into more spherical micellar structures with more TX-100 since the system appears to remain segregated.

At 45 °C, above the melting point of DPPC (41 °C), the solubilized structures become more elongated, something which is apparent from the gradient at low *Q*-values and can be seen clearly from the pair distribution function resulting from an IFT of the SAXS curves (Figure S11). The scattering pattern does not fit with simple elongated discs or rods, neither a worm-like micelle pattern. Although one would think that the main cause of this transition would be due to DPPC transitioning to the liquid-crystalline state, pure TX-100 micelles also undergo a transition between 37 and 45 °C to more elongated structures^50^ (Figure S8a), so the structure is likely a consequence of the change in the behavior of both TX-100 and the lipids. Cryo-TEM images of the structures formed at a 1:2 ratio of DPPC:TX-100 at 37 °C that was previously collected show a very polydisperse collection of broken leaflets, so it seems likely that the samples at 45 °C consist of similar types of structures (Figure S15).

**Figure 6:**
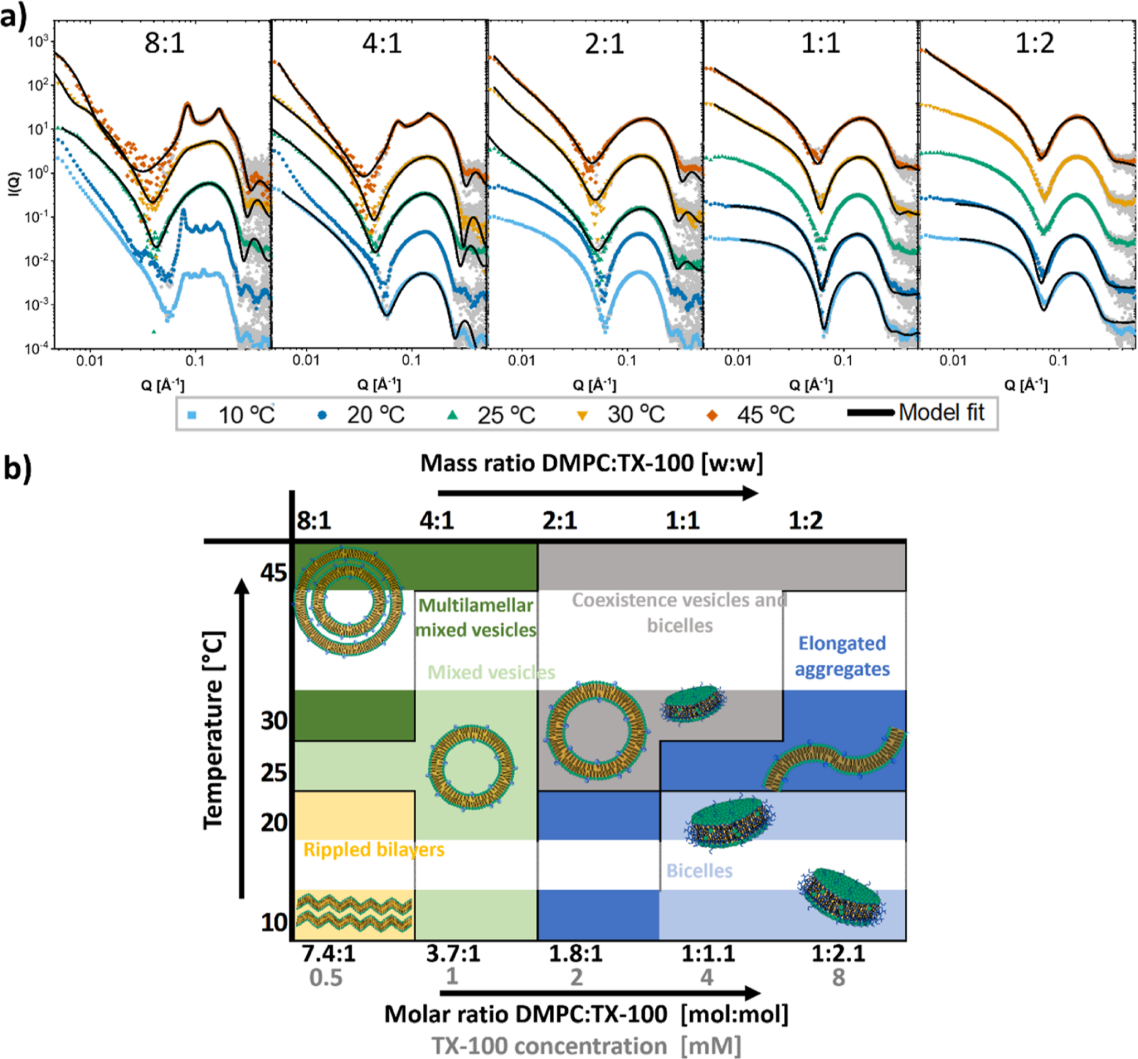
Structures of solubilization of DMPC by TX-100. (a) SAXS curves of mixtures of DMPC and TX-100 at indicated mass ratio of DMPC:TX-100 with model fits. Mass concentration of lipid was always 2.5 mg/mL (molar concentration: 3.7 mM). Mass concentrations in mg/mL of detergent were from lowest to highest: 0.31, 0.63, 1.25, 2.5, and 5 (molar concentrations in mM: 0.5, 1.0, 2.0, 4.0, and 8.0). (b) Overview of the different structures found at different ratios of DMPC:TX-100 and different temperatures.

Going to the shorter chained lipid, DMPC, we not only see some of the same effects as for DPPC but also many striking differences. Figure 6a,b displays the SAXS curves and an overview of the results from the analysis of the different mixtures as mixed and measured at different temperatures. Fit parameters can be found in Tables S37–S41. Similar to the DPPC bilayers, the characteristic Bragg peaks in the bilayer scattering appear at low ratios, for example, at 8:1 DMPC:TX-100 at both 10 and 20 °C. The fact the rippled phase also exists for DMPC below the melting point supports the notion that the physical state (packing) of the lipids plays an important role in the stability of the rippled phase. The concentration range where we see the rippled phase though is much narrower in DMPC compared to DPPC, occurring only the 8:1 ratio in our samples. As the temperature is increased, the ripple phase is no longer observed. A multilamellar vesicle model with symmetric insertion of TX-100 fits to the 8:1 data above 20 °C with increasing degree of multi-lamellarity as the temperature increases. The ripple phase also disappears again as we increase the concentration of TX-100 to the 4:1 ratio although the data are still modeled best with the vesicle model even at 10 °C. First at the 2:1 ratio, do we start to see solubilization, with solubilization occurring more efficiently at the lower temperatures which is inferred both from the model fit and from the IFT analysis giving the pair distance distribution (Figure S13). For the three higher temperatures, we have partial solubilization and an analytical scattering model with coexistence between vesicles and bicelles in solution fitting the data well. For the lower temperatures of TX-100 however, the vesicles seem to be solubilized into elongated aggregates that are characteristically different from vesicles as inferred from the IFT analysis (Figure S13), and a simple bicelle model cannot accurately fit the data at the low *q*-values. At the 1:1 ratio, we have the same situation with coexistence for the two higher temperatures but complete solubilization of the bilayer into small bicellar structures at 10 and 20 °C. The data at 25 °C could not be fitted with a bicelle or coexistence model, and the pair distance distribution function for these structures revealed that the structures are similar to the bicelles found at 10 and 20 °C albeit more elongated in nature. The same holds for the 1:2 ratio at both 25 and 30 °C. We note that at the specific mixtures of 8:1 at 45 °C and 2:1 at 25 °C, the system precipitated.

### Kinetics of Solubilization

This section will present a select set of results that was obtained by time-resolved SAXS measurements for the different detergent/lipid mixtures and temperatures above described in the sections above. For DDM and SDS, kinetic data were only obtained at 20 °C, but for TX-100, some results at 10 and 30 °C are also presented.

**Figure 7:**
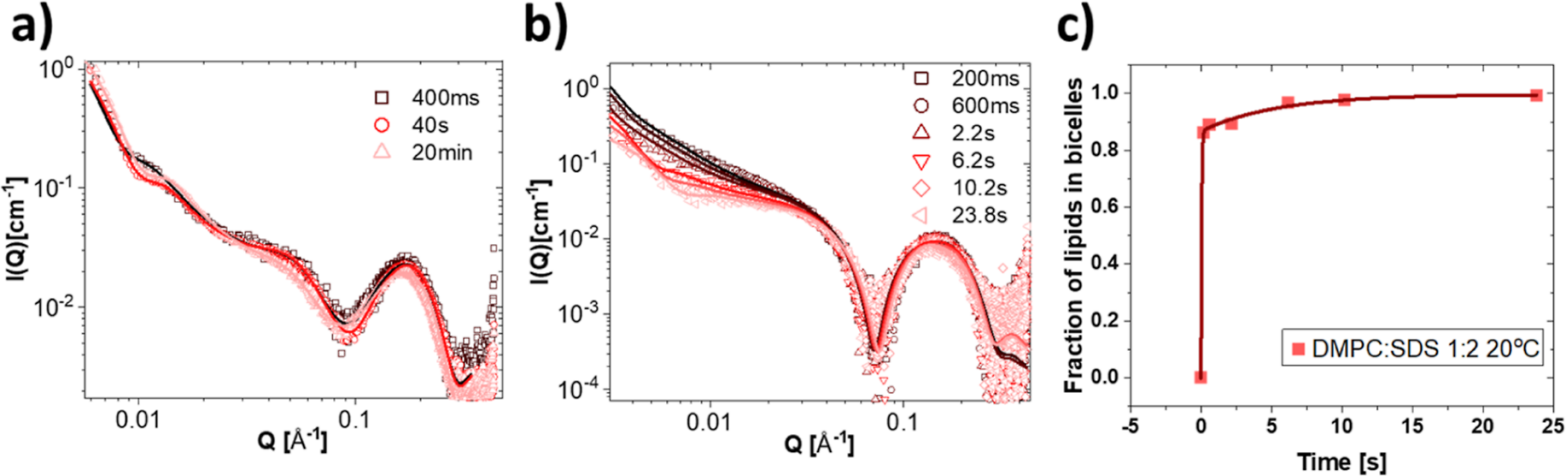
Kinetics of SDS solubilization of DPPC and DMPC vesicles. (a) Time-resolved SAXS measurement of DPPC mixed with SDS at 20 °C in a 1:8 mass ratio with best fits from a mixed vesicle/ellipsoidal micelle coexistence model. (b) Kinetic SAXS measurement of DMPC mixed with SDS at 20 °C in a 1:2 mass ratio with best fits from a mixed vesicle/bicelle coexistence model. (b) Plot of the fraction of lipids in solubilized structures (bicelles) at the different timepoints as found from the model fits in (b).

Figure 7 presents the time-resolved measurements collected at 20 °C for the mixtures of SDS with DPPC in (a) and with DMPC in (b). The obtained fit parameters from the analysis can be found in Tables S42 and S43. The kinetics of the partial solubilization of DPPC at 20 °C is extremely slow. As seen from Figure 7a, even at the 1:8 ratio, we see no change in the scattering pattern for the first 10 min except a small change in the spherical form factor oscillation at the high *Q* values. After 20 min, we can see also a small decrease in the micellar scattering present at the intermediate *Q* values. The pattern is still distinctly different from what is obtained after 4 h though; fit analysis reveals that only about 34% of the lipids have been solubilized at this time, compared to 63% in the equilibrated samples. For a 1:2 mixture of DMPC/SDS, displayed in Figure 7b, we note that the higher radiation damage apparent in SDS solution made acquisition at longer time scales necessary, and only the final steps of the solubilization, where most of the vesicles have already disappeared from solution, could be captured. Interestingly, although the bicelle model along with a small fraction of intact vesicles fits well with the final frames after 10 s, a coexistence between bicelles and vesicles did not fit equally well to the earlier frames; as can be seen from the fits in Figure 7b, the fit is particularly failing to describe the intermediate *Q*-region. This points to the possibility of intermediate elongated structures being present and the scattering thereby actually resulting from the coexistence of at least three different structures. This hypothesis is corroborated by the existence of an intermediate elongated structure also at the 1:1 lipid/detergent ratio in the equilibrated solutions (Figure 2).

**Figure 8:**
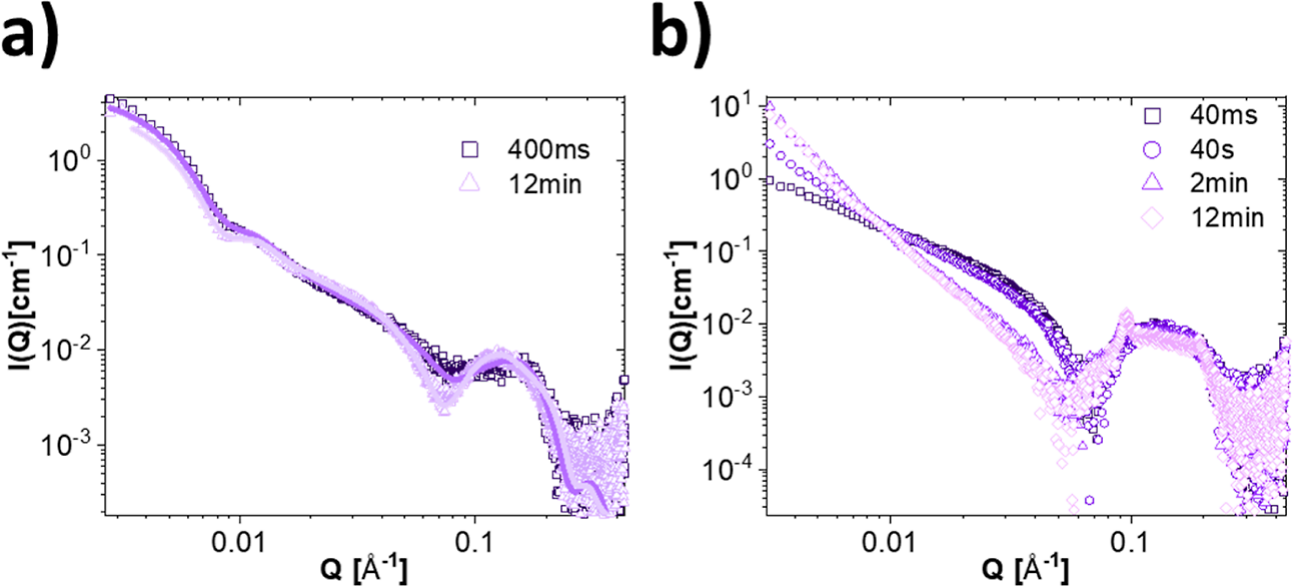
Kinetics of DDM interactions of DPPC and DMPC vesicles. (a) Kinetic measurement of DMPC mixed with DDM at 20 °C in a 1:1 mass ratio. (b) Kinetic measurement of DPPC mixed with DDM at 20 °C in a 1:1 mass ratio with model fits (line).

The kinetics of the partial solubilization of the DPPC bilayers by DDM is on a similar slow time scale to that of the SDS/DPPC mixtures. As displayed in Figure 8a, there is a very slow change from a scattering pattern that is very close to the average of the individual scattering of DPPC and DDM at 400 ms after mixing to one that is closer to the state after 2 h only after 12 min. A model where we have coexistence between pure DDM micelles and DPPC vesicles fits well to the data at 400 ms while coexistence between bicelles and vesicles as used for the static data fit the data at 12 min, further supporting this observation. The obtained fit parameters from this analysis can be found in Table S44. For mixtures of DDM with the shorter chained DMPC lipid at sub-solubilizing ratios where we see increased multi-lamellarity of the vesicles, however, the situation is quite different. The kinetic data presented in Figure 8b reveal peculiar changes in the scattering: the vesicles seem to first solubilize into large, sheet-like structures, as indicated by the *Q*^–2^ slope of the scattering in the low *Q* region for the measurement at 40 s, and then later reform into multilamellar aggregates. The initial solubilization into larger aggregates is very rapid and occurs within the first 40 ms. The formation of the multilamellar aggregates, however, is more elusive: in Figure 4c, the multilamellar structure appears after the first minute after mixing, whereas in repeat measurements, the multilamellar structure appeared as early as 1 s after measurement or did not appear at all before the solution started showing signs of precipitation. It could be that the kinetics is very sensitive to the exact ratio of DDM and lipids and that the stopped-flow instrument failed to reproduce the exact kinetics every time, or that the formation of multilamellar aggregates is dependent on an initialization that is a highly random process. These kinetic measurements suggest a process where the vesicles open into more flexible aggregates and only later reform into multilamellar structures. The opening then occurs very fast, almost being complete already at 40 ms, and the induced multi-lamellarity also appears only after 40 s. Interestingly, we can still see that the oscillation at low *Q*, likely arising from the scattering of the shell, is smeared out, supporting the idea that the bilayers reseal into vesicle structures after opening. Together, this seems like strong indications that DDM-induced multi-lamellarity of DMPC bilayers is caused by a rupture and resealing process rather than direct fusion between vesicles. Concerning the vesicle–worm transition which occurs at higher concentrations of DDM with DMPC at 20 °C, the kinetics of this process is very fast, similar to the opening at the lower concentration, and completes within the first 110 ms (see Supporting Information Figure S14). This shows that the process requires less reorganizational steps and can proceed by a type of fragmentation mechanism where seeds can grow into elongated structures by rapid addition of amphiphiles.

**Figure 9:**
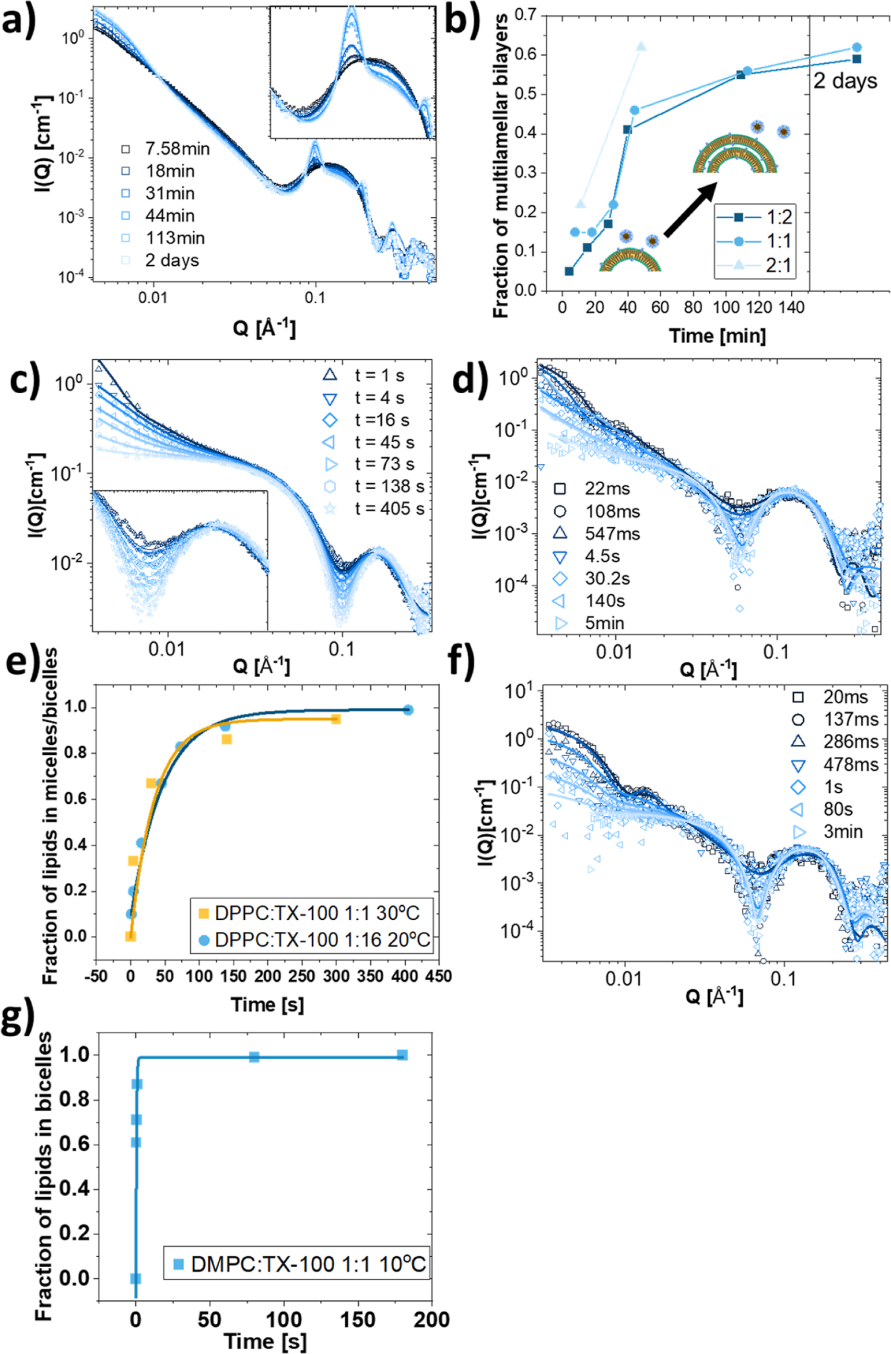
Kinetics of TX-100 solubilization of DPPC and DMPC vesicles. (a) Mixture of DPPC:TX-100 at the mass ratio 1:1 at 10 °C measured at different timepoints from the time of mixing modeled with a gradual increase in the degree of multi-lamellarity. (b) Plot of fraction of multilamellar bilayers as fitted to SAXS curves at different timepoints for three different DPPC:TX-100 w/w ratios mixed at 10 °C. (c) Time-resolved SAXS measurement of DPPC mixed with TX-100 at 20 °C in a 1:16 mass ratio and (d) 30 °C in a 1:1 ratio. (e) Plot of the fraction of lipids in solubilized structures (mixed micelle/bicelle) at the different timepoints as found from the model fits in (c,d). (f) Kinetic measurement of DMPC mixed with TX-100 at 10 °C in a 1:1 mass ratio with model fits (line). (g) Fraction of lipids in solubilized structures (bicelles) at the different timepoints as found from the model fits in (f).

Similar to the effect of DDM on the DMPC bilayer, TX-100 also seemed to induce multi-lamellarity at all ratios at 10 °C. The kinetics of this process is, however, vastly different from what was observed for DDM/DMPC. Modeling the kinetic data with a gradual increase in the fraction of multilamellar vesicles and number of adjacent bilayers describes the process well, as seen in Figure 9a (fit parameters in Table S45, for other ratios in S46 and S47); a conceptual illustration of this model can be seen in the graph in Figure 9b. The kinetic SAXS measurements show that the multi-lamellarity increases very slowly over time: a 1:1 mixture required more than 2 h after mixing to reach a final state at our concentrations (Figure 9a,b). This would suggest that interactions between vesicle structures are also important: an initial collapse could be followed by subsequent adhesion of several collapsed vesicles that would enhance the intensity of the Bragg peaks. The fraction of multi-lamellarity as a function of time for the different ratios are quite similar, as shown in Figure 9b, but does not fit to any simple models, such as single or stretched exponential. This could be because the multilamellar model does not accurately represent the process of collapse and adhesions which needs to be explored further. The kinetics of the formation of the rippled nano-flakes seen at 20 °C with TX-100:DPPC mixtures is likely governed by a similar process as at 10 °C, and this has been discussed further in a separate paper.^48^ There we concluded that the pathway was an implosion of the vesicle followed by ripples induced by the few inserted TX-100 molecules, which stabilizes the structure from solubilization. At high enough detergent concentration, however, solubilization will take place also at the low temperatures. Figure 9c depicts the kinetic measurements of the solubilization of DPPC with a 1:16 mass ratio of lipid:TX-100 along with model fits (fit parameters in Table S48). The process is quite slow at this temperature, taking around than 7 min to complete. As perhaps expected with a ratio of almost 19 TX-100 molecules to 1 lipid molecule, the resultant product is very similar to the pure TX-100 micelles. The kinetics can be described quite well by a simple two-stage model where lipids are being slowly extracted into TX-100 micelles, without any intermediate structures. This slow disappearance of lipids would point toward a mechanism where the micelles extract lipids gradually from the bilayer since we otherwise would expect a more abrupt change in the scattering pattern after saturation of the bilayer. The kinetics of DPPC solubilization by lower amounts of TX-100 at higher temperatures is similar to what we observed at low temperatures and high ratios. For kinetic measurements of the 1:1 ratio at 30 °C, a model where we first have a slow insertion of the TX-100 into the vesicles structure over ∼0.5 s followed by a solubilization into bicellar structures over the next 3 min fits the data quite well (Figure 9d); fit parameters are listed in Tables S49 and S50. Even though the solubilization here occurs at a much lower surfactant ratio than that at 20 °C and results in bicelles rather than spherical micelles, we see that interestingly, the time scale of the process is the same at both temperatures (Figure 9e).

The kinetics of TX-100 solubilization of DMPC are generally too fast to be resolved by SAXS, finishing within the first 30 ms, except for at 10 °C where we can see kinetics at the scale of 1 s (Figure 9f). A model with partial insertion of the TX-molecules from the micelles fits best with the first hundred milliseconds, while a coexistence between bicelles and vesicles fits to the data from 286 ms up to 1 s where we have complete solubilization into bicelles (fit parameters are listed in Tables S51 and S52). It therefore seems that we first see a slow insertion of TX-100 into the vesicles in the first 207 ms followed by a quite abrupt and rapid solubilization into discs that completes within the first second for the 1:1 ratio (Figure 9g).

### Slow versus Fast Solubilizers

**Figure 10:**
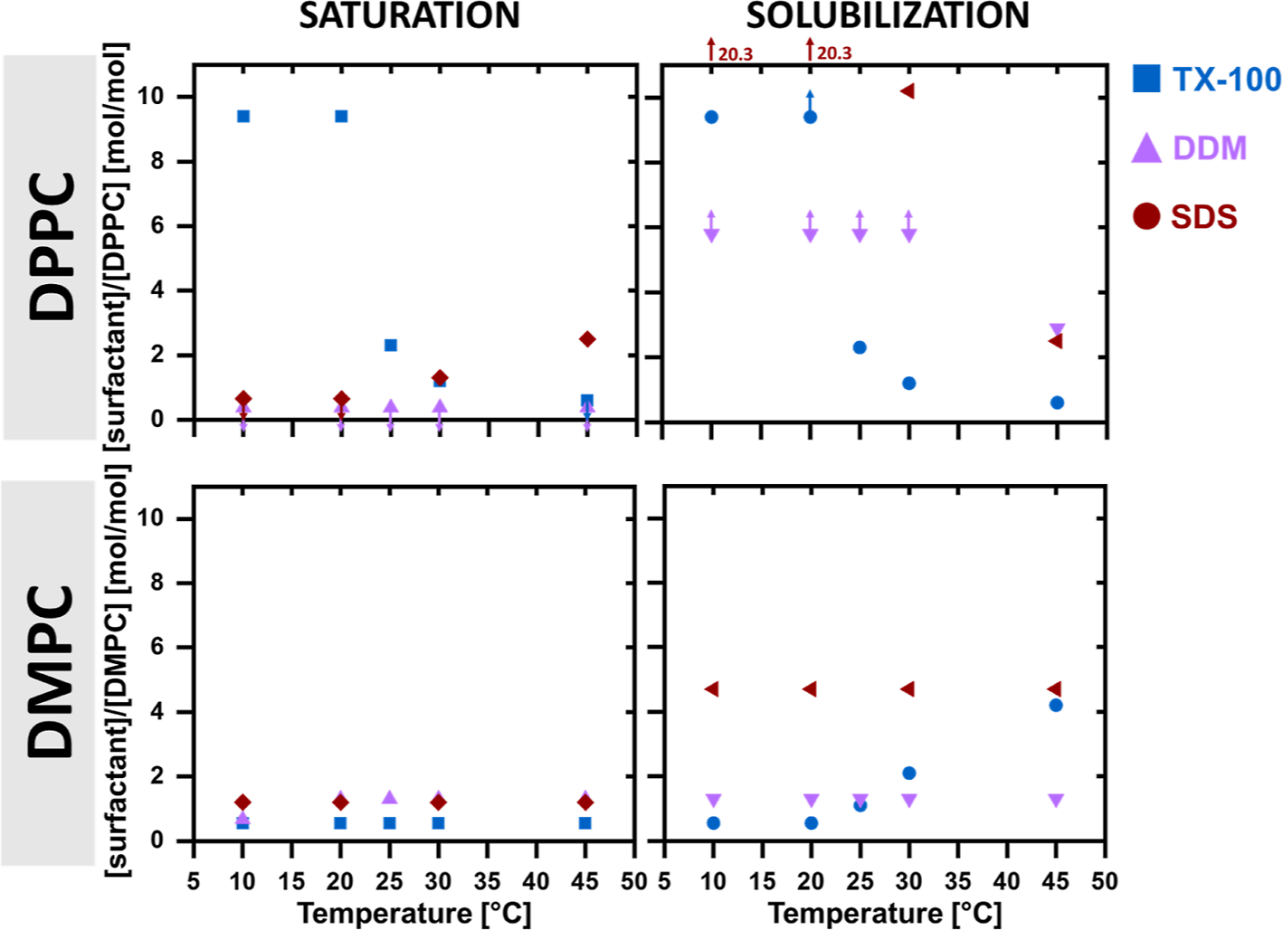
Saturation and solubilization limits deduced from SAXS. The apparent saturation and solubilization limits deduced from the structures of the aggregates found for the three different surfactants in terms of the surfactant molar concentration divided by the lipid molar concentration plotted at a function of temperature for the two different lipids. The arrow mark points where the limit must be either higher (up arrow) or lower (down arrow).

TX-100 is considered a fast solubilizer, whereas DDM and SDS are both traditionally considered slow solubilizers.^5,51^ We expect that the slower solubilizers will need a higher concentration to reach saturation and solubilization of the bilayers, as well as display slower kinetics compared to a fast solubilizer. To compare the different solubilizers, one need to consider the two different lipids separately since the lipid identity will also affect the efficiency of solubilization. In Figure 10 we have plotted the observed mass ratio saturation limit, where we start to see solubilized structures in coexistence with non-solubilized structures, and the solubilization limit, where all vesicle structures have been converted into solubilized micellar units, as a function of temperature. Note that large lamellar sheet structures are not considered solubilized, as suggested also by Heerklotz,^10^ and so the rippled nano-flakes formed by mixtures of DPPC and TX-100 at low temperatures are considered as existing below the saturation limit.

For DPPC, we cannot separate between the point of saturation and solubilization for TX-100 since we cannot deduce coexistence for many of the scattering patterns. If we assume saturation and solubilization to occur at the same point, here we see that TX-100 reaches saturation at a higher concentration than that for the slow solubilizers at the lower temperatures while DDM and SDS both have saturation points that are very low across the temperature range. TX-100 reaches similar saturation limits to SDS and DDM at 30 and 45 °C, respectively. When looking at the concentrations at which full solubilization occurs, we see that this point occurs at lower concentrations for TX-100 than that for the slow solubilizers DDM and SDS in the temperature range 25–45 °C. Therefore, in terms of the completion of solubilization, our data from DPPC vesicles support the division of the detergents into fast and slow solubilizers at temperatures above ambient temperatures. Interestingly, however, when looking at the shorter chained lipid DMPC, we see that the concentration needed for complete solubilization by TX-100 increases as the temperature increases above the melting point of the limit, to the point where it crosses the concentration needed of DDM at 30 °C and has almost the same solubilization efficiency as SDS at 45 °C. Therefore, it seems that TX-100 is a less or equally efficient solubilizer than DDM or SDS above this temperature. In contrast, DDM and SDS barely show any temperature dependence in the efficiency of solubilization of DMPC. The results clearly show that different surfactants cannot be unambiguously divided into fast and slow solubilizers in term of the efficiency of solubilizing lipid vesicles since the temperature dependence of the solubilization is not necessarily monotonic. Although we do not have enough kinetic data to be able to compare at all the different temperatures, it does seem that at 20 °C TX-100 solubilizes the DMPC vesicles in a shorter timespan (<20 ms, less than the time resolution of the instrument) compared to the slow solubilizers DDM (between 20 and 110 ms) and SDS (∼10 s). In terms of times of solubilization at temperatures where the surfactants have comparable efficiency in terms of concentration, it therefore seems that the detergents do conform to their classifications as “fast” and “slow” solubilizers, with SDS being the slowest of the three.

### Charged versus Uncharged Surfactants

DDM and SDS are both considered slow solubilizers and share the same tail group but have very different headgroups. While DDM has quite a bulky but uncharged maltose headgroup, SDS has a very small but charged headgroup. In our results, the two surfactants behave quite similar, having limits of saturation that are very close for both the lipids. However, the SDS does seem to have a higher solubilization limit compared to DDM in most cases, suggesting that the lipid vesicles are more tolerant to insertion of the charged surfactant compared with DDM. The SDS headgroups are expected to experience some degree of shielding from each other by mixing into the zwitterionic lipid headgroups; thus, we can expect that the effective headgroup size is somewhat lower for the single SDS molecule in the bilayer compared to that in the pure SDS micelle. Molecular dynamics simulation of SDS in DMPC bilayers also found that the PC headgroups reorient their positively charged ends toward the negatively charged sulfate headgroup, thereby allowing SDS to penetrate deeper into the bilayer.^42^ This configuration could explain why the DMPC bilayers are more tolerant toward SDS insertion due to their smaller alkyl chains, while DPPC exerts a higher tolerance at the higher temperatures (Figure 10). In comparison, the DDM headgroup can perhaps not be accommodated in the same way, forcing the DDM headgroup to protrude out from the surface of the bilayer to a larger extent and causing a bigger packing mismatch that leads to more partial solubilization. Since DDM is uncharged, it might also be that it more easily flips across the bilayer causing solubilization from each leaflet. Another difference between DDM and SDS is in their effect on the bilayer at sub-solubilizing concentrations, where DDM induces formation of multilamellar structures. Although it has been reported previously that also SDS should be able to induce fusion,^52^ we do not see any increase in multi-lamellarity with SDS. We do however see an increase in the repeat distance of the already multilamellar fraction of DMPC vesicles (Figures 2 and S9), which potentially could help explain small increases in size that has been observed when adding SDS to PC vesicles previously.^28^ The solvent in our study consists only of Tris buffer (0.5 M), and since the aggregation behavior of SDS is highly sensitive to the ions in solution, it is highly possible that in solutions of higher ionic strength we might indeed see induced multi-lamellarity as in the case of DDM due to the screening of the headgroup charges.

### Effect of Temperature

The results from DMPC and DPPC seen together point to TX-100 increasing in the solubilization efficiency with temperature when the lipids are still in the gel phase, while after the transition temperature of the lipids, the solubilization efficiency decreases with temperature. The fact that the vesicles appear to remain intact at higher ratios above the transition temperature suggests that the phase of the lipids has a big role in the saturation of the bilayer. Less efficient solubilization of DMPC at temperatures above the transition temperature compared to that below was also observed when we studied the solubilization by the styrene-maleic acid polymer,^34^ and we may attribute the same causes here. The lipids in the liquid-crystalline phase have a higher tolerance toward insertion of detergent molecules into the bilayer structures, while in the gel phase, the insertion causes greater disruption in the packing of the lipids. At a low amount of insertion, this can be accommodated by the formation of the rippled phase, which occurs at the 8:1 ratio. As the amount of insertion increases, the lipid bilayer eventually fragments since the detergent is more easily accommodated at the rims of disc-like structures. The increase in the solubilization efficiency as the temperature is raised while the lipids are still in the gel phase can then be seen as simply arising from the increase in the thermal mobility and decrease in the cohesive energy of the bilayer at higher temperatures rather than due to changes in the lipid packing. The result also points toward continuously increased fluidity also as we move to higher temperatures after passing the transition temperature since the saturation limit is even higher at 45 °C than that at 30 °C.

### Effects of Partitioning: Vesicle Deformation and Increase in Size and Multi-Lamellarity

The three-step model of solubilizations claims that in the first stage where we have partitioning of surfactant into the bilayer, we should observe a size increase because of the increased surface area of the vesicles.^6^ Different mechanisms have been suggested to account for the observations of surplus size increase for different detergents, however, including fusion of vesicles, breakdown and reassembly of vesicles, and a disproportionation mechanism (increase lipid transfer from smaller to larger vesicles).^5,15,17,45,53^ In our study, we have seen that the structure of the aggregates before they are considered solubilized can be quite varied in nature. For partitioning of TX-100 below the melting temperature of the lipid, we see that at the lower temperatures, we have formation of large multilamellar nanoflakes, hypothesized by us based on a more extensive investigation to be the result of collapse of the vesicle structure into bi-lamellar flakes^48^ that can display a characteristic rippled bilayer structure at certain temperatures (Figure 5). This structure is characteristic for TX-100 among the detergents and conditions tested in this paper and has not been observed in any other system investigated by our group.

When the lipid is well above the transition temperature, such as DMPC at 45 °C, we instead see just an increase in the multi-lamellarity of the spherical vesicles (Figure 6). This behavior fits well with the previous observation of TX-100-induced vesicle growth of sonicated PC-vesicles above the transition temperature.^54^ DDM also induces an increase in the multi-lamellarity of DMPC vesicles but over a wider temperature range than TX-100. In general, increase in multi-lamellarity strongly supports a breakdown-reassembly mechanism to explain detergent-induced growth since fusion and disproportionation of lipids would yield only larger vesicles. The kinetic data of the DDM-DMPC system also show an intermediate elongated structure, also pointing to the breakdown-reassembly mechanism being correct.

SDS behaves differently from the other detergents in the sense that it does not disturb the overall vesicle structure to such a large extent when partitioning. As discussed above, SDS may disturb the packing less compared with the other detergents at low ratios due to its smaller headgroup and the shielding the negative charges by the PC-headgroups, making it less curvophilic in the bilayer compared to that in the pure micelle. We cannot exclude the possibility of vesicles structures that are intact but perforated as this cannot be seen from the small-angle scattering. Such perforated vesicles have been observed in the solubilization of lipid bilayers by ionic detergents previously,^26,43,55^ although notably it has only been tested in solution with added salts, and it is expected that charged surfactants will behave quite differently in the present of salt.^43^ From an energetic viewpoint, it seems more likely that the SDS would indeed partition in a way that could dilute the charge repulsion rather than form pore structures under our conditions.

### Transition from Mixed Vesicles to Mixed Micelles

The mechanism of transition from intact vesicles to small micellar units has been explored and discussed in large, and many different observations and suggestions have been made.^5,8,10,51,56^ For the most of the kinetic data, the transitions captured here have been gradual transitions where the partitioning has been followed by a gradual increase in the mixed micellar phase happening concurrent with a decrease in the population of mixed vesicles. It should be noted that these are perhaps the exceptions since much of the kinetics was too fast to measure, and so, we cannot say anything about the kinetic pathway of these. This pertains to solubilization of DMPC with the fast solubilizer TX-100 at all temperatures except 10 °C where we could see the kinetics over the course of 1 s and with DDM when the detergent concentration is above the solubilization limit, where the kinetics would be over within the first 100 ms. For SDS, we can see the end of the solubilization happening within 20 s. The situation was similar to DPPC when reaching temperatures above 30 °C, whereas below this temperature, we would only have partial or no solubilization, and kinetics would be very slow, in the range of several hours. The only exception is when TX-100 is added in an extreme excess at low temperature or at a low ratio at 30 °C, where we see a very slow solubilization occurring over the course of 3 min.

In the case of TX-100 solubilization of DPPC, the time scale of the process at different temperatures overlaps despite very different amount of TX-100 being needed (Figure 9c). This suggest that the mechanism of solubilization must be similar even though the process yields different structures due to the very different amounts of TX-100 used in the two cases. DMPC at 10 °C, however, seems to display a different type of kinetics, with a very abrupt solubilization into bicelles. This is reminiscent of what was observed by Sudbrack et al. with optical microscopy of POPC GUVs where TX-100 at super-solubilizing concentrations first caused an increase in the bilayer area followed by the vesicles becoming perforated like lace fabric and the bilayer structure then gradually vanishing.^22^ A similar burst-type kinetics seems to be present for SDS solubilization of DMPC at 20 °C (Figure 7c), although there we have less time resolution. In contrast, solubilization by DDM of DMPC is too fast to be observed at 20 °C. Taken together, the results suggests that the cohesiveness of the bilayer is a good predictor for the type of solubilization kinetics we will observe; the DPPC tail group has more attractive interactions (cohesive energy) than DMPC due to the longer acyl chain and stronger van der Waals interactions. The longer-chained lipid displayed gradual and slow solubilization in all the conditions tested here; however, only conditions well below the melting temperature were tested (10–30 °C). We cannot exclude the possibility that the kinetics of the DPPC solubilization would change if the temperature was closer to the transition point, but it still seems that the shorter chained DMPC is more disposed to burst-type kinetic even well below the transition temperature (at 10, 14 °C below transition point). We also saw that DPPC bilayers are exceptionally resistant to partitioning of TX-100 at low temperatures since most of the TX-100 are still in their pure micellar form. Since it requires a very high concentration of micelles to actually start solubilization, it seems likely that the process here is a slow extraction of lipids from the bilayer to the micelles with little partitioning of detergent into the vesicle structures occurring, rather than a saturation of the bilayer followed by solubilization. The fact that the solubilization and saturation limits for this system overlap also support this hypothesis (Figure 10).

Particularly for SDS, Igarashi et al. suggested that at concentrations above the CMC, the SDS micelles themselves must interact with the bilayer and cause local instabilities to explain their observations of what they describe as a projection-disruption type solubilization at low concentrations and burst motion type at higher concentrations.^57^ Others have also observed a burst-type of solubilization with SDS occurring around the CMC of the surfactant.^22^ Indeed, for DMPC at all temperatures and DPPC at 45 °C, we also observe a change in behavior around the supposed CMC of SDS of 2.3 mg/mL, where we start to see solubilization of the vesicle structure; however, SAXS measurements of the pure SDS solution show that micelles are still present at even lower concentrations, and the CMC as estimated from the SAXS measurements is around 0.7 mg/mL. This then raises the question as to why these micellar units would not interact directly with the bilayer casing partial solubilization in the case of DMPC and should point against the direct micellar contact mechanism. The concentration of micellar units is, however, quite low at this concentration, which could also play a part if a critical number of micellar disruptions are necessary to facilitate solubilization. For DPPC, on the other hand, we do indeed observe partial solubilization at the lower concentrations when below the melting temperature of the lipids, starting at the 0.65 mg/mL concentration for the two lowest temperatures. This is close to the estimated CMC from the SAXS measurements, and so it might suggest that similar to TX-100, SDS also relies on a slow extraction of lipids through micelles in the case where we are well below the melting temperature of the lipid, whereas closer to the transition temperature a burst-like mechanism dominates.

### Structure of Solubilized Aggregates

It is generally found that at very high surfactant concentrations, the solubilized structure is similar to the spheroidal structure of the pure surfactant micelles. However, the solubilized lipid/surfactant mixtures may also form disc or rod/thread-like micelles when solubilization occurs at lower surfactant concentrations.^41,58^ The solubilized aggregates in our study showed a variety of different structures depending much on the detergent, lipid type, and temperature. The disc-shaped bicelles were observed to some extent in all the different lipid/detergent combinations tested in this work. Johansson et al. argue that it is the bending rigidity of the assembly that determines whether a discoidal or thread-like mixed micelle is formed.^41^ They also found that DPPC only formed discoidal particles with all the surfactants they tested. Their results and conclusions are quite in line with our results from TX-100 since discoidal particles are predominant below the melting temperature where the bending rigidity of the bilayers is expected to be higher and the miscibility lower, thereby facilitating the segregation of the detergent to the rim of the disc. In addition, the charged surfactant SDS is expected to increase the bending rigidity of the bilayer due to the raised surface charge density,^41^ which could explain why in the case of SDS solubilization of DPPC the discoidal particles are predominant at the higher temperatures. Again, in accordance with the idea that the bending rigidity is determinant of the aggregate structure, we see that above the melting temperature of DPPC we have solubilization into rod/worm-like micelles with DDM, while below we only have partial solubilization into discoidal structures. For DMPC, we observed rod/worm-like micelles for all temperatures at high enough DDM concentration. It is, however, plausible that the increased partitioning of DDM into DMPC due to the lower cohesiveness causes a larger shift in the melting temperature of DMPC, and that this would explain the formation of rods/worms even at the lower temperatures. In our study, we do find that at the concentrations before solubilization for TX-100 and SDS, we do have elongated structures present, although the scattering is not perfectly modeled by rodlike structures and should potentially rather be characterized by complex mixtures with longer, flexible bilayered particles or potentially ribbon-like micelles that are intermediate between the vesicle structure and disc structure, which have also been observed in other studies.^24,25,41,46,55,59^

## Conclusions

This paper has reported on the static and kinetic SAXS data from three very different but popularly used surfactants on two different lipid systems at a range of ratios and temperatures. The aim of the study was to shed light on the variety of structures that can occur during the solubilization process and how it relates to their mechanisms of action. We find that for the temperature, dependence of solubilization is not necessarily monotonic, and this makes it difficult to unambiguously divide different surfactants into fast and slow solubilizers in terms of the mass efficiency. At conditions where the surfactants do have comparable efficiency in terms of concentration, however, it does seem that the kinetics do follow the classification of slow and fast solubilizers regarding the time it takes to solubilize the membrane. For TX-100 particularly, the solubilization efficiency increases with temperature up to the melting temperature of the lipid being solubilized, after which it decreases with further increase in temperature. SDS is the only surfactant that does not induce large-scale deformations of the lipid vesicles at sub-solubilizing concentrations. TX-100 is seen to be able to induce a collapse and “ridging” of the bilayers at low temperatures unless very high concentrations are used. Both DDM and TX-100 cause an increase in the multi-lamellarity of the vesicles at temperatures above the phase transition of the lipid, and for DDM, the kinetics seem to suggest that this occurs via open vesicular intermediates. From the kinetic results of the longer chained lipid compared to that of the shorter chained lipid, it seems that the cohesive energy of the bilayer plays an important role in the solubilization mechanism of the vesicles, more so than the identity of the detergent. The bending rigidity of the lipid/surfactant mixture is an important factor determining the structure of the solubilized aggregate. Overall, this article presents a wide range of new structural and kinetic data on the solubilization process, which is expected be an inspiration for further exploration of these types of systems and useful in guiding surfactant applications.

## Supporting Information Available

Density measurements, details of the analytical SAXS models, all SAXS curves, obtained fit parameters, results from other analysis, and cryo-TEM images (PDF)

## Abbreviations

SDS: sodium dodecyl sulfate
DDM: *n*-dodecyl-β-d-maltoside
TX-100: Triton X-100
DPPC: 1,2-dipalmitoyl-*sn*-glycero-3-phosphocholine
DMPC: 1,2-dimyristoyl-*sn*-glycero-3-phosphocholine
POPC: 1-palmitoyl-2-oleoyl-*glycero*-3-phosphocholine
CMC: critical micellar concentration
SAXS: small-angle X-ray scattering
TEM: transmission electron microscopy
NMR: nuclear magnetic resonance
