# Supplementary material for: Pathways of Membrane Solubilization: A Structural Study of Model Lipid Vesicles Exposed to Classical Detergents
Source: Langmuir. 2023 Mar 9;39(11):3914–33. doi: 10.1021/acs.langmuir.2c03207 (PMC10035035; doi:10.1021/acs.langmuir.2c03207)
Supplement: Supplementary file 1 — la2c03207_si_001.pdf [file la2c03207_si_001.pdf]

Pathways of Membrane Solubilization: A Structural Study of Model Lipid Vesicles Exposed to Classical Detergents

Victoria Ariel Bjørnestad[†] and Reidar Lund[*,†].

† Department of Chemistry, University of Oslo, Sem Sælandsvei 26, 0371 Oslo, Norway

Contents

S1: Density measurements 3
S2: Analytical scattering models 4
S2.1: Vesicle model 4
Pure lipid vesicles 4
Mixed lipid:surfactant vesicles 6
S2.2: Rod and disc/bicelle model 8
S2.3: Ellipsoidal and triaxial micelle model 10
S2.4: Triton X-100 micelle model 10
S2.5: Coexistence models 11
S3: Analysis of static scattering data 11
S3.1: Pure lipid vesicles and surfactant micelles 11
S3.2: DPPC:SDS mixtures 16
S3.3: DMPC:SDS mixtures 24
S3.4: DPPC:DDM mixtures 28
S3.5: DMPC:DDM mixtures 34
S3.6: DPPC:TX-100 mixtures 37
S3.7: DMPC:TX-100 mixtures 41
S4: Analysis of kinetic SAXS data 44
S4.1: DPPC:SDS mixture at 20°C 44
S4.2: DMPC:SDS mixture at 20°C 45
S4.3: DPPC:DDM mixture at 20°C 46
S4.4: DMPC:DDM mixture at 20°C 47
S4.5: DPPC:TX-100 mixtures at 10°C 47
S4.6: DMPC:TX-100 mixture at 20°C 49
S4.7: DPPC:TX-100 mixture at 30°C 50
S4.8: DMPC:TX-100 mixture at 10°C 51
S5: Cryo-TEM images 52
S6: References 53

S1: Density measurements

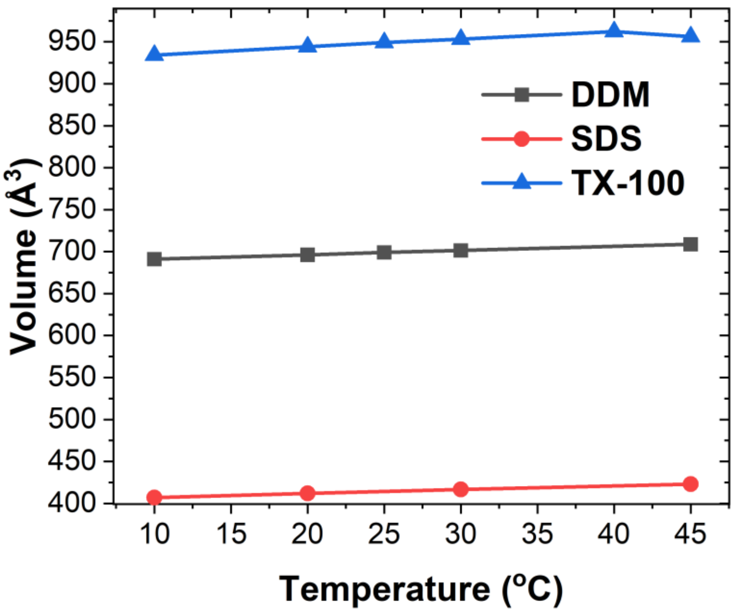

Figure S1: *Molecular volume calculated from the measured densities as a function of temperature for the three different surfactants. The density* d *is given in Table S1. The volume was then calculated as* $V = M_w/(N_a d)$ *where* M_w *is the molar mass of the solute and* N_a *is Avogadro's constant.*

Table S1: *Measured densities of the three surfactants used: SDS, DDM and Triton X-100. These were calculated from measurements of the solvent* (d_0) *and solution densities* (d_{sol})*:* $d = wd_{sol}d_0/(d_0 + (w-1)d_{sol})$*, where w is the weight fraction of solute in solution.*

Measured densities of surfactants [g/cm^3]			
**Temperature [°C]**	**SDS**	**DDM**	**Triton X-100**
10	1.17705	1.22720	1.11125
20	1.16253	1.21781	1.09941
25	-	1.21309	1.09380
30	1.14953	1.20899	1.08876
40	-	-	1.07831
45	1.13215	1.19646	1.08513

S2: Analytical scattering models

S2.1: Vesicle model

Pure lipid vesicles

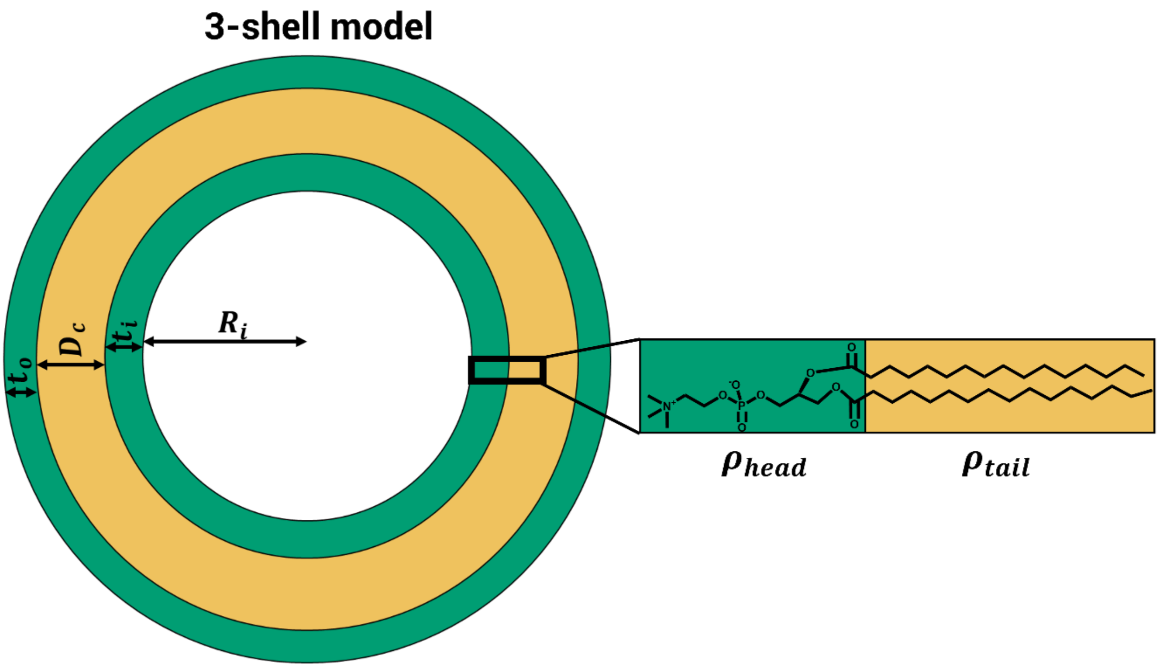

Figure S2: Illustration of the 3-shell model. t_o = thickness of outer head shell, D_c = thickness of tail shell, t_i = thickness of inner head shell, R_i = inner radius, ρ_{head} = SLD headgroup and ρ_{tail} = SLD tailgroup. Note that for mixed surfactant:lipid vesicles the SLD are calculated from the molar ratios of lipid:surfactant in the aggregate as described in equations S17 and S18. The interfaces between the shells are smeared by disorder parameters σ_i.

The SAXS measurements of the DMPC and DPPC vesicle were analysed using the 3-shell model, illustrated in Figure S2, where 1 shell accounts for the hydrocarbon region of the bilayer and 2 surrounding shells account for the headgroup regions on the outer and inner leaflet. The scattering amplitude is expressed as the sum of the form factors 3 concentric shells:

$$A(Q)_{3-shell} = \sum_{i=1}^{i=3} \Delta\rho_i \cdot V_i \cdot A(Q, R_i, R_{i-1})_{shell} \qquad (S1)$$

where V_i are the different shell volume:

$$V_i = \frac{4\pi}{3}\left(R_i^3 - R_{i-1}^3\right) \qquad (S2)$$

and $\Delta\rho_i$ is the contrast of that respective shell, defined as $\Delta\rho_i = \rho_i - \rho_0$, where ρ_0 is the scattering length density (SLD) of the solvent (Tris buffer in all cases here) and ρ_i the SLD of the respective shell. The SLD for the hydrocarbon region is calculated from the number of electrons, Z_{tail}, and the volume of a single lipid tail, V_{tail}:

$$\rho_{HC} = \frac{Z_{tail}}{V_{tail}} \cdot r_0 \qquad (S3)$$

with r_0 being the Thompson scattering length. In the headgroup shells, the hydration is also considered when calculating the SLDs. The SLD for a particular shell *i*=inner|outer is:

$$\rho_i = \left(1 - f_{w,outer}\right) \cdot \rho_{head} + f_{w,i} \cdot \rho_0 \qquad (S4)$$

And the parameter $f_{w,i}$ is the calculated fraction of water in that shell:

$$f_{w,i} = 1 - \frac{V_{head} \cdot P_{agg} \cdot 0.5}{V_i} \qquad (S5)$$

where the aggregation number P_{agg} is calculated from the how many lipid tail groups of volume V_{tail} fit into the tail group shell V_c calculated as in equation S2:

$$P_{agg} = \frac{V_c}{V_{tail}} \qquad (S6)$$

When using a common shell for the hydrophobic group, we need to make an assumption about the aggregation number in the outer and inner leaflet to calculate the hydration of the headgroup shells. Here, we have used the simplification that half of the lipids are in each leaflet; one might want to try other assumptions, however, we have found that the difference in the fits (tested by the exact calculation using of two shells from the hydrocarbon region as described in the next section) is completely insignificant in our fits. The headgroup SLD is calculated in the same way as it was for the tailgroup:

$$\rho_{head} = \frac{Z_{head}}{V_{head}} \cdot r_0 \qquad (S7)$$

The scattering amplitude for each shell is defined as:

$$A(Q)_{shell} = \frac{\frac{4 \cdot \pi \cdot R_i^3}{3} \cdot A(Q,R_i)_{sphere} \cdot \exp\left(-\frac{Q^2 \cdot \sigma_i^2}{2}\right) - \frac{4 \cdot \pi \cdot R_{i-1}^3}{3} \cdot A(Q,R_{i-1})_{sphere} \cdot \exp\left(-\frac{Q^2 \cdot \sigma_{i-1}^2}{2}\right)}{V_{i-1}} \qquad (S8)$$

where R_i and R_{i-1} represent the outer and inner radii of the shell, while σ_i and σ_{i-1} are the disorder parameters for the inner and outer boundaries of the shell. $A(Q,R)_{sphere}$ is the spherical form factor and is defined as:

$$A(Q)_{sphere} = \frac{3 \cdot (\sin(Q \cdot r) - Q \cdot r \cdot \cos(Q \cdot r))}{(Q \cdot r)^3} \qquad (S9)$$

To account for the multilamellarity of the liposomes, we used the Modified Caillé theory structure factor, implemented the same way as in the SASfit software, with:

$$S_{N_k,MC} = N_{diff} + N_k + 2 \cdot \sum_{k-1}^{N_k-1} (N_k - k) \cos(kQd) \exp\left(\left(-\frac{d}{2\pi}\right)^2 Q^2 \eta_1 \gamma\right) (\pi k)^{\left(\frac{d}{2\pi}\right)^2 Q^2 \eta_1} \qquad (S10)$$

where N is the average number of bilayers in the multilamellar structure, N_{diff} is the number of uncorrelated scattering bilayers, d is the mean stacking separation, γ is the Euler's constant and $\eta_1$is the Caillé parameter which accounts for the bilayer fluctuations. The final structure factor becomes:

$$S_{MC}(Q, N_k) = (1 - w) S_{[N_k],MC}(Q) + w S_{[N_k]+1,MC}(Q) \qquad (S11)$$

where $w = N_k - [N_k]$.

To account for polydispersity in the size of the different stacks, we use an average over a Gaussian distribution where σ is the standard deviation of the Gaussian-weighted distribution, making:

$$S(Q) = \sum_{N_k-N-2\sigma}^{N_k+2\sigma} x_k(N_k)S_{MC}(Q, N_k) \tag{S12}$$

where

$$x_k = \frac{1}{\sigma\sqrt{2\pi}}\exp\left(-\frac{(N_k - N)^2}{2\sigma^2}\right) \tag{S13}$$

The final scattering intensity is then calculated as:

$$\langle I(Q)\rangle = \left[\frac{\phi}{\langle V\rangle}\int_0^\infty f(R_{tot}) \cdot A(Q)_{3-shell} \cdot A(Q)_{3-shell}\, dr\right] \cdot \left(f_{uni} + (1 - f_{uni}) \cdot S(Q)\right) \tag{S14}$$

Mixed lipid:surfactant vesicles

The model used for the mixed vesicles of surfactant and lipids is exactly as described above, but with the volumes and thus SLDs of the shell calculated from the ratio of surfactant:lipid, $r_{surfactant}$, that exists in the vesicle:

$$r_{surfactant} = \frac{n_{surfactant}}{n_{lipids}} \tag{S15}$$

The surfactant tail group is always assumed to be fully inserted into the hydrocarbon shell, while the surfactant headgroup is always assumed to mix with the lipid headgroups. Thus, the volumes of the mixed lipid-surfactant pseudomolecules become:

$$V_{core} = V_{lipid\ tail} + V_{surfactant\ tail} \cdot r_{surfactant} \tag{S16}$$

$$V_{head} = V_{lipid\ head} + V_{surfactant\ head} \cdot r_{surfactant} \tag{S17}$$

And the corresponding SLDs were calculated as:

$$\rho_{core} = \frac{Z_{lipid\ tail} + Z_{surfactant\ tail} \cdot r_{surfactant}}{V_{core}} \cdot r_0 \tag{S18}$$

$$\rho_{head} = \frac{Z_{lipid\ head} + Z_{surfactant\ head} \cdot r_{surfactant}}{V_{head}} \cdot r_0 \tag{S19}$$

In the case of DDM an extra shell was included to make up two shells in the hydrocarbon region to account for possible asymmetry in the insertion of detergent. Otherwise, the SLDs and volumes were modified to that of a mixed lipid-surfactant pseudomolecules. This gives the following pseudovolume groups:

$$V_{core\_inner} = V_{lipid\ tail\ outer} + V_{surfactant\ tail} \cdot r_{surfactant\ outer} \tag{S20}$$

$$V_{core\_outer} = V_{lipid\ tail\ outer} + V_{surfactant\ tail} \cdot r_{surfactant\ inner} \tag{S21}$$

$$V_{head\_outer} = V_{lipid\ head} + V_{surfactant\ head} \cdot r_{surfactant\ outer} \tag{S22}$$

$$V_{head\_inner} = V_{lipid\ head} + V_{surfactant} \cdot r_{surfactant\ inner} \quad (S23)$$

where $r_{surfactant\ outer}$ and $r_{surfactant\ inner}$ are the ratios of surfactant:lipid in the outer and inner leaftlets, respectively. These ratios are found from the total amount of surfactant and lipid in solution and by the asymmetry parameter, f_{symm}, which determined how much goes to each leaflet.

$$r_{surfactant\ inner} = f_{symm} \cdot \frac{n_{surfactant}}{0.5 \times n_{lipids}} \quad (S24)$$

$$r_{surfactant\ outer} = \left(1 - f_{symm}\right) \cdot \frac{n_{surfactant}}{0.5 \times n_{lipids}} \quad (S25)$$

Here, some assumption must be made about the distribution of lipids across the leaflet needs to be made, and the simplified assumption that half the lipids are in each leaflet is used. We note that for small vesicles this is not exactly the case, and thus other authors might consider using a different assumption if higher accuracy in this parameter is required. The same procedure is used for determining the number of electrons in each pseudogroup and thereby used to calculate the scattering length densities and contrast of each pseudogroup:

$$\rho_{core\_outer} = \frac{Z_{lipid\ tail\ outer} + Z_{surfactant\ tail} \cdot r_{surfactant\ outer}}{V_{core_{outer}}} \cdot r_0 \quad (S26)$$

$$\rho_{core\_inner} = \frac{Z_{lipid\ tail\ inner} + Z_{surfactant\ tail} \cdot r_{surfactant\ inner}}{V_{core_{inner}}} \cdot r_0 \quad (S26)$$

$$\rho_{head\_outer} = \frac{Z_{lipid\ head} + Z_{surfactant\ head} \cdot r_{surfactant\ outer}}{V_{head_{outer}}} \cdot r_0 \quad (S28)$$

$$\rho_{head\_inner} = \frac{Z_{lipid\ head} + Z_{surfactant\ head} \cdot r_{surfactant\ inner}}{V_{head_{inner}}} \cdot r_0 \quad (S29)$$

The aggregation number is the calculated from how many tail groups fit into each tail group shell, of volume $V_{c,inner}$ and $V_{c,outer}$:

$$P_{agg,inner} = \frac{V_{core\ inner}}{V_{lipid\ tail\ inner}} \quad (S30)$$

$$P_{agg,outer} = \frac{V_{core\ outer}}{V_{lipid\ tail\ outer}} \quad (S31)$$

And the corresponding fractions of water for the inner and outer headgroup shells are then calculated as in equation S5 using the corresponding aggregation numbers belonging to the shell group.

S2.2: Rod and disc/bicelle model

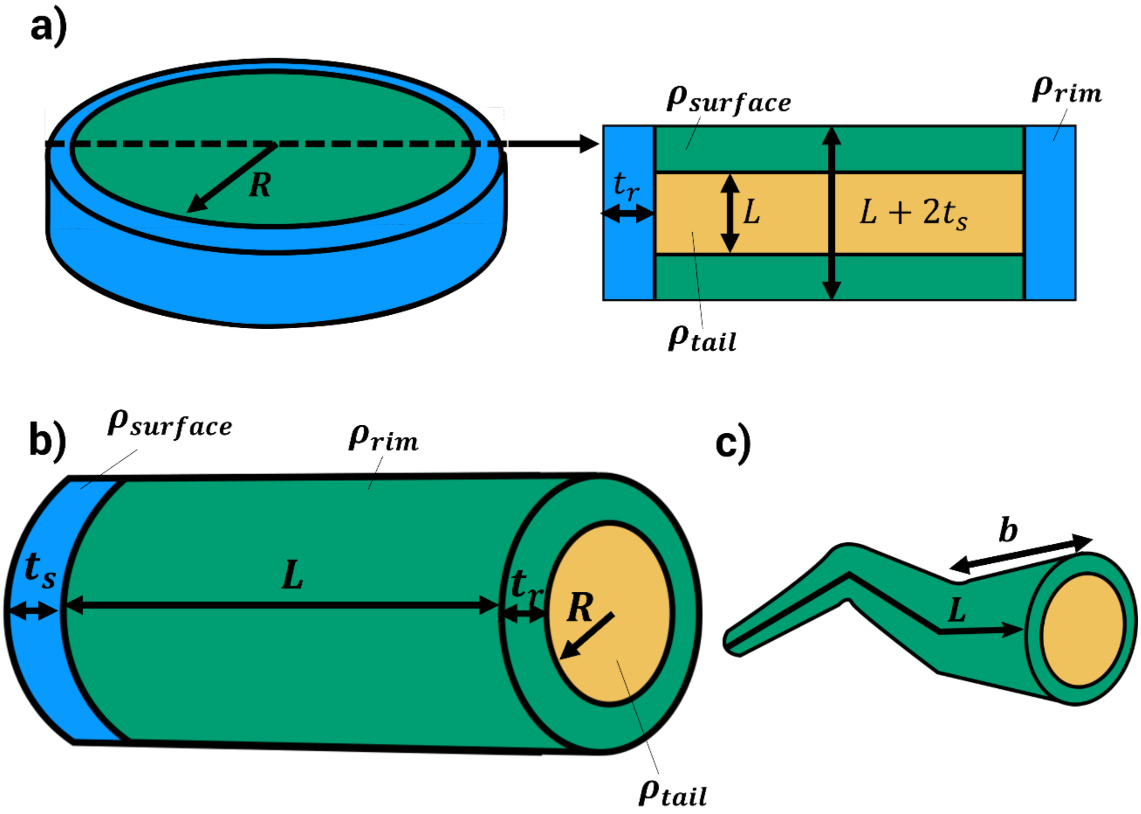

Figure S3: Illustration of the a) disc model with cross-section shown on the right, b) rod model (cross-section) and c) additional parameter to the rod in the worm model. L =length of rod, R =radius of rod, t_s =thickness of headgroup in surface/cap, t_r =thickness of headgroup in rim/rod length, b =statistical segment length of rod, ρ_{head} = SLD headgroup and ρ_{tail} = SLD tailgroup. The SLDs are calculated as described in equations S34-S36. Color change between disc and rod simply indicate where we expect the surplus of lipids (green) to remain in the different structures. The interfaces between the structures are smeared by a disorder parameter σ.

The rod/disc model is illustrated in Figure S3 both as the disc (Figure S3a) and as the rod (Figure S3b)) and the additions parameter that is added upon worm formation (Figure) The contrast in the rod/disc model is defined in the same manner as for the mixed lipid vesicles in S2.1 above in terms of mixtures between surfactant and lipid.

The scattering amplitude of a cylinder of length L_i and radius R is defined as

$$A_{cyl}(Q,\alpha) = \frac{2 \cdot \sin(Q \cdot L_i \cdot \cos(\alpha)/2)}{Q \cdot L_i \cdot \cos(\alpha)/2} \cdot \frac{J_1(Q \cdot R_i \cdot \sin(\alpha))}{Q \cdot R_i \cdot \sin(\alpha)} \qquad (S31)$$

A simple rod/disc model for micelles could be defined by 2 cylinders, the core cylinder, and the shell cylinder. For the model to account for asymmetry in the rim and surface of the shell, we instead define 3 cylinders, consisting of the core cylinder (consisting of lipid/surfactant tail groups), the core + surface headgroups cylinder (denoted core + dL) and finally the core + surface headgroups + rim headgroups (denoted core + dL + dR). The different lengths will then give the different values for H and R to input into in equation S30 to yield A_{core}, $A_{core+dL}$ and $A_{core+dL+dR}$. The final form factor for the cylindrical micelle will then be:

$$I_{cylinder}(Q) = n_z \cdot \int_0^{2\pi} (\mathrm{F_{core}} + F_{core+dL} + F_{core+dL+dR})^2 \sin(\alpha)\, d\alpha \; \cdot \exp\left(-\frac{Q^2 \cdot \sigma^2}{2}\right)$$

$(S32)$

where n_z is the number density of the particles, σ is a disorder parameter to smear the core-shell interface and the different terms are

$$\mathrm{F_{core}} = \Delta\rho_{tail} \cdot V_{core} \cdot A_{core}(Q)$$

$(S33)$

$$\mathrm{F_{core+dL}} = \Delta\rho_{dL} \cdot \left(V_{core+dL} \cdot A_{core+dL}(Q) - V_{core} \cdot A_{core}(Q)\right)$$

$(S34)$

$$\mathrm{F_{core+dL+dR}} = \Delta\rho_{dR} \cdot \left(V_{core+dL+dR} \cdot A_{core+dL+dR}(Q) - V_{core+dL} \cdot A_{core+dL}(Q)\right)$$

$(S35)$

The contrast terms $\Delta\rho_i$ are defined as $\Delta\rho_i = \rho_i - \rho_0$, where the various ρ_i are as follows:

$$\rho_{core} = \frac{Z_{lipid\ tail} + Z_{surfactant\ tail} \cdot r_{surfactant}}{V_{lipid\ tail} + V_{surfactant\ tail} \cdot r_{surfactant}} \cdot r_0$$

$(S36)$

$$\rho_{dR} = \frac{Z_{lipid\ tail} \cdot f_{LdR} + Z_{surfactant\ tail} \cdot r_{surfactant} \cdot f_{SdR}}{V_{lipid\ tail} \cdot f_{LdR} + V_{surfactant\ tail} \cdot r_{surfactant} \cdot f_{SdR}} \cdot r_0$$

$(S37)$

$$\rho_{dL} = \frac{Z_{lipid\ tail} \cdot (1 - f_{LdR}) + Z_{surfactant\ tail} \cdot r_{surfactant} \cdot (1 - f_{SdR})}{V_{lipid\ tail} \cdot (1 - f_{LdR}) + V_{surfactant\ tail} \cdot r_{surfactant} \cdot (1 - f_{SdR})} \cdot r_0$$

$(S38)$

where $r_{surfactant}$ is the number of surfactants per lipid present in the micelle, and $f_{LdR}$and f_{SdR} are the fraction of the lipid headgroups and surfactant tailgroups respectively that go to the rim rather than the surface of the disc.

When H>>R the structure is considered a rod. For the rod/worm-like micelles, $dL = dR$ and $\rho_{dR} = \rho_{dL} = \rho_{shell}$ in all cases.

For the worm-like micelles we use the fact that for long cylinders the form factor can be decoupled into the contributions from the cross-section and the length of the cylinder, with the amplitude of the cross-section being

$$A_{cr}(QR) = \frac{2J_1(QR)}{QR}$$

$(S39)$

and the amplitude in the longitudinal direction is given by the form factor for a semi-flexible chain P_{chain} as reported in the works of Pedersen et. al. [1]
Including polydispersity, this gives the final expression

$$I_{worm}(Q) = \frac{n_z \int_{L_{min}}^{L_{max}} L^2 f(L) P_{CS}(Q,L) dL}{\int_{L_{min}}^{L_{max}} L^2 f(L) dL}$$

$$(S40)$$

where

$$P_{CS}(Q,L) = \left(\Delta\rho_{shell} A_{cr}(QR_{tot}) + (\Delta\rho_{core} - \Delta\rho_{shell}) \cdot V_{core} A_{cr}(QR_{core})\right)^2 \cdot P_{chain}$$

$$(S41)$$

S2.3: Ellipsoidal and triaxial micelle model

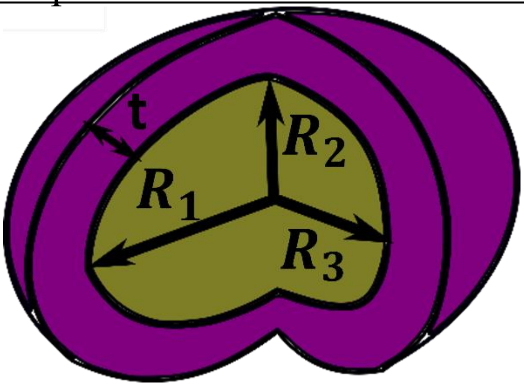

Figure S4: Illustration of the triaxial model and the parameters

The ellipsoidal and triaxial models for concentric shells are already well described by Pedersen[2]. A hard-sphere structure factor was used to account for the repulsion between negatively charges micelles of SDS or mixed aggregates of lipid:SDS. For mixed micelles, the core and head shell scattering length densities were calculated as

$$\rho_{core} = \frac{Z_{lipid\ tail} + Z_{surfactant\ tail} \cdot r_{surfactant}}{V_{lipid\ tail} + V_{surfactant\ tail} \cdot r_{surfactant}} \cdot r_0$$

$$(S42)$$

$$\rho_{shell} = \frac{Z_{lipid\ tail} \cdot f_{LdR} + Z_{surfactant\ tail} \cdot r_{surfactant}}{V_{lipid\ tail} \cdot f_{LdR} + V_{surfactant\ tail} \cdot r_{surfactant}} \cdot r_0$$

$$(S43)$$

S2.4: Triton X-100 micelle model

For the pure Triton X-100 micelles, a spherical micelle model with a density gradient and blob scattering as presented by Lund et. al.[3] was used with one modification: the Triton X-100 tail group was allowed to intermix into the headgroup shell via a fraction, f_{core}, so that the SLD of the shell was changed accordingly:

$$\rho_{shell} = \frac{Z_{head} + Z_{tail} \cdot (1 - f_{core})}{V_{head} + V_{tail} \cdot (1 - f_{core})} \cdot r_0$$

$$(S44)$$

The aggregation number was fitted and thus the core radius was calculated as

$$R_c = \left(\frac{3}{4\pi} \cdot Vtail \cdot P \cdot f_{core}\right)^{1/3}$$

$(S45)$

S2.5: Coexistence models

Wherever we have reported coexistence between vesicles structures and micellar structures, this has simply been done by having two parameters, $f_{lipid\_micelle}$ and $f_{surfactant\_micelle}$ to vary the concentration of each of the components that should be used to calculate the scattering intensity arising from each structure separately. These intensities are then added together.

S3: Analysis of static scattering data

S3.1: Pure lipid vesicles and surfactant micelles

The scattering data from the references of the pure lipid vesicles were analysed by the vesicle model as described in section S2.1. Figure S2 presents representative results of such measurements, with the corresponding fit parameters presented in Table S2 and S3. Note that new reference samples were taken for each preparation of lipid vesicles, since preparations tend to vary slightly in degree of multilamellarity and size. Figure S2 c) displays the fit parameters that accounts for most of the change in scattering between different temperatures.

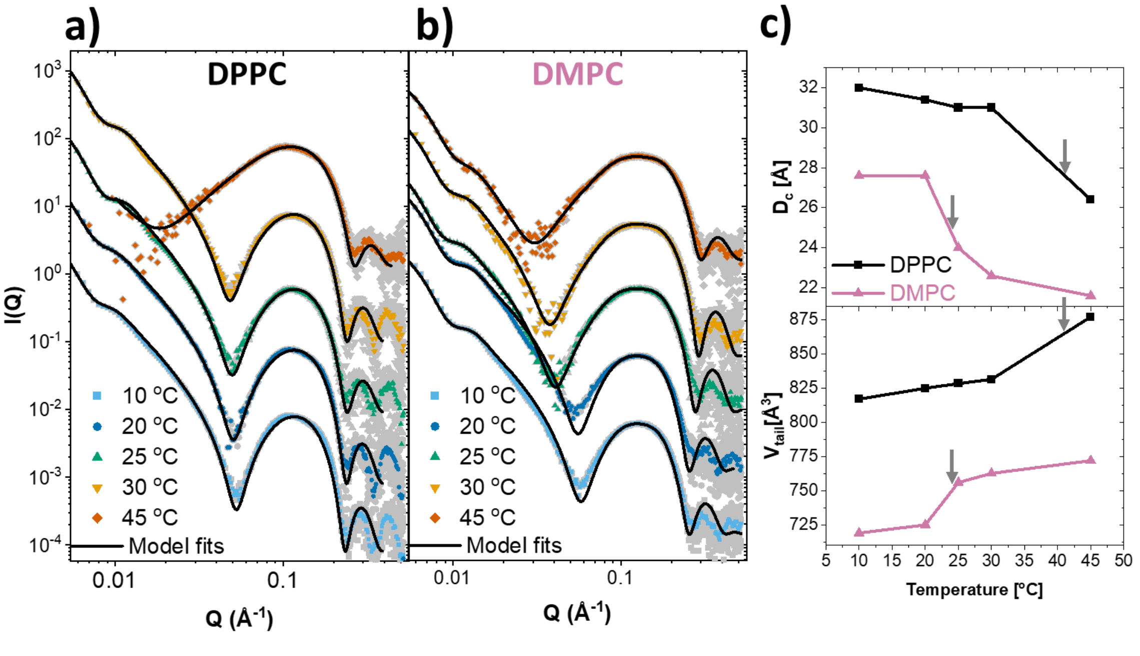

Figure S5: *SAXS measurements at different temperatures with model fits of a) DPPC and b) DMPC. c) Changes in the bilayer thickness (D_c) and tail volume (V_{tail}) derived from model fits as a function of temperature. Arrows indicate the transition temperature of the lipid.*

Table S2: *Model fit parameters from analysis of SAXS data from samples of 2.5mg/ml DMPC vesicles analysed using the 3-shell model*

**Model fits of SAXS data from 2.5mg/ml DMPC vesicles**					
	10°C	20°C	25°C	30°C	45°C

Vesicle model					
Average radius [Å]	245 (269)	250(277)	275	260(307)	240(290)
Thickness of i/o tail shell [Å]	13.8 (13.6)	13.8(13.8)	12.0	11.3(11.2)	10.8(10.7)
Thickness of i/o head shell [Å]	11.0(11.3)/ 7.5(7.8)	11.0(11.0)/ 7.5(7.5)	10.0/ 9.0	10.0(10.0)/ 9.0(9.0)	10.0(10.0)/ 9.0(0.9)
Smearing (i/o tail region) [Å]	3.8(3.5)	4.0(3.8)	1.8	2.0(2.0)	1.8(1.8)
Smearing (i/o head region) [Å]	2.5(2.5)/ 2.0(1.0)	2.5(2.5)/ 2.0(2.0)	2.0/ 1.0	2.0(2.0)/ 1.0(1.0)	2.0(2.0)/ 1.0(1.0)
Hydration of i/o head shell*	0.330(0.369)/ 0.253(0.288)	0.337(0.353)/ 0.257(0.257)	0.409/0.476	0.447(0.466)/ 0.511(0.511)	0.475(0.494)/ 0.541(0.539)
Volume lipid (o/i) [$Å^3$]	1150(1154)	1056(1064)	1087	1094(1099)	1103(1107.4)
Fraction of unilamellar vesicles	0.90(0.82)	0.90(0.82)	0.90	0.90(0.85)	0.90(0.85)
Nu	2(1)	2(1)	0	0(0)	0(0)
Number of bilayers	2(1)	2(1)	2.0	2.0(2.0)	2.0(2.0)
Sigma_d	10(1)	10(1)	10	10(1)	10(1)
Distance between lamella [Å]	69(69)	69(69)	69	66(61)	66(61)
Eta	0.3(0.3)	0.3(0.3)	0.3	0.3(0.3)	0.3(0.3)
Polydispersity (gaussian model)	0.33(0.30)	0.33(0.30)	0.33	0.33(0.24)	0.33(0.26)
SLD head [cm^{-2}]	1.396E+11 (1.396E+11)	1.396E+11 (1.396E+11)	1.396E+11 (1.396E+11)	1.396E+11 (1.396E+11)	1.396E+11 (1.396E+11)
SLD tail [cm^{-2}]	8.230E+10 (8.185E+10)	8.162E+10 (8.073E+10)	7.828E+10	7.759E+10 (7.705E+10)	7.665E+10 (7.622E+10)

Table S3*: Model fit parameters from analysis of SAXS data from samples of 2.5mg/ml DPPC vesicles analysed using the 3-shell model*

Model fits of SAXS data from 2.5mg/ml DPPC vesicles					
	10°C	20°C	25°C	30°C	45°C
Vesicle model					
Average radius [Å]	300(237)	285(237)	285	280(261)	280(261)
Thickness of i/o tail shell [Å]	16.0(15.8)	15.7(15.4)	15.5	15.5(14.9)	13.2(12.9)
Thickness of i/o head shell [Å]	9.0(9.0)/ 7.5(7.5)	9.0(9.6)/ 7.5(6.8)	9/7.5	9.0(9.6)/ 7.5(7.8)	9.0(9.6)/ 7.5(6.8)

Smearing (i/o tail region) [Å]	4.8(4.5)	5.0(3.1)	5.0	5.0(3.4)	4.2(3.2)
Smearing (i/o head region) [Å]	4.0(4.0)/ 3.0(3.0)	4.0(4.0)/ 3.0(3.0)	4.0/¨ 3.0	4.0(4.0)/ 3.0(3.0)	4.0(4.0)/ 3.0(3.0)
Hydration of i/o head shell*	0.179(0.163)/ 0.231(0.260)	(0.238)/ (0.202)	0.213/ 0.268	0.214(0.285)/ 0.272(0.228)	0.375(0.423)/ 0.404(0.359)
Volume lipid (o/i) [Å^3]	1148(1148)	1156(1150)	1160	1163(1161)	1208(1208)
Fraction of unilamellar vesicles	0.77(0.71)	0.77(0.71)	0.77	0.77(0.71)	0.77(0.71)
Nu	4.0(2.0)	4.0(2.0)	4.0	4.0(2.0)	4.0(2.0)
Number of bilayers	2.5(2.0)	2.5(2.0)	2.5	2.5(2.0)	2.5(2.0)
Sigma_d	3(1.0)	3(10)	3	3(1)	3(1)
Distance between lamella [Å]	59(69)	59(69)	59	59(66)	59(66)
Eta	2.0(0.23)	2.0(0.23)	2.0	2.0(0.23)	2.0(0.23)
Polydispersity (gaussian model)	0.38(0.41)	0.40(0.41)	0.31	0.35(0.36)	0.35(0.36)
SLD head [cm^{-2}]	1.396E+11 (1.396E+11)	1.396E+11 (1.396E+11)	1.396E+11 (1.396E+11)	1.396E+11 (1.396E+11)	1.396E+11 (1.396E+11)
SLD tail [cm^{-2}]	8.347E+10 (8.347E+10)	8.266E+10 (8.326E+10)	8.232E+10	8.201E+10 (8.216E+10)	7.774E+10 (7.776E+10)

The scattering data from the references of the pure surfactant samples were analysed by the different model as described in section S2. SDS was analysed with the ellipsoidal micelle model with a hard-sphere structure factor referred to in section S2.3. Representative fits are shown in Figure S5a) and the corresponding parameters are listed in Table S4. Only the tail densities were varied with the temperature changes to account for the change in measured density. The CMC of 0.7mg/ml (2.4mM) was found to be lower in our Tris solutions than the one usually found for SDS in pure water (2.4mg/ml/8mM), this is quite clear from the SAXS data which still shows able micelle scattering at 1.25 mg/ml.

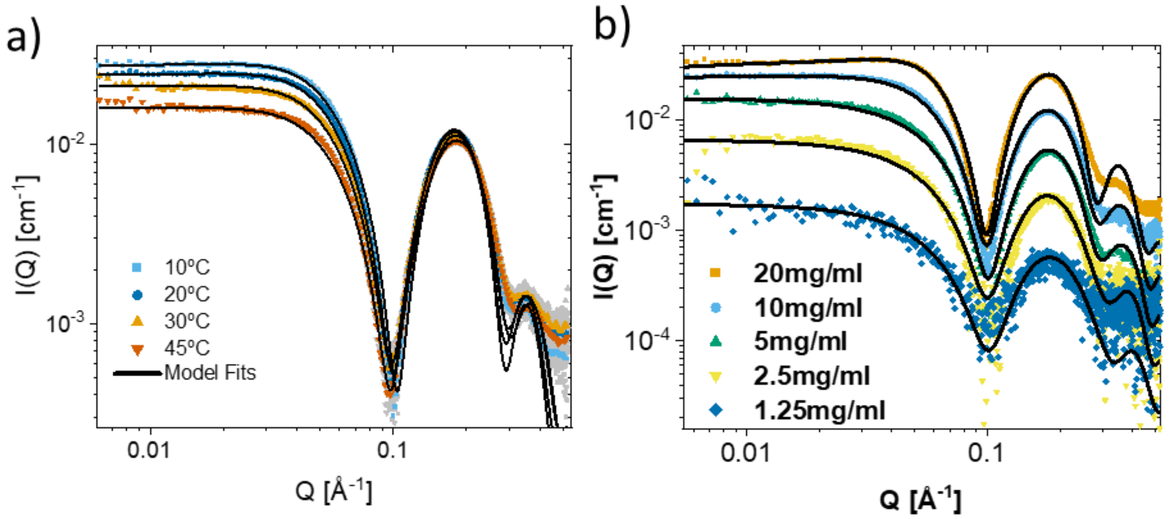

Figure S6: *a) SAXS measurements of SDS 10 mg/ml at different temperatures with model fits.b) SAXS measurements of SDS at 20°C at different concentrations.*

Table S4: *Model fit parameters from analysis of SAXS data SDS samples analysed using the ellipsoidal core-shell model with the hard-sphere structure factor. The SLD of the solvent is 9.43E+10. *calculated values.*

**Model fits of SAXS data from 10mg/ml SDS micelles at different temperatures**				
	10°C	20°C	30°C	45°C
**Spheroidal micelle model**				
Equatorial radius [Å]	1.27	1.32	1.37	1.37
Aspect ratio [Å]	16.1	16.5	16.6	16.7
Thickness headgroup shell [Å]	6.6	5.5	4.2	3.0
Counter-ion fraction	0.95	0.93	0.91	0.9
Smearing [Å]	2.0	2.0	2.0	0.9
Hydration of head shell*	0.096	0.124	0.173	0.266
Hard-sphere radius	58	58	58	58
Z	0.3	0.3	0.3	0.3
Scale of structure factor	4.3	4.4	4.0	3.5
CMC [mg/ml]	0.7	0.7	0.7	0.7
SLD tail group [cm^{-2}]*	7.888E+10	7.772E+10	7.667E+10	7.582E+10
SLD head group [cm^{-2}]*	2.726E+11	2.726E+11	2.726E+11	2.726E+11

DDM was analysed with the triaxial micelle model referred to in section S2.3. Representative fits are shown in Figure S4 and the corresponding parameters are listen in Table S5. Both head and tail densities were varied with the temperature changes, but constricted to the measured total density, resulting in varying SLDs for both groups. The CMC of DDM is very low, usually estimated to be less than 0.08mg/ml (0.2mM) in water. We put the CMC to this value for the fits, but it makes no significant difference to put the CMC as zero.

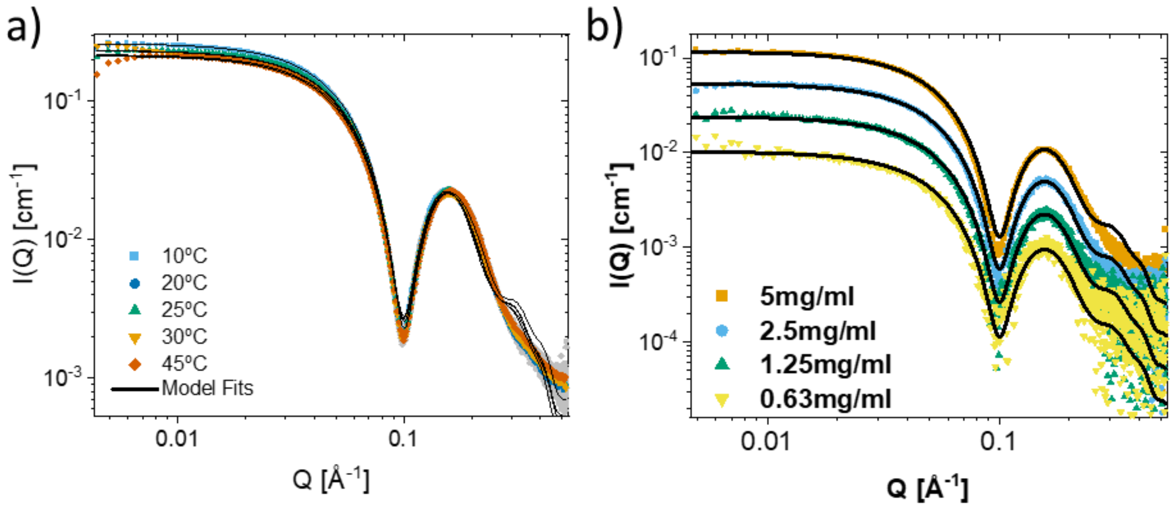

Figure S7: a) *SAXS measurements of DDM 10mg/ml at different temperatures with model fit. b) SAXS measurements of DDM at 20°C at different concentrations.*

Table S5: *Model fit parameters from analysis of SAXS data on DDM samples analysed using the triaxial core-shell model. The SLD of the solvent is 9.43E+10. *calculated values.*

**Model fits of SAXS data from 10mg/ml DDM micelles at different temperatures**					
	10°C	20°C	25°C	30°C	45°C
**Triaxial ellipsoidal micelle model**					
1st radial axis [Å]	30.8	31.1	31.5	30.5	32.3
2nd radial axis [Å]	21.7	21.3	21.2	21.6	21.4
3rd radial axis[Å]	15.7	15.8	15.7	15.9	15.8
Thickness headgroup shell [Å]	8.0	7.2	7.3	6.3	5.6
Smearing [Å]	0.2	0.2	0.2	1.3	0.9
Hydration of head shell*	0.378	0.287	0.309	0.179	0.059
CMC [mg/ml]	0.08	0.08	0.08	0.08	0.08
SLD tail group [cm^{-2}]*	7.891E+10	7.805E+10	7.761E+10	7.661E+10	7.551E+10
SLD head group [cm^{-2}]*	1.480E+11	1.467E+11	1.476E+11	1.480E+11	1.490E+11

TX-100 was analysed with the Triton X-100 micelle model describe in section S2.4. Representative fits are shown in Figure S3a) and the corresponding parameters are listen in Table S6. Only the headgroup density of TX-100 was varied with temperature to account for volume changes but note that the SLD of the shell group also includes the fraction of tails. The CMC was estimated to be 0.3 mg/ml (0.48 mM) from the fits, which is close to albeit a little higher than the value found usually in water. The contrast of the TX-100 micelles becomes very low at low concentrations of surfactants, so this could be an overestimation; however, the scattering at 0.3mg/ml compared with that at lower concentration is indiscernible as seen in Figure S3b), so it should not be much lower.

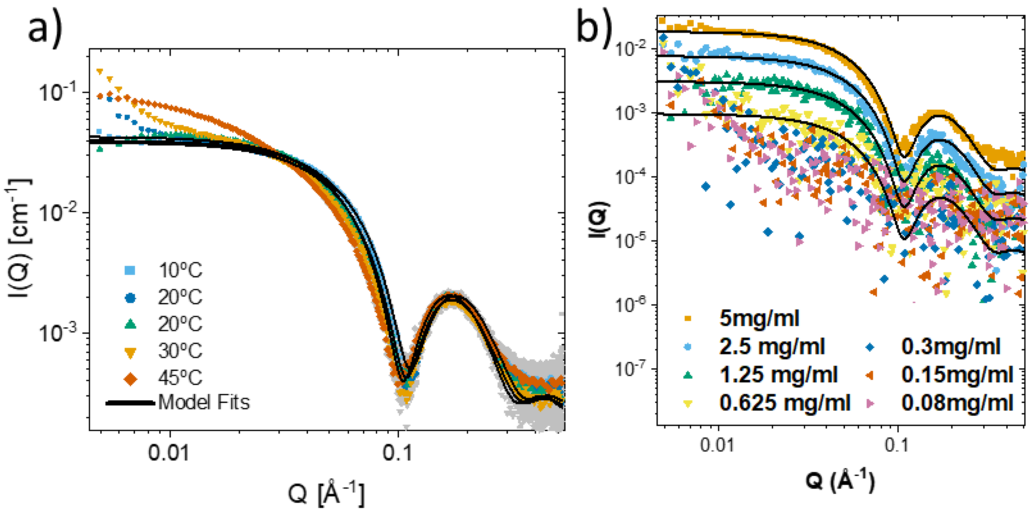

Figure S8: *a) SAXS measurements of TX-100 10mg/ml at different temperatures with model fits. Fits have not been achieved for temperatures above 30°C.* b) *SAXS measurements of TX-100 at 20°C at different concentrations.*

Table S6: *Model fit parameters from analysis of SAXS data on TX-100 samples analysed using the spherical core-shell model with density gradient and blob scattering. The SLD of the solvent is 9.43E+10. *calculated values.*

**Model fits of SAXS data from 10mg/ml TX-100 micelles at different temperatures**			
	10°C	20°C	25°C
**Core-Shell with density profile and blob scattering**			
Aggregation number	56.4	69.8	89.4
Micellar radius [Å]	29.5	30.93	30.2
Density profile sigma	0.2	0.2	0.23
Fraction of TX-100 tails in core	0.41	0.36	0.34
Smearing [Å]	0.9	0.9	0.2
Hydration of head shell*	0.510	0.468	0.266
CMC [mg/ml]	0.3	0.3	0.3
SLD core group [cm^{-2}]*	8.084E+10	8.084E+10	8.084E+10
SLD shell group [cm^{-2}]*	1.073E+11	1.055E+11	1.047E+11

S3.2: DPPC:SDS mixtures

Four different analytical scattering models were used to analyse the scattering data from the DPPC:SDS: a 4-shell vesicle model which accounts for the inserted SDS molecules, a bicelle model, a coexistence between vesicles and bicelle, coexistence between vesicles and ellipsoids and a bicelle model. The scattering data along with the model fits at different ratios and temperatures is shown in Figure S6a). The fraction of lipid in bicelle/ellipsoidal micelle is plotted as a function of SDS concentration in Figure S6b).

Table S7: *Model fit parameters from analysis of the SAXS data from 2.5mg/ml DPPC vesicles mixed with 0.15mg/ml SDS at different temperatures*

**Model fits of SAXS data from 2.5mg/ml DPPC vesicles mixed with 0.15 mg/ml SDS at different temperatures**				
	10°C	20°C	30°C	45°C
Mixed vesicle model				
Average radius vesicle [Å]	225	252	265	310
Thickness of i/o tail shell vesicle [Å]	16.6	16.5	16.0	13.6
Thickness of i/o head shell vesicle [Å]	10.7/7.0	10.7/7.0	10.0/7.0	9.0/8.0
Smearing (i/o tail region) vesicle [Å]	4.8	4.8	4.0	3.6
Smearing (i/o head region) vesicle [Å]	2.0/1.5	2.0/1.5	3.0/2.0	2.0/2.0
Hydration of i/o head shell vesicle*	0.280/0.187	0.291/0.198	0.275/0.218	0.382/0.439
Volume lipid (i/o) vesicle [$Å^3$]	1143	1149	1156	1207
Fraction of SDS in inner leaflet	0.5	0.5	0.5	0.5
Fraction of unilamellar vesicles	0.81	0.81	0.87	0.85
Nu	1.0	1.0	1.0	1.0
Number of bilayers	2.0	2.0	2.0	2.0
Sigma_d	1.0	1.0	1.0	1.0
Distance between lamella [Å]	65	65	66	77
Eta	0.2	0.2	0.2	0.2
Polydispersity vesicle (gaussian model)	0.38	0.38	0.38	0.38
SLD average head [cm^{-2}]	1.419E+11	1.419E+11	1.419E+11	1.419E+11
SLD average tail [cm^{-2}]	8.367E+10	8.302E+10	8.229E+10	7.773E+10

Table S8: *Model fit parameters from analysis of the SAXS data from 2.5mg/ml DPPC vesicles mixed with 0.31mg/ml SDS at different temperatures*

**Model fits of SAXS data from 2.5mg/ml DPPC vesicles mixed with 0.31 mg/ml SDS at different temperatures**				
	10°C	20°C	30°C	45°C
Mixed vesicle model				
Average radius vesicle [Å]	252	252	255	310
Thickness of i/o tail shell vesicle [Å]	16.2	16.2	16.0	13.6

Thickness of i/o head shell vesicle [Å]	10.7/7.0	10.7/7.0	10.0/7.0	9.0/8.0
Smearing (i/o tail region) vesicle [Å]	4.9	4.9	4.0	3.6
Smearing (i/o head region) vesicle [Å]	2.0/1.5	2.0/1.5	3.0/2.0	2.0/2.0
Hydration of i/o head shell vesicle*	0.325/0.234	0.239/0.238	0.301/0.253	0.382/0.439
Volume lipid (i/o) vesicle [Å^3]	1145	1149	1156	1207
Fraction of SDS in inner leaflet	0.5	0.5	0.5	0.5
Fraction of unilamellar vesicles	0.75	0.84	0.97	0.95
Nu	1.0	1.0	1.0	1.0
Number of bilayers	2.0	2.0	2.0	2.0
Sigma_d	1.0	1.0	1.0	1.0
Distance between lamella [Å]	65	65	66	92
Eta	0.3	0.2	0.2	0.2
Polydispersity vesicle (gaussian model)	0.38	0.38	0.38	0.38
SLD average head [cm^{-2}]	1.442E+11	1.442E+11	1.442E+11	1.442E+11
SLD average tail [cm^{-2}]	8.320E+10	8.270E+10	8.194E+10	7.778E+10

Table S9: *Model fit parameters from analysis of the SAXS data from 2.5mg/ml DPPC vesicles mixed with 0.63mg/ml SDS at different temperatures*

**Model fits of SAXS data from 2.5mg/ml DPPC vesicles mixed with 0.63 mg/ml SDS at different temperatures**				
	10°C	20°C	30°C	45°C
**Mixed vesicle model**				
Average radius vesicle [Å]	246	246	266	298
Thickness of i/o tail shell vesicle [Å]	15.1	14.9	15.7	12.8
Thickness of i/o head shell vesicle [Å]	10.9/6.9	10.9/6.9	10.0/7.0	9.0/8.0
Smearing (i/o tail region) vesicle [Å]	5	5	4.8	3.1
Smearing (i/o head region) vesicle [Å]	3.0/2.0	3.0/2.0	1.0/1.0	2.0/2.0
Hydration of i/o head shell vesicle*	0.428/ 0.323	0.441/ 0.337	0.636/ 0.309	0.462/ 0.511
Volume lipid (i/o) vesicle [Å^3]	1157	1163	1161	1198
Fraction of SDS in inner leaflet	0.5	0.5	0.5	0.5
Fraction of unilamellar vesicles	0.83	0.86	0.95	1.0
Nu	0	0	1	NA
Number of bilayers	2.0	2.0	2.0	NA
Sigma_d	1.0	1.0	1.0	NA
Distance between lamella [Å]	65	65	69	NA
Eta	0.3	0.3	0.2	NA
Polydispersity vesicle (gaussian model)	0.40	0.40	0.34	0.34

SLD average head [cm^{-2}]	1.534E+11	1.534E+11	1.484E+11	1.479E+11
SLD average tail [cm^{-2}]	8.178E+10	8.106E+10	8.099E+10	7.806E+10

Table S10: *Model fit parameters from analysis of the SAXS data from 2.5mg/ml DPPC vesicles mixed with 1.25mg/ml SDS at different temperatures*

**Model fits of SAXS data from 2.5mg/ml DPPC vesicles mixed with 1.25 mg/ml SDS at different temperatures**					
	10°C	20°C	30°C		45°C
**Mixed vesicle +ellipsoid model**			**Mixed vesicle +bicelle model**		
Average radius vesicle [Å]	250	258	286		320
Thickness of i/o tail shell vesicle [Å]	15.1	15.1	14.6		11.5
Thickness of i/o head shell vesicle [Å]	10.0/8.0	10.0/8.0	9.2/7.9		8.8/8.1
Smearing (i/o tail region) vesicle [Å]	5	5	4.8		3.4
Smearing (i/o head region) vesicle [Å]	3.0/2.0	3.0/2.5	3.0/2.0		3.0/2.0
Hydration of i/o head shell vesicle*	0.374/ 0.411	0.383/ 0.416	0.384/ 0.439		0.569/ 0.610
Volume lipid (i/o) vesicle [$Å^3$]	1154	1160	1182		1221
Fraction of SDS in inner leaflet	0.5	0.5	0.5		0.5
Fraction of unilamellar vesicles	0.85	0.88	1		1.0
Nu	0	0	NA		NA
Number of bilayers	2.0	2.0	NA		NA
Sigma_d	1.0	1.0	NA		NA
Distance between lamella [Å]	65	65	NA		NA
Eta	0.4	0.4	NA		NA
Polydispersity vesicle (gaussian model)	0.37	0.35	0.37		0.47
SLD average head vesicle [cm^{-2}]	1.527E+11	1.529E+11	1.534E+11		
SLD average tail vesicle [cm^{-2}]	8.207E+10	8.133E+10	7.938E+10		
Fraction lipid/SDS in micelles	0.02/0.54	0.08/0.56	Fraction lipid/SDS in bicelles	0.19/ 0.58	0/0
Equatorial radius [Å]	18.2	18.2	Radius bicelle [Å]	31.3	NA
Aspect ratio	1.27	1.27	Thickness of bicelle tail region disc [Å]	26.9	NA
Thickness headgroup shell [Å]	7.0	7.0	Thickness of bicelle head shell [Å]	19.0	NA
Counter-ion fraction	0.91	0.91	Thickness of bicelle rim [Å]	10.0	NA
Volume lipid in micelle [$Å^3$]	1056	1056	Volume lipid in bicelle [$Å^3$]	1171	NA

Smearing	2.5	2.5	Hydration of head shell/rim*	0.760/ 0.973	
Hydration of head shell*	0. 113	0. 132	Fraction of lipid/DDM in rim	0.01/ 0.70	NA
Hard-sphere radius [Å]	54	54	SLD average head surface bicelle[cm^{-2}]	1.627E+11	NA
Z	0.5	0.5	SLD average head rim[cm^{-2}]	2.676E+11	NA
Scale of structure factor	5	5	SLD average tail bicelle[cm^{-2}]	7.837E+10	NA

Table S11: *Model fit parameters from analysis of the SAXS data from 2.5mg/ml DPPC vesicles mixed with 2.5mg/ml SDS at different temperatures*

**Model fits of SAXS data from 2.5mg/ml DPPC vesicles mixed with 2.5 mg/ml SDS at different temperatures**			
	10°C	20°C	30°C
**Mixed vesicle +ellipsoid model**			**Mixed vesicle +bicelle model**
Average radius vesicle [Å]	250	251	221
Thickness of i/o tail shell vesicle [Å]	15.6	15.2	14.0
Thickness of i/o head shell vesicle [Å]	10.0/8.0	10.0/7.0	8.6/7.5
Smearing (i/o tail region) vesicle [Å]	5	5	1.6
Smearing (i/o head region) vesicle [Å]	3.0/2.5	3.0/2.2	3.2/2.5
Hydration of i/o head shell vesicle*	0.339/ 0.383	0.379/ 0.341	0.443/0.523
Volume lipid (i/o) vesicle [Å^3]	1148	1163	1185
Fraction of SDS in inner leaflet	0.5	0.5	0.5
Fraction of unilamellar vesicles	0.85	0.92	1
Nu	0	0	NA
Number of bilayers	2.0	2.0	NA
Sigma_d	1.0	1.0	NA
Distance between lamella [Å]	65	65	NA
Eta	0.4	0.4	NA
Polydispersity vesicle (gaussian model)	0.37	0.37	0.37
SLD average head vesicle [cm^{-2}]	1.514E+11	1.532E+11	1.728E+11
SLD average tail vesicle [cm^{-2}]	8.262E+10	8.108E+10	7.848E+10

Fraction lipid/SDS in micelles	0.17/0.83	0.14/0.79	Fraction lipid/SDS in bicelles	0.86/0.90
Equatorial radius [Å]	17.4	17.6	Radius bicelle [Å]	30.1
Aspect ratio	1.27	1.27	Thickness of bicelle tail region disc [Å]	26.0
Thickness headgroup shell [Å]	8.0	6.5	Thickness of bicelle head shell [Å]	22.0
Counter-ion fraction	0.91	0.93	Thickness of bicelle rim [Å]	10.1
Volume lipid in micelle [$Å^3$]	1056	1056	Volume lipid in bicelle [$Å^3$]	1156
Smearing	2.5	2.5	Hydration of head shell/rim*	0.743/ 0.979
Hydration of head shell*	0. 100	0. 132	Fraction of lipid/DDM in rim	0.13/0.58
Hard-sphere radius [Å]	54	54	SLD average head surface bicelle[cm^{-2}]	1.644E+11
Z	0.5	0.5	SLD average head rim[cm^{-2}]	2.290E+11
Scale of structure factor	5	5	SLD average tail bicelle[cm^{-2}]	7.944E+10

Table S12*: Model fit parameters from analysis of the SAXS data from 2.5mg/ml DPPC vesicles mixed with 5mg/ml SDS at different temperatures*

**Model fits of SAXS data from 2.5mg/ml DPPC vesicles mixed with 5.0 mg/ml SDS at different temperatures**				
	10°C	20°C	30°C	45°C
**Mixed vesicle +ellipsoid model**			**Mixed vesicle +bicelle model**	
Average radius vesicle [Å]	239	242	250	400
Thickness of i/o tail shell vesicle [Å]	15.1	15.0	15.0	15.0
Thickness of i/o head shell vesicle [Å]	10.0/7.1	10.0/7.1	8.6/7.5	8.7/7.5
Smearing (i/o tail region) vesicle [Å]	5	5	1.6	1.6
Smearing (i/o head region) vesicle [Å]	3.0/2.2	3.0/2.2	3.2/2.5	3.2/2.5
Hydration of i/o head shell vesicle*	0.380/ 0.349	0.395/ 0.362	0.479/ 0.546	0.447/ 0.468
Volume lipid (i/o) vesicle [$Å^3$]	1154	1163	1135	1135
Fraction of SDS in inner leaflet	0.5	0.5	0.5	0.5
Fraction of unilamellar vesicles	NA	0.92	1	1.0
Nu	NA	0	NA	NA
Number of bilayers	NA	2.0	NA	NA
Sigma_d	NA	1	NA	NA
Distance between lamella [Å]	NA	65	NA	NA
Eta	NA	0.4	NA	NA
Polydispersity vesicle (gaussian model)	0.37	0.37	0.40	0.40

SLD average head vesicle [cm^{-2}]	1.546E+11	1.551E+11	1.94355244E+11		1.551E+11
SLD average tail vesicle [cm^{-2}]	8.196E+10	8.097E+10	7.96968984E+10		8.097E+10
Fraction lipid/SDS in micelles	0.12/0.88	0.22/0.89	Fraction lipid/SDS in bicelles	0.996/ 0.997	0.998/ 0.999
Equatorial radius [Å]	17.2	17.6	Radius bicelle [Å]	23.8	23.3
Aspect ratio	1.27	1.27	Thickness of bicelle tail region disc [Å]	26.2	25.2
Thickness headgroup shell [Å]	7.3	6.1	Thickness of bicelle head shell [Å]	8.7	8.7
Counter-ion fraction	0.91	0.91	Thickness of bicelle rim [Å]	14.3	14.3
Volume lipid in micelle [Å^3]	1135	1125	Volume lipid in bicelle [Å^3]	1155	1150
Smearing	2.5	2.5	Hydration of head shell/rim*	0.760/ 0.693	0.768/ 0.969
Hydration of head shell*	0. 099	0. 134	Fraction of lipid/SDS in rim	0.08/0.62	0.08/0.62
Hard-sphere radius [Å]	65	60	SLD average head surface bicelle[cm^{-2}]	1.758E+11	1.759E+11
Z	0.5	2.7	SLD average head rim bicelle [cm^{-2}]	2.543E+11	2.543E+11
Scale of structure factor	28	20	SLD average tail bicelle[cm^{-2}]	7.858E+10	7.812E+10

Table S13: *Model fit parameters from analysis of the SAXS data from 2.5mg/ml DPPC vesicles mixed with 10mg/ml SDS at different temperatures*

**Model fits of SAXS data from 2.5mg/ml DPPC vesicles mixed with 10.0 mg/ml SDS at different temperatures**				
	10°C	20°C	30°C	45°C
**Mixed vesicle +ellipsoid model**			**Bicelle model**	
Average radius vesicle [Å]	231	255	NA	NA
Thickness of i/o tail shell vesicle [Å]	15.1	15.1	NA	NA
Thickness of i/o head shell vesicle [Å]	10.0/7.1	10.0/7.1	NA	NA
Smearing (i/o tail region) vesicle [Å]	5	5	NA	NA

Smearing (i/o head region) vesicle [Å]	3.0/2.2	3.0/2.2	NA		NA
Hydration of i/o head shell vesicle*	0.408/0.385	0.423/0.385	NA		NA
Volume lipid (i/o) vesicle [Å^3]	1154	1163	NA		NA
Fraction of SDS in inner leaflet	0.5	0.5	NA		NA
Polydispersity vesicle (gaussian model)	0.37	0.31	NA		NA
Fraction lipid/SDS in micelles	0.41/0.94	0.41/0.94	Fraction lipid/SDS in bicelles	1/1	1/1
Equatorial radius [Å]	21.2	20.7	Radius bicelle [Å]	20.25	20.4
Aspect ratio	0.71	0.75	Thickness of bicelle tail region disc [Å]	26.6	26.9
Thickness headgroup shell [Å]	7.9	6.5	Thickness of bicelle head shell [Å]	6.9	5.7
Counter-ion fraction	0.91	0.91	Thickness of bicelle rim [Å]	10.9	10.5
Volume lipid in micelle [Å^3]	1162	1168	Volume lipid in bicelle [Å^3]	1139	1141
Smearing	2.3	2.0	Hydration of head shell/rim*	0.758/0.957	0.702/0.952
Hydration of head shell*	0. 122	0. 151	Fraction of lipid/SDS in rim	0.08/0.58	0.08/0.58
SLD average head vesicle [cm^{-2}]	1.608E+11	1.608E+11	SLD average head surface bicelle[cm^{-2}]	1.990E+11	1.992E+11
SLD average tail vesicle [cm^{-2}]	8.165E+10	8.067E+10	SLD average head rim bicelle [cm^{-2}]	2.607E+11	2.612E+11
Hard-sphere radius [Å]	55	55	SLD average tail bicelle[cm^{-2}]	7.808E+10	7.733E+10
Z	2.7	2.7			
Scale of structure factor	10	8			

Table S14: *Model fit parameters from analysis of the SAXS data from 2.5mg/ml DPPC vesicles mixed with 0.63 mg/ml SDS at different temperatures*

**Model fits of SAXS data from 2.5mg/ml DPPC vesicles mixed with 20.0 mg/ml SDS at different temperatures**				
	10°C	20°C	30°C	45°C
**Mixed vesicle +ellipsoid model**			**Ellipsoidal model**	
Average radius vesicle [Å]	235	259	NA	NA
Thickness of i/o tail shell vesicle [Å]	15.1	14.6	NA	NA
Thickness of i/o head shell vesicle [Å]	10.0/7.1	10.0/7.1	NA	NA
Smearing (i/o tail region) vesicle [Å]	5	5	NA	NA
Smearing (i/o head region) vesicle [Å]	3.0/2.2	3.0/2.2	NA	NA
Hydration of i/o head shell vesicle*	0.457/0.433	0.489/0.450	NA	NA
Volume lipid (i/o) vesicle [Å^3]	1165	1171	NA	NA
Fraction of SDS in inner leaflet	0.5	0.5	NA	NA
Polydispersity vesicle (gaussian model)	0.34	0.29	NA	NA
Fraction lipid/SDS in micelles	0.63/0.97	0.63/0.97	1/1	1/1
Equatorial radius [Å]	16.4	16.4	21.0	21.0
Aspect ratio	1.33	1.38	0.73	0.78
Thickness headgroup shell [Å]	7.1	6.3	6.7	7.1
Counter-ion fraction	0.91	0.91	0.91	0.91
Volume lipid in micelle [Å^3]	1164	1164	1139	1185
Smearing	1.6	2.0	2.5	2.5
Hydration of head shell*	0. 097	0. 113	0.145	0.137
Hard-sphere radius [Å]	51	51	50	51
Z	0.5	0.5	0.3	0.2
Scale of structure factor	6.6	6.3	5.6	5.6
SLD average head [cm^{-2}]	1.705E+11	1.705E+11	2.425E+11	2.425E+11
SLD average tail [cm^{-2}]	8.059E+10	7.977E+10	7.629E+10	7.586E+10

S3.3: DMPC:SDS mixtures

Four different analytical scattering models were used to analyse the scattering data from the DMPC:SDS: a 4-shell vesicle model which accounts for the inserted SDS molecules, a bicelle model, a rod model and an ellipsoidal micelle model. The scattering data along with the model fits at different ratios and temperatures is shown in Figure S7a). Inverse Fourier transform analysis was also used to give an indication of which structures were present at the 1:1 ratio shown in Figure S8b). The variation of the vesicle radius found from the fits is shown in Figure S8c) and the variation in the lamellar repeat spacing is shown in Figure S8d).

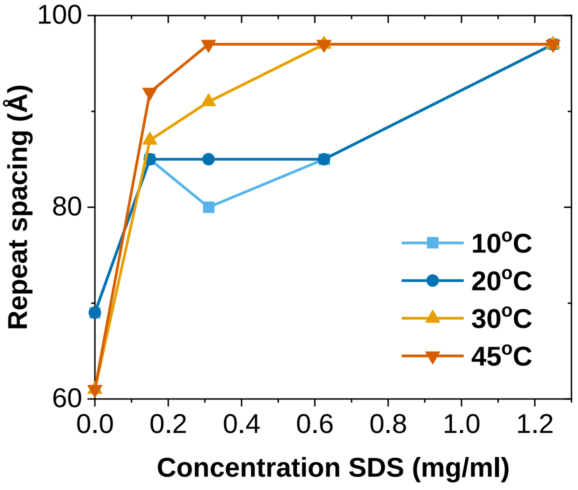

Figure S9: *The repeat spacing between lamella as a function of the concentration of SDS for different temperatures.*

Table S15: *Model fit parameters from analysis of the SAXS data from 2.5mg/ml DMPC vesicles mixed with 0.15mg/ml SDS at different temperatures*

Model fits of SAXS data from 2.5mg/ml DMPC vesicles mixed with 0.15 mg/ml SDS at different temperatures				
	10°C	20°C	30°C	45°C
Mixed vesicle model				
Average radius vesicle [Å]	227	271	311	276
Thickness of i/o tail shell vesicle [Å]	14.6	13.8	11.7	11.1
Thickness of i/o head shell vesicle [Å]	11.0/6.5	11.0/7.5	9.0/8.2	9.0/8.2
Smearing (i/o tail region) vesicle [Å]	4.6	4.4	3.7	3.7
Smearing (i/o head region) vesicle [Å]	1.5/1.0	2.5/2.0	1.5/1.0	1.5/1.0
Hydration of i/o head shell vesicle*	0.309/0.136	0.376/0.288	0.429/0.483	0.457/0.516
Volume lipid (i/o) vesicle [Å^3]	1052	1063	1104	1110
Fraction of SDS in inner leaflet	0.5	0.5	0.5	0.5
Fraction of unilamellar vesicles	0.85	0.85	0.85	0.85
Nu	1.0	1.0	1.0	1.0
Number of bilayers	2.0	2.0	2.0	2.0
Sigma_d	1.0	1.0	1.0	1.0
Distance between lamella [Å]	85	85	87	92
Eta	0.3	0.3	0.15	0.15
Polydispersity vesicle (gaussian model)	0.29	0.32	0.26	0.28
SLD average head [cm^{-2}]	1.429E+11	1.429E+11	1.463E+11	1.461E+11
SLD average tail [cm^{-2}]	8.187E+10	8.064E+10	7.659E+10	7.595E+10

Table S16: *Model fit parameters from analysis of the SAXS data from 2.5mg/ml DMPC vesicles mixed with 0.31mg/ml SDS at different temperatures*

Model fits of SAXS data from 2.5mg/ml DMPC vesicles mixed with 0.31 mg/ml SDS at different temperatures				
	10°C	20°C	30°C	45°C
Mixed vesicle model				
Average radius vesicle [Å]	215	232	323	292

Thickness of i/o tail shell vesicle [Å]	14.2	14.0	12.1	11.2
Thickness of i/o head shell vesicle [Å]	11.0/6.5	12.0/5.5	9.0/8.0	9.0/8.0
Smearing (i/o tail region) vesicle [Å]	4.7	4.8	4.1	3.5
Smearing (i/o head region) vesicle [Å]	1.5/1.0	1.5/1.0	1.5/1.0	1.5/1.0
Hydration of i/o head shell vesicle*	0.356/0.200	0.432/0.071	0.407/0.448	0.452/0.494
Volume lipid (i/o) vesicle [Å^3]	1056	1072	1099	1105
Fraction of SDS in inner leaflet	0.5	0.5	0.5	0.5
Fraction of unilamellar vesicles	0.85	0.90	0.87	0.85
Nu	1.0	1.0	1.0	1.0
Number of bilayers	2.0	2.0	2.0	2.0
Sigma_d	1.0	1.0	1.0	1.0
Distance between lamella [Å]	80	85	91	97
Eta	0.3	0.5	0.3	0.2
Polydispersity vesicle (gaussian model)	0.42	0.42	0.29	0.28
SLD average head [cm^{-2}]	1.464E+11	1.463E+11	1.463E+11	1.461E+11
SLD average tail [cm^{-2}]	8.129E+10	7.961E+10	7.701E+10	7.638E+10

Table S17: *Model fit parameters from analysis of the SAXS data from 2.5mg/ml DMPC vesicles mixed with 0.63mg/ml SDS at different temperatures*

**Model fits of SAXS data from 2.5mg/ml DMPC vesicles mixed with 0.63 mg/ml SDS at different temperatures**				
	10°C	20°C	30°C	45°C
Mixed vesicle model				
Average radius vesicle [Å]	223	228	311	301
Thickness of i/o tail shell vesicle [Å]	13.9	12.8	11.7	11.2
Thickness of i/o head shell vesicle [Å]	11.5/5.5	9.2/7.9	8.9/7.9	8.5/8.3
Smearing (i/o tail region) vesicle [Å]	4.4	4.2	4.3	4.3
Smearing (i/o head region) vesicle [Å]	3.8/1.2	2.1/1.8	2.1/1.8	2.1/1.8
Hydration of i/o head shell vesicle*	0.437/0.123	0.390/0.460	0.457/0.495	0.458/0.542
Volume lipid (i/o) vesicle [Å^3]	1056	1090	1101	1106
Fraction of SDS in inner leaflet	0.5	0.5	0.5	0.5
Fraction of unilamellar vesicles	0.85	0.90	0.85	0.85
Nu	1.0	1.0	1.0	1.0
Number of bilayers	2.0	2.0	2.0	2.0
Sigma_d	1.0	1.0	1.0	1.0
Distance between lamella [Å]	85	85	97	97
Eta	0.3	0.3	0.5	0.5
Polydispersity vesicle (gaussian model)	0.47	0.49	0.33	0.32
SLD average head [cm^{-2}]	1.525E+11	1.525E+11	1.525E+11	1.520E+11
SLD average tail [cm^{-2}]	8.102E+10	7.788E+10	7.681E+10	7.624E+10

Table S18: *Model fit parameters from analysis of the SAXS data from 2.5mg/ml DMPC vesicles mixed with 1.25mg/ml SDS at different temperatures*

**Model fits of SAXS data from 2.5mg/ml DMPC vesicles mixed with 1.25 mg/ml SDS at different temperatures**				
	10°C	20°C	30°C	45°C
Mixed vesicle model				
Average radius vesicle [Å]	350	379	379	311

Thickness of i/o tail shell vesicle [Å]	12.6	12.3	11.5	10.9
Thickness of i/o head shell vesicle [Å]	11.8/7.4	9.9/8.4	9.3/7.7	8.5/8.3
Smearing (i/o tail region) vesicle [Å]	5.2	5.0	4.4	4.4
Smearing (i/o head region) vesicle [Å]	3.0/2.7	2.5/2.0	2.5/2.0	2.1/1.8
Hydration of i/o head shell vesicle*	0.583/0.448	0.539/0.541	0.548/0.534	0.527/0.596
Volume lipid (i/o) vesicle [$Å^3$]	1062	1098	1103	1108
Fraction of SDS in inner leaflet	0.5	0.5	0.5	0.5
Polydispersity vesicle (gaussian model)	0.34	0.39	0.30	0.36
SLD average head [cm^{-2}]	1.633E+11	1.630E+11	1.630E+11	1.621E+11
SLD average tail [cm^{-2}]	8.021E+10	7.735E+10	7.666E+10	7.604E+10

Table S19: *Model fit parameters from analysis of the SAXS data from 2.5mg/ml DMPC vesicles mixed with 2.5mg/ml SDS at different temperatures*

**Model fits of SAXS data from 2.5mg/ml DMPC vesicles mixed with 2.5 mg/ml SDS at different temperatures**				
	10°C	20°C	30°C	45°C
Rod model				
Radius tail region rod [Å]	15.6	15.0	14.3	13.0
Length of rod [Å]	450	510	420	404
Thickness of rod head shell [Å]	14.7	15.8	16.4	17.6
Thickness of rod cap [Å]	6.4	6.8	6.3	6.3
Volume lipid in rod [$Å^3$]	1037	1041	1055	1069
Hydration of head shell*	0.893	0.908	0.919	0.936
Interface smearing	3.1	3.0	3.4	3.1
SLD average head [cm^{-2}]	1.797E+11	1.797E+11	1.797E+11	1.797E+11
SLD average tail [cm^{-2}]	8.053E+10	8.030E+10	7.959E+10	7.888E+10

Table S20: *Model fit parameters from analysis of the SAXS data from 2.5mg/ml DMPC vesicles mixed with 5mg/ml SDS at different temperatures*

**Model fits of SAXS data from 2.5mg/ml DMPC vesicles mixed with 5.0 mg/ml SDS at different temperatures**				
	10°C	20°C	30°C	45°C
Bicelle model				
Radius bicelle [Å]	28.4	27.0	24.6	23.4
Thickness of bicelle tail region disc [Å]	22.5	21.9	22.5	22.1
Thickness of bicelle head shell [Å]	10.9	10.9	10.2	10.1
Thickness of bicelle rim [Å]	12.9	12.4	15.1	17.8
Volume lipid in bicelle [$Å^3$]	1022	1041	1052	1070
Hydration of head shell/sim*	0.795/0.972	0.807/0.972	0.792/0.978	0.796/0.983
Fraction of lipid/TX-100 in rim	0.030/0.48	0.03/0.51	0.03/0.54	0.03/0.55
Interface smearing	2.0	2.0	2.0	2.0
SLD average head [cm^{-2}]	2.669E+11	2.655E+11	2.635E+11	2.677E+11
SLD average tail [cm^{-2}]	7.992E+10	7.935E+10	7.871E+10	7.821E+10

Table S21: *Model fit parameters from analysis of the SAXS data from 2.5mg/ml DMPC vesicles mixed with 10mg/ml SDS at different temperatures*

**Model fits of SAXS data from 2.5mg/ml DMPC vesicles mixed with 10.0 mg/ml SDS at different temperatures**				
	10°C	20°C	30°C	45°C
Radius bicelle [Å]	22.1	21.2	20.8	20.2
Thickness of bicelle tail region disc [Å]	26.5	24.4	24.5	25.1
Thickness of bicelle head shell [Å]	5.0	7.3	6.3	5.0
Thickness of bicelle rim [Å]	9.5	9.9	10.3	10.1
Volume lipid in bicelle [$Å^3$]	1092	1052	1045	1047
Hydration of head shell/sim*	0.631/0.943	0.764/0.956	0.737/0.954	0.796/0.983
Fraction of lipid/TX-100 in rim	0.030/0.57	0.03/0.57	0.03/0.59	0.03/0.55
Interface smearing	2.0	2.0	2.0	2.0
SLD average head [cm^{-2}]	2.710E+11	2.670E+11	2.702E+11	2.617E+11
SLD average tail [cm^{-2}]	7.857E+10	7.850E+10	7.776E+10	7.702E+10

S3.4: DPPC:DDM mixtures

Two different analytical scattering models were used to analyse the scattering data from the DPPC:DDM: a 4-shell vesicle model which accounts for the inserted DDM molecules in coexistence with bicelles and a rod/worm-like micelle model. The scattering data along with the model fits at different ratios and temperatures is shown in Figure S8a). The amounts of DDM and lipid in bicelles/rods plotted versus the concentration of DDM is shown in Figure S8b). Inverse Fourier transform analysis was also used to give an indication of which structures were present at the higher ratios at 45°C, shown in Figure S8c). Tables S22 to S27 show the resultant fit parameters.

Table S22: *Model fit parameters from analysis of the SAXS data from 2.5mg/ml DPPC vesicles mixed with 0.61mg/ml DDM at different temperatures*

**Model fits of SAXS data from 2.5mg/ml DPPC vesicles mixed with 0.61 mg/ml DDM at different temperatures**					
	10°C	20°C	25°C	30°C	45°C
**Vesicle + bicelle model**					
Average radius vesicle [Å]	295	285	306	321	335
Thickness of i/o tail shell vesicle [Å]	16.0/15.3	16.0/15.3	15.9/15.1	15.8/14.7	13.4/12.1
Thickness of i/o head shell vesicle [Å]	9.0/9.6	9.0/9.6	9.0/9.6	9.5/10.0	9.0/7.5
Smearing (i/o tail region) vesicle [Å]	5.0/5.0	5.0/5.0	5.0/5.0	4.6/4.7	4.8/4.8
Smearing (i/o head region) vesicle [Å]	3.0/2.0	3.0/2.0	3.0/2.0	3.0/2.0	3.0/3.0
Hydration of i/o head shell vesicle*	0.273/ 0.106	0.257/ 0.204	0.259/ 0.171	0.305/ 0.205	0.399/ 0.132
Volume lipid (o/i) vesicle [$Å^3$]	1155/ 1156	1158/ 1172	1162/ 1182	1170/ 1183	1235/ 1217
Fraction of DDM in inner leaflet	0.03	0.04	0.1	0.13	0.2

Polydispersity vesicle (gaussian model)	0.36	0.38	0.33	0.25	0.44
Fraction of unilamellar vesicles	0.9	0.9	0.9	0.9	0.5
Nu	0	0	0	0.5	0
Number of bilayers	1.5	1.5	1.5	1.0	3.7
Sigma_d	3.0	3.0	3.0	3.0	1.0
Distance between lamella [Å]	61	61	61	61	62
Eta	0.1	0.1	0.1	0.1	0.1
Fraction lipid/DDM in bicelles	0.30/0.48	0.20/0.57	0.25/0.48	0.28/0.45	0.15/0.25
Radius bicelle [Å]	36.4	40.5	40.5	46.0	58.0
Thickness of bicelle tail region disc [Å]	24.5	24.2	24.2	25.0	20.0
Thickness of bicelle head shell [Å]	6.0	6.0	6.0	9.0	9.0
Thickness of bicelle rim [Å]	9.0	9.0	9.0	9.0	9.0
Volume lipid in bicelle [$Å^3$]	1165	1168	1172	1161	1205
Hydration of head shell/sim*	0.846/ 0.364	0.733/ 0.324	0.792/ 0.369	0.786/ 0.434	0.756/ 0.563
Fraction of lipid/DDM in rim	0.1/0.25	0.05/0.35	0.05/0.35	0.10/0.50	0.10/0.50
Interface smearing	3.6	4.0	4.0	4.0	4.0
SLD average vesicle head i/o [cm^{-2}]	1.397E+11/ 1.418E+11	1.397E+11/ 1.414E+11	1.400E+11/ 1.416E+11	1.401E+11/ 1.417E+11	1.404E+11/ 1.418E+11
SLD average vesicle tail i/o [cm^{-2}]	8.269E+10/ 8.099E+10	8.237E+10/ 8.006E+10	8.191E+10/ 7.913E+10	8.105E+10/ 7.900E+10	7.538E+10/ 7.648E+10
SLD average head surface bicelle [cm^{-2}]	1.417E+11	1.421E+11	1.416E+11	1.412E+11	1.412E+11
SLD average head rim bicelle [cm^{-2}]	1.431E+11	1.443E+11	1.441E+11	1.437E+11	1.437E+11
SLD average tail bicelle [cm^{-2}]	8.017E+10	7.912E+10	7.942E+10	8.046E+10	7.724E+10

Table S23: *Model fit parameters from analysis of the SAXS data from 2.5mg/ml DPPC vesicles mixed with 1.25mg/ml DDM at different temperatures*

**Model fits of SAXS data from 2.5mg/ml DPPC vesicles mixed with 1.25 mg/ml DDM at different temperatures**					
	10°C	20°C	25°C	30°C	45°C
**Vesicle + bicelle model**					
Average radius vesicle [Å]	301	290	300	340	310
Thickness of i/o tail shell vesicle [Å]	16.2/15.4	16.0/15.3	16.0/15.0	15.3/14.2	13.4/12.1
Thickness of i/o head shell vesicle [Å]	9.0/9.6	9.0/9.6	9.0/9.6	9.6/10.0	9.0/8.0
Smearing (i/o tail region) vesicle [Å]	5.0/5.0	5.0/5.0	5.0/5.0	4.4/4.4	4.8/4.8

Smearing (i/o head region) vesicle [Å]	3.0/2.0	3.0/2.0	3.0/2.0	3.0/2.0	3.0/2.0
Hydration of i/o head shell vesicle*	0.243/ 0.208	0.257/ 0.209	0.269/ 0.215	0.320/ 0.305	0.321/ 0.034
Volume lipid (o/i) vesicle [Å^3]	1149/ 1165	1157/ 1172	1159/ 1175	1171/ 1183	1233/ 1224
Fraction of DDM in inner leaflet	0.04	0.04	0.04	0.17	0.27
Polydispersity vesicle (gaussian model)	0.34	0.37	0.29	0.23	0.49
Fraction of unilamellar vesicles	0.9	0.9	0.9	0.9	0.9
Nu	0.0	0.0	0.0	0.5	0.0
Number of bilayers	1.5	1.5	1.5	1.5	3.7
Sigma_d	3.0	3.0	3.0	3.0	1.0
Distance between lamella [Å]	61	61	71	60	62
Eta	0.1	0.1	0.1	0.1	0.1
Fraction lipid/DDM in bicelles	0.19/0.80	0.19/0.78	0.19/0.78	0.19/0.77	0.35/0.43
Radius bicelle [Å]	31.9	32.9	31.4	42.2	42.7
Thickness of bicelle tail region disc [Å]	23.4	22.9	22.6	22.4	20.1
Thickness of bicelle head shell [Å]	6.0	6.0	6.0	9.0	9.0
Thickness of bicelle rim [Å]	9.5	9.0	9.0	9.0	9.0
Volume lipid in bicelle [Å^3]	1189	1213	1219	1233	1248
Hydration of head shell/sim*	0.667/ 0.281	0.722/ 0.210	0.478/ 0.469	0.691/ 0.322	0.758/ 0.586
Fraction of lipid/DDM in rim	0.13/0.34	0.13/0.27	0.13/0.57	0.19/0.36	0.20/0.50
Interface smearing	4.0	4.0	3.2	4.0	4.0
SLD average vesicle head i/o [cm^{-2}]	1.397E+11/ 1.412E+11	1.397E+11/ 1.413E+11	1.397E+11/ 1.413E+11	1.400E+11/ 1.412E+11	1.413E+11/ 1.425E+11
SLD average vesicle tail i/o [cm^{-2}]	8.331E+10/ 8.076E+10	8.250E+10/ 8.009E+10	8.230E+10/ 7.987E+10	8.098E+10/ 7.929E+10	7.544E+10/ 7.578E+10
SLD average head surface bicelle [cm^{-2}]	1.435E+11	1.436E+11	1.430E+11	1.435E+11	1.419E+11
SLD average head rim bicelle [cm^{-2}]	1.443E+11	1.442E+11	1.445E+11	1.442E+11	1.435E+11
SLD average tail bicelle [cm^{-2}]	7.655E+10	7.565E+10	7.543E+10	7.493E+10	7.436E+10

Table S24: *Model fit parameters from analysis of the SAXS data from 2.5mg/ml DPPC vesicles mixed with 2.5mg/ml DDM at different temperatures*

**Model fits of SAXS data from 2.5mg/ml DPPC vesicles mixed with 2.5 mg/ml DDM at different temperatures**					
	10°C	20°C	25°C	30°C	45°C
**Vesicle + bicelle model**					
Average radius vesicle [Å]	312	294	315	335	220

Thickness of i/o tail shell vesicle [Å]	16.0/ 15.3	16.0/ 15.3	16.0/ 15.3	15.3/ 13.4	14.1/ 13.2
Thickness of i/o head shell vesicle [Å]	9.0/9.6	9.0/9.6	9.0/9.6	9.5/10	9.5/10.0
Smearing (i/o tail region) vesicle [Å]	5.0/5.0	5.0/5.0	5.0/5.0	4.4/4.4	4.4/4.4
Smearing (i/o head region) vesicle [Å]	3.0/2.0	3.0/2.0	3.0/2.0	3.0/2.0	3.0/2.0
Hydration of i/o head shell vesicle*	0.256/ 0.207	0.256/ 0.218	0.263/ 0.219	0.339/ 0.280	
Volume lipid (o/i) vesicle [Å^3]	1155/ 1158	1156/ 1172	1159/ 1175	1175/ 1189	1175/ 1186
Fraction of DDM in inner leaflet	0.04	0.04	0.04	0.17	0.19
Polydispersity vesicle (gaussian model)	0.32	0.35	0.26	0.25	0.8
Fraction of unilamellar vesicles	0.9	0.9	0.9	0.9	1
Nu	0	0	0	0.5	NA
Number of bilayers	1.5	1.5	1.5	1.5	NA
Sigma_d	3.0	3	3	3.0	NA
Distance between lamella [Å]	61	61	61	61	NA
Eta	0.1	0.1	0.1	0.1	NA
Fraction lipid/DDM in bicelles	0.20/ 0.90	0.20/ 0.90	0.20/ 0.90	0.28/ 0.87	0.79/ 0.88
Radius bicelle [Å]	28.5	27.5	28.8	33.3	43.0
Thickness of bicelle tail region disc [Å]	23.6	23.6	23.6	24.1	25.5
Thickness of bicelle head shell [Å]	6.0	6.0	6.0	10.0	10.5
Thickness of bicelle rim [Å]	9.0	9.0	9.0	9.0	10.0
Volume lipid in bicelle [Å^3]	1242	1248	1256	1250	1253
Hydration of head shell/sim*	0.578/ 0.205	0.594/ 0.206	0.500/ 0.286	0.690/ 0.315	0.851/ 0.317
Fraction of lipid/DDM in rim	0.13/ 0.39	0.13/ 0.39	0.13/ 0.46	0.19/ 0.46	0.79/ 0.88
Interface smearing	4.0	4.0	3.5	4.0	3.3
SLD average vesicle head i/o [cm^{-2}]	1.397E+11/ 1.396E+11	1.397E+11/ 1.413E+11	1.397E+11/ 1.413E+11	1.402E+11/ 1.415E+11	1.412E+11/ 1.429E+11
SLD average vesicle tail i/o [cm^{-2}]	8.270E+10/ 8.130E+10	8.260E+10/ 8.013E+10	8.231E+10/ 7.993E+10	8.055E+10/ 7.865E+10	7.999E+10/ 7.773E+10
SLD average head surface bicelle [cm^{-2}]	1.441E+11	1.441E+11	1.440E+11	1.437E+11	1.428E+11
SLD average head rim bicelle [cm^{-2}]	1.446E+11	1.446E+11	1.446E+11	1.444E+11	1.444E+11
SLD average tail bicelle [cm^{-2}]	7.449E+10	7.436E+10	7.419E+10	7.430E+10	7.412E+10

Table S25: *Model fit parameters from analysis of the SAXS data from 2.5mg/ml DPPC vesicles mixed with 5mg/ml DDM at different temperatures*

Model fits of SAXS data from 2.5mg/ml DPPC vesicles mixed with 5 mg/ml DDM at different temperatures				
	10°C	20°C	25°C	30°C
**Vesicle + bicelle model**				
Average radius vesicle [Å]	310	290	318	333
Thickness of i/o tail shell vesicle [Å]	16.0/15.3	16.0/15.3	16.0/15.3	16/15.3
Thickness of i/o head shell vesicle [Å]	9.0/9.6	9.0/9.6	9.0/9.6	9.0/9.6
Smearing (i/o tail region) vesicle [Å]	5.0/5.0	5.0/5.0	5.0/5.0	5.0/5.0
Smearing (i/o head region) vesicle [Å]	3.0/2.0	3.0/2.0	3.0/2.0	3.0/2.0
Hydration of i/o head shell vesicle*	0.277/ 0.118	0.269/ 0.158	0.279/ 0.155	0.296/ 0.121
Volume lipid (o/i) vesicle [Å^3]	1156/1172	1158/1178	1161/1182	1171/1190
Fraction of DDM in inner leaflet	0.04	0.04	0.04	0.04
Polydispersity vesicle (gaussian model)	0.33	0.34	0.25	
Fraction of unilamellar vesicles	0.9	0.9	0.9	0.9
Nu	0.0	0.0	0.0	0
Number of bilayers	1.5	1.5	1.5	1.5
Sigma_d	3.0	3.0	3.0	3.0
Distance between lamella [Å]	61	61	61	61
Eta	0.1	0.1	0.1	0.1
Fraction lipid/DDM in bicelles	0.27/0.93	0.29/0.94	0.35/0.95	0.47/0.95
Radius bicelle [Å]	26.3386	25.3	26.9	29.8
Thickness of bicelle tail region disc [Å]	23.1	23.3	24.3	24.3
Thickness of bicelle head shell [Å]	6.0	6.0	6.0	6.0
Thickness of bicelle rim [Å]	9.0	9.0	9.0	9.0
Volume lipid in bicelle [Å^3]	1256	1260	1262	1275
Hydration of head shell/sim*	0.549/ 0.229	0.562/ 0.240	0.548/ 0.210	0.511/ 0.261
Fraction of lipid/DDM in rim	0.19/0.42	0.19/0.43	0.19/0.42	0.20/0.43
Interface smearing	4.0	4.0	4.0	4.0
SLD average vesicle head i/o [cm^{-2}]	1.398E+11/ 1.418E+11	1.398E+11/ 1.416E+11	1.397E+11/ 1.416E+11	1.398E+11/ 1.418E+11
SLD average vesicle tail i/o [cm^{-2}]	8.258E+10/ 7.976E+10	8.239E+10/ 7.943E+10	8.214E+10/ 7.914E+10	8.109E+10/ 7.844E+10
SLD average head surface bicelle [cm^{-2}]	14427	1.442E+11	1.442E+11	1.440E+11
SLD average head rim bicelle [cm^{-2}]	14459	1.446E+11	1.446E+11	1.444E+11
SLD average tail bicelle [cm^{-2}]	7.424E+10	7.416E+10	7.410E+10	7.373E+10

Table S26: *Model fit parameters from analysis of the SAXS data from 2.5mg/ml DPPC vesicles mixed with 10mg/ml DDM at different temperatures*

Model fits of SAXS data from 2.5mg/ml DPPC vesicles mixed with 10 mg/ml DDM at different temperatures				
	10°C	20°C	25°C	30°C
**Vesicle + bicelle model**				
Average radius vesicle [Å]	318	302	307	350
Thickness of i/o tail shell vesicle [Å]	15.8/15.1	16.0/15.3	16.0/15.3	16.0/15.3
Thickness of i/o head shell vesicle [Å]	9.0/9.6	9.0/9.6	9.0/9.6	9.0/10.4
Smearing (i/o tail region) vesicle [Å]	5.0/5.0	5.0/5.0	5.0/5.0	5.0/5.0
Smearing (i/o head region) vesicle [Å]	3.0/2.0	3.0/2.0	3.0/2.0	3.0/2.0

Hydration of i/o head shell vesicle*	0.297/ 0.074	0.286/ 0.051	0.288/ 0.079	0.335/ 0.009
Volume lipid (o/i) vesicle [Å^3]	1158/1167	1156/1172	1162/1178	1181/1200
Fraction of DDM in inner leaflet	0.04	0.04	0.04	0.04
Polydispersity vesicle (gaussian model)	0.32	0.34	0.28	0.23
Fraction of unilamellar vesicles	0.9	0.9	0.9	0.9
Nu	0	0	0	0
Number of bilayers	1.5	1.5	1.5	1.5
Sigma_d	3.0	3.0	3.0	3.0
Distance between lamella [Å]	61	61	61	61
Eta	0.1	0.1	0.1	0.1
Fraction lipid/DDM in bicelles	0.43/0.97	0.55/0.97	0.54/0.97	0.75/0.98
Radius bicelle [Å]	24.6	24.6	25.1	27.0
Thickness of bicelle tail region disc [Å]	23.7	23.4	24.0	24.5
Thickness of bicelle head shell [Å]	8.0	8.0	8.0	8.0
Thickness of bicelle rim [Å]	9.0	9.0	9.0	9.0
Volume lipid in bicelle [Å^3]	1253	1265	1280	1285
Hydration of head shell/rim*	0.721/ 0.119	0.753/ 0.115	0.734/ 0.113	0.706/ 0.160
Fraction of lipid/DDM in rim	0.12/0.37	0.14/0.34	0.14/0.35	0.16/0.38
Interface smearing	4.2	4.2	4.2	4.2
SLD average vesicle head i/o [cm^{-2}]	1.398E+11/ 1.420E+11	1.398E+11/ 1.420E+11	1.398E+11/ 1.419E+11	1.399E+11/ 1.425E+11
SLD average vesicle tail i/o [cm^{-2}]	8.236E+10/ 7.997E+10	8.255E+10/ 7.954E+10	8.200E+10/ 7.920E+10	8.010E+10/ 7.728E+10
SLD average head surface bicelle [cm^{-2}]	1.444E+11	1.443E+11	1.446E+11	1.446E+11
SLD average head rim bicelle [cm^{-2}]	1.447E+11	1.446E+11	1.443E+11	1.442E+11
SLD average tail bicelle [cm^{-2}]	7.430E+10	7.410E+10	7.388E+10	7.364E+10

Table S27: *Model fit parameters from analysis of the SAXS data from 2.5mg/ml DPPC vesicles mixed with 10mg/ml DDM at different temperatures*

**Model fits of SAXS data from 2.5mg/ml DPPC vesicles mixed with 5mg/ml and 10 mg/ml DDM at 45°C**		
	5 mg/ml DDM	10 mg/ml DDM
**Rod/worm-like micelle model**		
Average radius rod [Å]	17.5	16.2
Length of rod [Å]	1000	277.3
Thickness of head shell [Å]	9.0	8.2
Smearing (i/o tail region) [Å]	1.6	0.8
Smearing (i/o head region) [Å]	1.9	2.4
Volume lipid (o/i) vesicle [Å^3]	1194	1196
Hydration of head shell*	0.467	0.424
Polydispersity radius (gaussian model)	0.14	0.17
Statistical segment length	900	NA
SLD average head [cm^{-2}]	1.439E+11	1.445E+11
SLD average tail [cm^{-2}]	7.792E+10	7.754E+10

S3.5: DMPC:DDM mixtures

Three different analytical scattering models were used to analyse the scattering data from the DMPC:DDM: a 4-shell vesicle model which accounts for the inserted DDM molecules, the vesicles in coexistence with bicelles and a rod/worm-like micelle model. The scattering data along with the model fits at different ratios and temperatures is shown in Figure S9a). Inverse Fourier transform analysis was also used to give an indication of which structures were present at the 2:1 ratio at 10°C, shown in Figure S8b) and for the worm-like structures at the highest ratio in Figure S9c). Tables S28 to S31 show the resultant fit parameters.

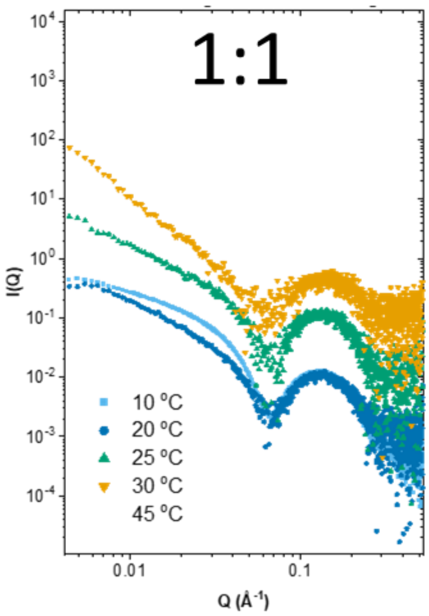

Figure S10: *SAXS measurements of DMPC mixed with DDM in a 1:1 mass ratio.*

Table S28: *Model fit parameters from analysis of the SAXS data from 2.5mg/ml DMPC vesicles mixed with 0.31mg/ml DDM at different temperatures*

**Model fits of SAXS data from 2.5mg/ml DMPC vesicles mixed with 0.31 mg/ml DDM at different temperatures**				
	10°C	20°C	25°C	30°C
**Mixed vesicle model**				
Average radius vesicle [Å]	280	311	255	235
Thickness of i/o tail shell vesicle [Å]	13.5/10.7	13.0/10.7	11.0/9.0	11.0/9.5
Thickness of i/o head shell vesicle [Å]	9.0/12.0	10.0/11.2	10.7	10.7
Smearing (i/o tail region) vesicle [Å]	3.0/3.0	4.0/3.0	1.7	0.5
Smearing (i/o head region) vesicle [Å]	3.0/4.0	3.0/4.5	3.0/3.0	1.0/1.5
Hydration of i/o head shell vesicle*	0.354/0.474	0.452/0.456	0.480/0.494	0.480/0.530
Volume lipid (i/o) vesicle [Å^3]	1045/1060	1065/1085	1097	1101
Fraction of DDM in inner leaflet	0	0	(symmetric)	(symmetric)
Fraction of unilamellar vesicles	0.95	0.80	0.95	0.95
Nu	0.1	0.1	0.1	1.0
Number of bilayers	2.0	5.0	2.0	2.0
Sigma_d	1.0	1.0	1.0	1.0
Distance between lamella [Å]	71	68.5	63.0	63.0

Eta	0.1	0.1	0.1	0.1
Polydispersity vesicle (gaussian model)	0.30	0.22	0.37	0.40
SLD average head i/o [cm^{-2}]	1.396E+11/ 1.411E+11	1.396E+11/ 1.411E+11	1.405E+11	1.405E+11
SLD average tail i/o [cm^{-2}]	8.288E+10/ 8.057E+10	8.062E+10/ 7.828E+10	7.723E+10	7.686E+10

Table S29: *Model fit parameters from analysis of the SAXS data from 2.5mg/ml DMPC vesicles mixed with 0.63mg/ml DDM at different temperatures*

**Model fits of SAXS data from 2.5mg/ml DMPC vesicles mixed with 0.63 mg/ml DDM at different temperatures**					
	10°C	20°C	25°C	30°C	45°C
**Mixed vesicle model**					
Average radius vesicle [Å]	200	340	295	265	313
Thickness of i/o tail shell vesicle [Å]	11.7/10.1	12.0/10.1	11.0/9.7	10.7/9.2	9.0
Thickness of i/o head shell vesicle [Å]	10.0/11.2	10.0/11.2	10.0/11.2	10.0/11.0	10.0
Smearing (i/o tail region) vesicle [Å]	3.5/3.5	3.5/3.5	3.0/3.0	2.0/2.0	3.2
Smearing (i/o head region) vesicle[Å]	3.0/4.5	3.0/4.5	2.2/3.2	2.2/3.2	2.8/3.2
Hydration of i/o head shell vesicle*	0.465/ 0.458	0.474/ 0.459	0.496/ 0.517	0.490/ 0.551	0.539/ 0.567
Volume lipid (i/o) vesicle [$Å^3$]	1065/ 1078	1085/ 1105	1097 /1112	1103/ 1110	1119/ 1121
Fraction of DDM in inner leaflet	0.12	0.20	0.25	0.33	0.40
Fraction of unilamellar vesicles	0.90	0.65	0.80	0.90	0.75
Nu	0.1	0.1	0.1	0.1	0
Number of bilayers	3.0	5.0	3.5	3.5	5
Sigma_d	1.0	1.0	1.0	1.0	0.1
Distance between lamella [Å]	73	67.0	62.0	60.0	58.0
Eta	0.2	0.2	0.2	0.2	0.01
Polydispersity vesicle (gaussian model)	0.80	0.3	0.30	0.37	0.26
SLD average head i/o [cm^{-2}]	1.401E+11/ 1.418E+11	1.403E+11/ 1.417E+11	1.405E+11/ 1.416E+11	1.407E+11/ 1.415E+11	1.409E+11/ 1.413E+11
SLD average tail i/o [cm^{-2}]	8.049E+10/ 7.872E+10	7.839E+10/ 7.656E+10	7.723E+10/ 7.600E+10	7.669E+10/ 7.614E+10	7.531E+10/ 7.523E+10

Table S30: *Model fit parameters from analysis of the SAXS data from 2.5mg/ml DMPC vesicles mixed with 1.25mg/ml DDM at different temperatures*

**Model fits of SAXS data from 2.5mg/ml DMPC vesicles mixed with 1.25 mg/ml DDM at different temperatures**					
	10°C	20°C	25°C	30°C	45°C
**Mixed vesicle + bicelle model**		**Mixed vesicle model**			
Average radius vesicle [Å]	290	140	310	280	320

Thickness of i/o tail shell vesicle [Å]	10.5/10.1	9.5/9.0	9.5/9.0	9.0/8.5	9.0/8.5
Thickness of i/o head shell vesicle [Å]	10.5/11.5	11.0/14.0	11.0/14.0	11.0/14.0	11.0/13.0
Smearing (i/o tail region) vesicle [Å]	3.5/3.5	3.5/3.5	3.0/3.0	3.0/3.0	3.0/3.0
Smearing (i/o head region) vesicle [Å]	3.0/4.5	4.0/5.0	4.0/4.5	4.0/4.0	3.0/4.0
Hydration of i/o head shell vesicle*	0.517/ 0.466	0.499/ 0.661	0.554/ 0.619	0.580/ 0.640	0.571/ 0.595
Volume lipid (i/o) vesicle [$Å^3$]	1066/ 1077	1120/ 1115	1118/ 1113	1120/ 1113	1128/ 1120
Fraction of DDM in inner leaflet	0.21	0.36	0.34	0.33	0.35
Fraction of unilamellar vesicles	0.85	0.55	0.59	0.80	0.50
Nu	0	0.5	0.2	0	0
Number of bilayers	2.0	4.0	4.0	4.0	5.0
Sigma_d	1.0	1.0	0.5	0.5	0.5
Distance between lamella [Å]	64	67.0	64.0	61.0	59.0
Eta	0.1	0.2	0.2	0.2	0.2
Polydispersity vesicle (gaussian model)	0.59	0.9	0.40	0.37	0.37
Fraction lipid/DDM in bicelles	0.45/0.66	NA	NA	NA	NA
Radius bicelle [Å]	50.9	NA	NA	NA	NA
Thickness of bicelle tail region disc [Å]	27.6	NA	NA	NA	NA
Thickness of bicelle head shell [Å]	10.0	NA	NA	NA	NA
Thickness of bicelle rim [Å]	12.0	NA	NA	NA	NA
Volume lipid in bicelle [$Å^3$]	1035	NA	NA	NA	NA
Hydration of head shell/rim*	0.463/0.829	NA	NA	NA	NA
Fraction of lipid/DDM in rim	0.15/0.33	NA	NA	NA	NA
SLD average head vesicle i/o [cm^{-2}]	1.430E+11/ 1.481E+11	1.416E+11/ 1.424E+11	1.415E+11/ 1.424E+11	1.415E+11/ 1.424E+11	1.418E+11/ 1.426E+11
SLD average tail vesicle i/o [cm^{-2}]	7.933E+10/ 7.614E+10	7.537E+10/ 7.591E+10	7.551E+10/ 7.606E+10	7.534E+10/ 7.606E+10	7.481E+10/ 7.565E+10
SLD average head bicelle surface [cm^{-2}]	1.417E+11	NA	NA	NA	NA
SLD average head bicelle rim i/o [cm^{-2}]	1.430E+11	NA	NA	NA	NA
SLD average tail bicelle [cm^{-2}]	7.954E+10	NA	NA	NA	NA

Table S31: *Model fit parameters from analysis of the SAXS data from 2.5mg/ml DMPC vesicles mixed with 5mg/ml DDM at different temperatures*

**Model fits of SAXS data from 2.5mg/ml DPPC vesicles mixed with 5mg/ml DDM at different temperatures**					
	10°C	20°C	25°C	30°C	45°C
**Rod/worm-like micelle model**					
Average radius rod [Å]	19.1	16.8	16.8	16.6	16.3
Length of rod [Å]	322	800	800	800	1000
Thickness of head shell [Å]	6.5	10.6	10.6	10.6	10.8
Smearing (tail region) [Å]	0.87	0.2	0.2	0.2	0.1
Smearing (head region) [Å]	1.2	1.6	1.3	0.7	0.1
Volume lipid (o/i) vesicle [$Å^3$]	1108	1106	1111	1116	1124
Hydration of head shell*	0.063	0.529	0.531	0.539	0.558
Statistical segment length	NA	700	700	700	450
SLD average head [cm^{-2}]	1.367E+11	1.438E+11	1.438E+11	1.438E+11	1.438E+11
SLD average tail [cm^{-2}]	8.071E+10	7.910E+10	7.910E+10	7.882E+10	7.872E+10

S3.6: DPPC:TX-100 mixtures

Two different analytical scattering models have been used to analyse the scattering data from the DPPC:TX-100 mixtures: the mixed vesicle model which accounts for the inserted TX-100 molecules as described in section S2.1 and a bicelle (disc) model described in S2.2. Inverse Fourier transform analysis was used to give an indication of which structures were present at the highest temperatures, where the analytical models failed to give a fit, showing elongated structures to be present. For the low temperature measurements where we have the appearance of overlapping Bragg peaks in the scattering pattern we have large rippled leaftlets which cannot be described by any analytical model, but has been studied further using cryo-TEM in a separate publication.

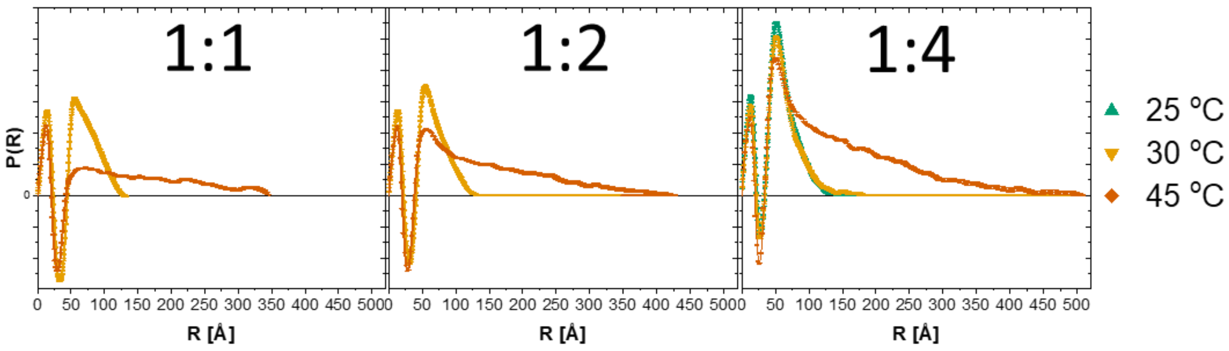

Figure S11: *Distance distribution functions of the 1:1, 1:2 and 1:4 ratio of DPPC:TX-100 mixtures at different temperatures showing that the structure at 45°C is much more extended than at the other temperatures*

Table S32: *Model fit parameters from analysis of the SAXS data from 2.5mg/ml DPPC vesicles mixed with different TX-100 concentrations at 10°C.*

**Model fits of SAXS data from 2.5mg/ml DPPC vesicles mixed with different TX-100 concentrations at 10°C**						
	0.31 mg/ml	0.63 mg/ml	1.25 mg/ml	2.5 mg/ml	5 mg/ml	10 mg/ml

Mixed vesicle + TX-100 micelle model						
Average radius [Å]	300	490	490	490	490	490
Thickness of i/o tail shell [Å]	15.2	15.6	15.4	14.8	13.2	11.5
Thickness of i/o head shell [Å]	9.0/7.5	9.0/9.0	9.0/9.0	9.0/9.0	9.0/9.0	9.7/9.0
Smearing (i/o tail region) [Å]	4.1	4.1	4.1	4.1	4.1	4.1
Smearing (i/o head region) [Å]	4.0/3.0	3.0/2.0	3.0/2.0	3.0/2.0	3.0/2.0	3.0/3.0
Hydration of i/o head shell*	0.075/ 0.126	0.139/ 0.263	0.136/ 0.261	0.099/ 0.226	0.052/ 0.180	0.087/ 0.135
Volume lipid (o/i) [$Å^3$]	1140	1132	1135	1140	1155	1189
Fraction of unilamellar vesicles	0.55	0	0	0	0	0
Nu	2.0	2.0	2	2	2	1.4
Number of bilayers	3.8	5.6	5.6	5.6	5.6	5
Sigma_d	1	2	2	2	2	2
Distance between lamella [Å]	63.5	64	64	64	64	64
Eta	0.15	0.05	0.05	0.05	0.05	0.05
Polydispersity (gaussian model)	0.33	0.45	0.45	0.45	0.45	0.45
Fraction of TX-100 in micelles	0	0.70	0.84	0.83	0.85	0.825
SLD average head [cm^{-2}]	1.351E+11	1.366E+11	1.365E+11	1.338E+11	1.311E+11	1.265E+11
SLD average tail [cm^{-2}]	8.277E+10	8.361E+10	8.331E+10	8.272E+10	8.131E+10	7.888E+10

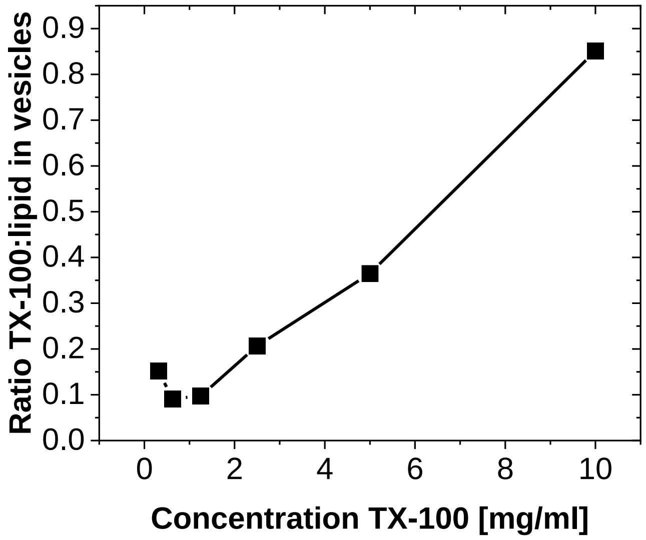

Figure S12: *The ratio of TX-100 to lipid in the vesicle structure plotted as a function of the mass concentration of TX-100 present in solution.*

Table S33: *Model fit parameters from analysis of the SAXS data from 2.5mg/ml DPPC vesicles mixed with 0.31mg/ml TX-100 at 20°C.*

**Model fits of SAXS data from 2.5mg/ml DPPC vesicles mixed with 0.31mg/ml TX-100 at 20°C**	
	0.31mg/ml
**Mixed vesicle model**	
Average radius [Å]	290
Thickness of i/o tail shell [Å]	15.2
Thickness of i/o head shell [Å]	9.0/7.5
Smearing (i/o tail region) [Å]	3.6
Smearing (i/o head region) [Å]	4.5/3.0
Hydration of i/o head shell*	0.126/0.136
Volume lipid (o/i) [$Å^3$]	1147
Fraction of unilamellar vesicles	0.70
Nu	1.0
Number of bilayers	3.8
Sigma_d	1
Distance between lamella [Å]	64.5
Eta	0.15
Polydispersity (gaussian model)	0.39
SLD average head [cm^{-2}]	1.351E+11
SLD average tail [cm^{-2}]	8.210E+10

Table S34: *Model fit parameters from analysis of the SAXS data from 2.5mg/ml DPPC vesicles mixed with different concentrations of TX-100 at 45°C.*

**Model fits of SAXS data from 2.5mg/ml DPPC vesicles mixed with different TX-100 concentrations at 45°C**		
	0.31mg/ml	0.63mg/ml
**Mixed vesicle model**		
Average radius [Å]	300	300
Thickness of i/o tail shell [Å]	12.3	11.1
Thickness of i/o head shell [Å]	9.0/7.5	9.0/7.5
Smearing (i/o tail region) [Å]	2.0	2.2
Smearing (i/o head region) [Å]	4.0/2.0	4.0/3.0
Hydration of i/o head shell*	0.308/0.323	0.293/0.299
Volume lipid (o/i) [$Å^3$]	1203	1210
Polydispersity (gaussian model)	0.33	0.33
SLD average head [cm^{-2}]	1.336E+11	1.297E+11
SLD average tail [cm^{-2}]	7.715E+10	7.681E+10

Table S35: *Model fit parameters from analysis of the SAXS data from 2.5mg/ml DPPC vesicles mixed with 10mg/ml TX-100 at 25°C.*

**Model fits of SAXS data from 2.5mg/ml DPPC vesicles mixed with different concentrations of TX-100 at 25°C**	
	10mg/ml
**Bicelle + TX-100 micelle model**	
Radius bicelle [Å]	35.7
Thickness of bicelle tail region disc [Å]	17.6
Thickness of bicelle head shell [Å]	17.6
Thickness of bicelle rim [Å]	25.0
Volume lipid in bicelle [$Å^3$]	1151
Hydration of head shell/rim*	0.678/0.939
Fraction of lipid/TX-100 in rim	0.0/0.44
Fraction of TX-100 in ellipsoidal micelle	0.52
SLD average head bicelle surface [cm^{-2}]	1.225E+11
SLD average head bicelle rim i/o [cm^{-2}]	1.146E+11
SLD average tail bicelle [cm^{-2}]	8.132E+10

Table S36: *Model fit parameters from analysis of the SAXS data from 2.5mg/ml DPPC vesicles mixed with different concentrations of TX-100 at 30°C.*

**Model fits of SAXS data from 2.5mg/ml DPPC vesicles mixed with different concentrations of TX-100 at 30°C**			
	2.5mg/ml	5mg/ml	10mg/ml
**Bicelle model**		**Bicelle + TX-100 micelle model**	
Radius bicelle [Å]	41.8	38.0	37.1
Thickness of bicelle tail region disc [Å]	23.6	20.0	16.1
Thickness of bicelle head shell [Å]	16.8	17.7	17.7
Thickness of bicelle rim [Å]	27.4	29.0	29.0
Volume lipid in bicelle [$Å^3$]	1148	1153	1149
Hydration of head shell/rim*	0.591/0.950	0.641/0.905	0.675/0.954
Fraction of lipid/TX-100 in rim	0.13/0.34	0.0/0.40	0.0/0.38

Fraction of TX-100 in ellipsoidal micelle	0	0.30	0.46
SLD average head bicelle surface [cm^{-2}]	1.244E+11	1.235E+11	1.207E+11
SLD average head bicelle rim i/o [cm^{-2}]	1.180E+11	1.138E+11	1.138E+11
SLD average tail bicelle [cm^{-2}]	8.169E+10	8.128E+10	8.138E+10

S3.7: DMPC:TX-100 mixtures

Three different analytical scattering models were used to analyse the scattering data from the DMPC:TX-100 mixtures: a 4-shell vesicle model which accounts for the inserted TX-100 molecules, a bicelle model and a model with coexistence between these bicelles and vesicles. These models could not account for all the structures observed. Inverse Fourier transform analysis was also used to give an indication of which structures were present, particularly where the analytical models failed to give a fit. Figure S11 shows the scattering curves for all different ratios as indicated above the graphs and temperatures as indicated in the lower left of the figure with the obtained fits. Tables S36 to S40 show the resultant fit parameters.

Table S37: *Model fit parameters from analysis of the SAXS data from 2.5mg/ml DMPC vesicles mixed with 0.31mg/ml of TX-100 at different temperatures.*

**Model fits of SAXS data from 2.5mg/ml DMPC vesicles mixed with 0.31 mg/ml TX-100 at different temperatures**			
	25°C	30°C	45°C
**Vesicle model**			
Average radius [Å]	400	325	200
Thickness of i/o tail shell [Å]	12.1	11.0	10.0
Thickness of i/o head shell [Å]	9.0/7.0	9.0/7.0	9.0/7.0
Smearing (i/o tail region) [Å]	2.7	3.0	4.0
Smearing (i/o head region) [Å]	2.4	2.4	4.0/2.4
Hydration of i/o head shell*	0.257/0.180	0.324/0.270	0.366/0.370
Volume lipid (o/i) [$Å^3$]	1096	1105	1117
Fraction of unilamellar vesicles	1	0.7	0.1
Nu	NA	1.0	1.5
Number of bilayers	NA	2.0	4.0
Sigma_d	NA	3	2
Distance between lamella [Å]	NA	76	75
Eta	NA	0.40	0.18
Polydispersity (gaussian model)	0.45	0.39	0.39
SLD average head [cm^{-2}]	1.337E+11	1.337E+11	1.337E+11
SLD average tail [cm^{-2}]	7.796E+10	7.710E+10	7.600E+10

Table S38: *Model fit parameters from analysis of the SAXS data from 2.5mg/ml DMPC vesicles mixed with 0.63mg/ml of TX-100 at different temperatures.*

**Model fits of SAXS data from 2.5mg/ml DMPC vesicles mixed with 0.63 mg/ml TX-100 at different temperatures**				
	10°C	25°C	30°C	45°C
**Vesicle model**				
Average radius [Å]	250	280	280	250
Thickness of i/o tail shell [Å]	13.9	12.1	11.1	10.5
Thickness of i/o head shell [Å]	10.0/7.8	9.0/7.0	9.0/7.0	8.0/7.0

Smearing (i/o tail region) [Å]	3.0	3.0	3.2	3.2
Smearing (i/o head region) [Å]	2.2/1.8	2.4	2.4	2.4
Hydration of i/o head shell*	0.025/ 0.045	0.132/ 0.099	0.215/ 0.180	0.175/ 0.237
Volume lipid (o/i) [Å^3]	1056	1100	1107	1121
Fraction of unilamellar vesicles	1	1	1	0.4
Nu	NA	NA	NA	0.5
Number of bilayers	NA	NA	NA	3
Sigma_d	NA	NA	NA	2
Distance between lamella [Å]	NA	NA	NA	87
Eta	NA	NA	NA	0.24
Polydispersity (gaussian model)	0.7	1.0	1.0	0.50
SLD average head [cm^{-2}]	1.297E+11	1.297E+11	1.297E+11	1.297E+11
SLD average tail [cm^{-2}]	8.234E+10	7.813E+10	7.750E+10	7.626E+10

Table S39: *Model fit parameters from analysis of the SAXS data from 2.5mg/ml DMPC vesicles mixed with 1.25mg/ml of TX-100 at different temperatures.*

**Model fits of SAXS data from 2.5mg/ml DMPC vesicles mixed with 1.25 mg/ml TX-100 at different temperatures**			
	25°C	30°C	45°C
**Vesicle + bicelle model**			
Average radius [Å]	200	124	124
Thickness of i/o tail shell [Å]	11.5	11.2	10.3
Thickness of i/o head shell [Å]	9.8/7.2	9.8/7.2	8.5/6.5
Smearing (i/o tail region) [Å]	3.5	3.5	3.8
Smearing (i/o head region) [Å]	3.2/2.4	3.2/2.4	2.0/2.4
Hydration of i/o head shell*	0.061/0.040	0.010/0.123	0.000/0.148
Volume lipid (o/i) [Å^3]	1112	1115	1127
Polydispersity (gaussian model)	0.95	1.3	1.2
Fraction lipid/TX-100 in bicelles	0.2/0.2	0.15/0.15	0.0/0.0
Radius bicelle [Å]	50	50	NA
Thickness of bicelle tail region disc [Å]	16.0	16.0	NA
Thickness of bicelle head shell [Å]	11.0	11.0	NA
Thickness of bicelle rim [Å]	13.0	13.0	NA
Volume lipid in bicelle [Å^3]	1082	1097	NA
Hydration of head shell/sim*	0.8073/0.717	0.810/0.721	NA
Fraction of lipid/TX-100 in rim	0.0/0.7	0.0/0.7	NA
Interface smearing	3.0	3.0	NA
SLD average head vesicle [cm^{-2}]	1.248E+11	1.248E+11	1.248E+11
SLD average tail vesicle [cm^{-2}]	7.809E+10	7.785E+10	7.685E+10
SLD average head bicelle surface [cm^{-2}]	1.328E+11	1.328E+11	1.240E+11
SLD average head bicelle rim [cm^{-2}]	1.098E+11	1.098E+11	1.098E+11
SLD average tail bicelle [cm^{-2}]	8.060E+10	7.933E+10	8.806E+10

Table S40: *Model fit parameters from analysis of the SAXS data from 2.5mg/ml DMPC vesicles mixed with 2.5mg/ml of TX-100 at different temperatures.*

**Model fits of SAXS data from 2.5mg/ml DMPC vesicles mixed with 2.5 mg/ml TX-100 at different temperatures**				
	10°C	20°C	30°C	45°C
**Bicelle model**			**Vesicle + bicelle model**	
Average radius vesicle [Å]	NA	NA	280	280
Thickness of i/o tail shell vesicle [Å]	NA	NA	8.5/6.9	8.5/6.9
Thickness of i/o head shell vesicle [Å]	NA	NA	10.3	10.3
Smearing (i/o tail region) vesicle [Å]	NA	NA	3.8	3.8
Smearing (i/o head region) vesicle [Å]	NA	NA	2.0/2.4	2.0/2.4
Hydration of i/o head shell vesicle*	NA	NA	0.001/0.000	0.025/0.006
Volume lipid (o/i) vesicle [Å^3]	NA	NA	1116	1126
Polydispersity vesicle (gaussian model)	NA	NA	0.65	0.65
Fraction lipid/TX-100 in bicelles	1.0/1.0	1.0/1.0	0.62/0.75	0.50/0.69
Radius bicelle [Å]	47.4	47.1	47.0	45.0
Thickness of bicelle tail region disc [Å]	19.9	18.1	16.2	13.0
Thickness of bicelle head shell [Å]	16.7	17.0	11.0	11.0
Thickness of bicelle rim [Å]	11.0	11.0	17.0	17.0
Volume lipid in bicelle [Å^3]	1061	1081	1108	1134
Hydration of head shell/sim*	0.633/0.850	0.680/0.864	0.838/0.683	0.853/0.749
Fraction of lipid/TX-100 in rim	0.0/0.4		0.0/0.4	0.0/0.4
Interface smearing	1.2	2.0	1.6	2.0
SLD average head vesicle [cm^{-2}]	NA	NA	1.227E+11	1.231E+11
SLD average tail vesicle [cm^{-2}]	NA	NA	7.835E+10	7.748E+10
SLD average head bicelle surface [cm^{-2}]	1.273E+11	1.273E+11	1.220E+11	1.214E+11
SLD average head bicelle rim [cm^{-2}]	1.150E+11	1.150E+11	1.098E+11	1.098E+11
SLD average tail bicelle [cm^{-2}]	8.117E+10	7.970E+10	8.046E+10	7.920E+10

Table S41: *Model fit parameters from analysis of the SAXS data from 2.5mg/ml DMPC vesicles mixed with 5.0mg/ml of TX-100 at different temperatures.*

**Model fits of SAXS data from 2.5mg/ml DMPC vesicles mixed with 5.0 mg/ml TX-100 at different temperatures**			
	10°C	20°C	45°C
**Bicelle model**			**Vesicle + bicelle model**
Average radius vesicle [Å]	NA	NA	280
Thickness of i/o tail shell vesicle [Å]	NA	NA	8.5/9.8
Thickness of i/o head shell vesicle [Å]	NA	NA	10.5
Smearing (i/o tail region) vesicle [Å]	NA	NA	3.3
Smearing (i/o head region) vesicle [Å]	NA	NA	3.0/2.4
Hydration of i/o head shell vesicle*	NA	NA	0.058/0.042
Volume lipid (o/i) vesicle [Å^3]	NA	NA	1126
Polydispersity vesicle (gaussian model)	NA	NA	
Fraction lipid/TX-100 in bicelles	NA	NA	0.65/0.79
Radius bicelle [Å]	44.1	43.5	35.0
Thickness of bicelle tail region disc [Å]	17.0	15.7	11.8
Thickness of bicelle head shell [Å]	18.5	17.8	11.0
Thickness of bicelle rim [Å]	9.0	15.0	16.4

Volume lipid in bicelle [Å^3]	1099	1118	1150
Hydration of head shell/sim*	0.581/0.880	0.641/0.905	0.853/0.737
Fraction of lipid/TX-100 in rim	0.0/0.2	0.0/0.3	0.0/0.4
Interface smearing	1.9	1.9	1.0
SLD average head vesicle [cm^{-2}]	NA	NA	1.186E+11
SLD average tail vesicle [cm^{-2}]	NA	NA	7.927E+10
SLD average head bicelle surface [cm^{-2}]	1.173E11	1.181E11	1.178E+11
SLD average head bicelle rim [cm^{-2}]	1.098E11	1.098E11	1.098E+11
SLD average tail bicelle [cm^{-2}]	8.225E10	8.120E10	8.046E+10

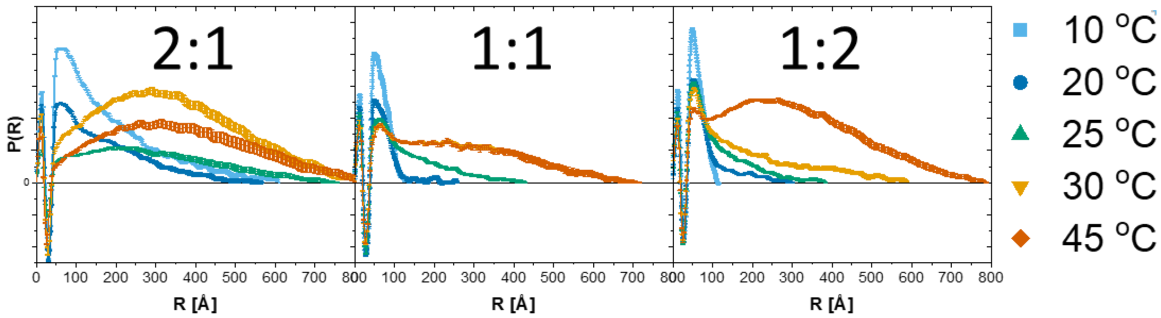

Figure S13: *Distance distribution functions of the 2:1, 1:1 and 1:2 ratio of DMPC:TX-100 mixtures at different temperatures as obtained from IFT analysis.*

S4: Analysis of kinetic SAXS data

S4.1: DPPC:SDS mixture at 20°C

Table S42: *Model fit parameters from analysis of the kinetic SAXS data from 2.5mg/ml DPPC vesicles mixed with 20mg/ml of SDS at 20°C at different timepoints from mixing.*

**Model fits of SAXS data from 2.5mg/ml DPPC vesicles mixed with 20.0 mg/ml SDS at 20°C**			
	400ms	40s	20min
**Mixed vesicle +ellipsoid model**			
Average radius vesicle [Å]	252	232	231
Thickness of i/o tail shell vesicle [Å]	16.1/16.1	14.9/14.9	15.4/15.4
Thickness of i/o head shell vesicle [Å]	10.0/7.1	10.0/7.1	10.0/7.1
Smearing (i/o tail region) vesicle [Å]	5.0/5.0	5.0/5.0	5.0/5.0
Smearing (i/o head region) vesicle [Å]	4.0/3.0	4.0/3.0	4.0/3.0
Hydration of i/o head shell vesicle*	0.266/0.230	0.334/0.305	0.414/0.394
Volume lipid (i/o) vesicle [Å^3]	1153	1158	1167
Polydispersity vesicle (gaussian model)	0.41	0.35	0.32
Fraction lipid/SDS in micelles	0/0.99	0/0.98	0.34/0.95
Equatorial radius [Å]	15.9	15.9	14.9
Aspect ratio	1.4	1.4	1.41
Thickness headgroup shell [Å]	5.7	5.4	6.9
Counter-ion fraction	0.93	0.93	0.93
Volume lipid in micelle [Å^3]	NA	NA	1164
Smearing	0.68	0.68	2.1

Hydration of head shell*	0.887	0.879	0.91
Hard-sphere radius [Å]	52	52	56
Z	2.7	2.7	2.7
Scale of structure factor	9.4	9.4	8.0
SLD average head vesicle [cm^{-2}]	1.432E+11	1.450E+11	1.629E+11
SLD average tail vesicle [cm^{-2}]	8.263E+10	8.203E+10	8.031E+10

S4.2: DMPC:SDS mixture at 20°C

Table S43: *Model fit parameters from analysis of the kinetic SAXS data from 2.5mg/ml DMPC vesicles mixed with 5mg/ml of SDS at 20°C at different timepoints from mixing.*

**Model fits of SAXS data from 2.5mg/ml DMPC vesicles mixed with 5 mg/ml SDS at 20°C**						
	200ms	600ms	2.2s	6.2s	10.2s	23.8s
Mixed vesicle model + bicelles						
Average radius vesicle [Å]	325	323	325	394	341	321
Thickness of i/o tail shell vesicle [Å]	10.3	10.3	10.3	12.3	12.3	12.3
Thickness of i/o head shell vesicle [Å]	6.5/5.4	6.5/5.4	6.5/5.4	9.9/8.4	9.9/8.4	9.9/8.4
Smearing (i/o tail region) vesicle [Å]	5.0/5.0	5.0/5.0	5.0/5.0	5.0/5.0	5.0/5.0	5.0/5.0
Smearing (i/o head region) vesicle [Å]	2.1/1.6	2.1/1.6	2.1/1.6	2.5/2.0	2.5/2.0	2.5/2.0
Hydration of i/o head shell vesicle*	0.541/ 0.529	0.563/ 0.552	0.541/ 0.529	0.571/ 0.570	0.566/ 0.576	0.559/ 0.573
Volume lipid (i/o) vesicle [$Å^3$]	1113	1113	1113	1034	1034	1034
Fraction of SDS in inner leaflet	0.5	0.5	0.5	0.5	0.5	0.5
Polydispersity vesicle (gaussian model)	1.10	0.89	1.06	0.43	0.43	0.43
Fraction lipid/SDS in bicelles	0.89/ 0.91	0.89/ 0.89	0.89/ 0.91	0.97/ 0.99	0.98/ 0.99	0.98/ 0.99
Radius bicelle [Å]	37.2	35.9	37.2	31.4	28.9	26.8
Thickness of bicelle tail region disc [Å]	19.4	20.2	19.4	21.8	21.8	22.1
Thickness of bicelle head shell [Å]	12.7	13.0	13.0	10.2	10.2	10.5

Thickness of bicelle rim [Å]	13.0	12.1	12.7	16.4	16.4	16.1
Volume lipid in bicelle [Å^3]	1067	1064	1067	1059	1047	1036
Hydration of head shell/sim*	0.981/ 0.828	0.980/ 0.810	0.981/ 0.828	0.981/ 0.771	0.980/ 0.780	0.979/ 0.788
Fraction of lipid/TX-100 in rim	0.03/ 0.28	0.03/ 0.28	0.03/ 0.28	0.03/ 0.38	0.03/ 0.46	0.03/ 0.51
Interface smearing	2.6	2.6	2.6	2.6	2.4	2.6
SLD average head vesicle [cm^{-2}]	2.002E+11	2.004E+11	1.932E+11	1.762E+11	1.765E+11	1.753E+11
SLD average tail vesicle [cm^{-2}]	7.564E+10	7.704E+10	7.694E+10	8.089E+10	8.087E+10	8.094E+10
SLD average head bicelle surface [cm^{-2}]	1.867E+11	1.867E+11	1.879E+11	1.834E+11	1.794E+11	1.766E+11
SLD average head bicelle rim [cm^{-2}]	2.546E+11	2.546E+11	2.545E+11	2.583E+11	2.602E+11	2.611E+11
SLD average tail bicelle [cm^{-2}]	7.871E+10	7.871E+10	7.860E+10	7.887E+10	7.930E+10	7.969E+10

S4.3: DPPC:DDM mixture at 20°C

Table S44: *Model fit parameters from analysis of the kinetic SAXS data from 2.5mg/ml DMPC vesicles mixed with 2.5mg/ml of DDM at 20°C at different timepoints from mixing*

**Model fits of SAXS data from 2.5mg/ml DPPC vesicles mixed with 2.5 mg/ml DDM at 20°C**		
	400ms	12min
Average radius vesicle [Å]	263	281
Thickness of i/o tail shell vesicle [Å]	14.9/14.9	15.3/14.7
Thickness of i/o head shell vesicle [Å]	11.4/6.1	11.4/6.3
Smearing (i/o tail region) vesicle [Å]	5.0/5.0	5.0/5.0
Smearing (i/o head region) vesicle [Å]	4.0/2.0	4.0/2.0
Hydration of i/o head shell vesicle*	0.394/0.134	0.416/0.020
Volume lipid (o/i) vesicle [Å^3]	1159/1159	1159/1172
Fraction of DDM in inner leaflet	NA	0.04
Polydispersity vesicle (gaussian model)	0.29	0.29
Fraction of unilamellar vesicles	0.5	0.65
Nu	0.5	0.5
Number of bilayers	2	2.0
Sigma_d	1	1.0
Distance between lamella [Å]	64	69
Eta	0.5	0.5
Fraction of DDM in DDM micelles	1	0
Fraction lipid/DDM in bicelles	NA	0.27/0.94
Radius bicelle [Å]	NA	31.3

Thickness of bicelle tail region disc [Å]	NA	23.1
Thickness of bicelle head shell [Å]	NA	7.9
Thickness of bicelle rim [Å]	NA	6.3
Volume lipid in bicelle [$Å^3$]	NA	1248
Hydration of head shell/sim*	NA	0.500/0.324
Fraction of lipid/DDM in rim	NA	0.13/0.51
Interface smearing	NA	4.0
SLD average head vesicle [cm^{-2}]	1.396E+11	1.3969174E+11
SLD average tail vesicle [cm^{-2}]	8.236E+10	8.23349695E+10
SLD average head bicelle surface [cm^{-2}]	NA	1.434E+11
SLD average head bicelle rim [cm^{-2}]	NA	1.445E+11
SLD average tail bicelle [cm^{-2}]	NA	7.43532273E+10

S4.4: DMPC:DDM mixture at 20°C

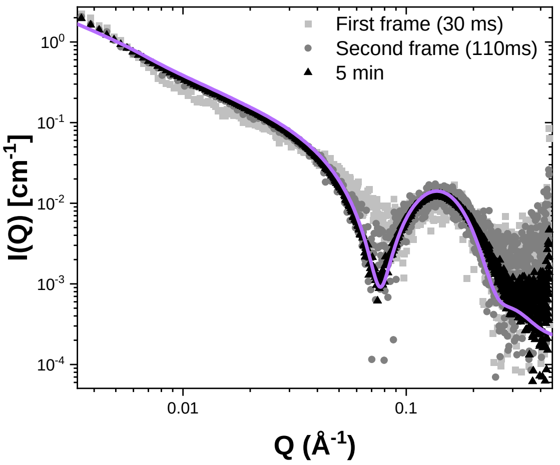

Figure S14: *Kinetics from a DMPC:DDM mixture at 1:2 ratio at 20°C. The fit parameters are the same as in Table S32.*

S4.5: DPPC:TX-100 mixtures at 10°C

Table S45: *Model fit parameters from analysis of the kinetic SAXS data from 2.5mg/ml DPPC vesicles mixed with 2.5mg/ml of TX-100 at 10°C at different timepoints from mixing*

**Model fits of SAXS data from 2.5mg/ml DPPC vesicles mixed with 2.5 mg/ml TX-100 at 10°C**						
	7min58s	18min	31min	44min	113min	2 days
**Vesicle model**						
Average radius [Å]	670	670	670	490	490	490
Thickness of i/o tail shell [Å]	14.9	14.9	14.9	14.9	14.9	14.9
Thickness of i/o head shell [Å]	10.5/ 8.5	10.5/ 8.5	10.5/ 8.5	10.5/ 8.5	10.5/ 8.5	10.5/ 8.5
Smearing (i/o tail region) [Å]	3.0	3.0	3.0	3.2	3.2	3.2
Smearing (i/o head region) [Å]	2.0/2.0	2.0/2.0	2.0/2.0	2.0/2.0	2.0/2.0	2.0/2.0

Hydration of i/o head shell*	0.290/ 0.217	0.290/ 0.218	0.290/ 0.218	0.276 /0.232	0.276/ 0.232	0.276/ 0.232
Volume lipid (o/i) [Å^3]	1145	1146	1146	1146	1146	1146
Fraction of unilamellar vesicles	0.85	0.85	0.78	0.54	0.44	0.38
Nu	0.5	1.0	1.0	1.5	1.5	1.5
Number of bilayers	2.0	3.0	4.0	5.0	5.0	5.0
Sigma_d	2.0	2.0	2.0	2.0	2.0	2.0
Distance between lamella [Å]	65	65	65	64	64	64
Eta	0.1	0.1	0.1	0.05	0.05	0.05
Polydispersity (gaussian model)	0.38	0.38	0.38	0.45	0.45	0.45
Fraction of TX-100 in micelles	0.89	0.89	0.89	0.89	0.89	0.89
SLD average head [cm^{-2}]	1.351E+11	1.351E+11	1.351E+11	1.351E+11	1.351E+11	1.351E+11
SLD average tail [cm^{-2}]	8.365E+10	8.355E+10	8.355E+10	8.355E+10	8.355E+10	8.355E+10

Table S46: *Model fit parameters from analysis of the kinetic SAXS data from 2.5mg/ml DPPC vesicles mixed with 5.0mg/ml of TX-100 at 10°C at different timepoints from mixing*

**Model fits of SAXS data from 2.5mg/ml DMPC vesicles mixed with 5.0 mg/ml TX-100 at 10°C**						
	4min33s	15min	28min	40min	104min	2 days
**Vesicle model**						
Average radius [Å]	670	670	670	490	490	490
Thickness of i/o tail shell [Å]	14.9	14.8	14.9	14.9	14.9	14.9
Thickness of i/o head shell [Å]	10.5/8.5	10.5/8.5	10.5/8.5	10.5/8.5	10.5/8.5	10.5/8.5
Smearing (i/o tail region) [Å]	3.0	3.0	3.0	3.0	3.2	3.2
Smearing (i/o head region) [Å]	2.0/2.0	2.0/2.0	2.0/2.0	2.0/2.0	2.0/2.0	2.0/2.0
Hydration of i/o head shell*	0.324/ 0.254	0.330/ 0.260	0.325/ 0.256	0.311/ 0.269	0.309/ 0.267	0.309/ 0.267
Volume lipid (o/i) [Å^3]	1144	1146	1146	1146	1146	1146
Fraction of unilamellar vesicles	0.95	0.89	0.83	0.59	0.45	0.41
Nu	1	0.5	1.0	1.0	1.5	1.5
Number of bilayers	2.0	3.0	4.0	5.0	5.0	5.0
Sigma_d	2.0	2.0	2.0	2.0	2.0	2.0
Distance between lamella [Å]	65	65	65	64	64	64

Eta	0.1	0.05	0.05	0.1	0.05	0.05
Polydispersity (gaussian model)	0.37	0.38	0.38	0.45	0.45	0.45
Fraction of TX-100 in micelles	0.965	0.965	0.965	0.965	0.964	0.964
SLD average head [cm^{-2}]	1.365E+11	1.365E+11	1.365E+11	1.365E+11	1.364E+11	1.364E+11
SLD average tail [cm^{-2}]	8.379E+10	8.379E+10	8.379E+10	8.379E+10	8.359E+10	8.359E+10

Table S47: *Model fit parameters from analysis of the kinetic SAXS data from 2.5mg/ml DPPC vesicles mixed with 1.25mg/ml of TX-100 at 10°C at different timepoints from mixing*

**Model fits of SAXS data from 2.5mg/ml DPPC vesicles mixed with 1.25 mg/ml TX-100 at 10°C**		
	11min26s	48min
**Vesicle model**		
Average radius [Å]	670	490
Thickness of i/o tail shell [Å]	14.9	14.9
Thickness of i/o head shell [Å]	10.5/8.5	10.5/8.5
Smearing (i/o tail region) [Å]	3.0	3.2
Smearing (i/o head region) [Å]	2.0/2.0	2.0/2.0
Hydration of i/o head shell*	0.320/ 0.250	0.302/ 0.260
Volume lipid (o/i) [$Å^3$]	1145	1146
Fraction of unilamellar vesicles	0.78	0.38
Nu	0.5	1.5
Number of bilayers	2.0	5.0
Sigma_d	2.0	2.0
Distance between lamella [Å]	68	64
Eta	0.05	0.05
Polydispersity (gaussian model)	0.38	0.45
Fraction of TX-100 in micelles	0.85	0.84
SLD average head [cm^{-2}]	1.363E+11	1.361E+11
SLD average tail [cm^{-2}]	8.369E+10	8.359E+10

S4.6: DMPC:TX-100 mixture at 20°C

Table S48: *Model fit parameters from analysis of the kinetic SAXS data from 2.5mg/ml DMPC vesicles mixed with 40mg/ml of TX-100 at 20°C at different timepoints from mixing.*

**Model fits of SAXS data from 2.5mg/ml DMPC vesicles mixed with 40 mg/ml TX-100 at 20°C**							
	1s	4s	16s	45s	73s	138s	405s
**Vesicle + ellipsoidal TX-100 micelle model**							
Average radius [Å]	240	500	500	500	600	600	600
Thickness of i/o tail shell [Å]	16.1	16.1	16.1	16.1	16.1	16.1	16.1
Thickness of i/o head shell [Å]	9.7/ 7.5	9.7/ 7.5	9.7/ 7.5	9.7/ 7.5	9.7/ 8.2	9.7/ 8.2	9.7/ 8.2
Smearing (i/o tail region) [Å]	5.0	5.0	5.0	5.0	5.0	5.0	5.0
Smearing (i/o head region) [Å]	4.0/ 3.0	4.0/ 3.0	4.0/ 3.0	4.0/ 3.0	4.0/ 3.0	4.0/ 3.0	4.0/ 3.0

Hydration of i/o head shell*	0.129/ 0.172	0.214/ 0.130	0.205/ 0.120	0.151/ 0.060	0.058/ 0.024	0.089/ 0.056	0.003/ 0.004
Volume lipid (o/i) [Å^3]	1153	1156	1156	1156	1156	1156	1156
Polydispersity (gaussian model)	0.67	0.67	0.67	0.67	0.67	0.67	0.067
Fraction of lipid in micelle	0.1	0.2	0.41	0.67	0.83	0.92	0.99
Fraction of TX-100 in micelles	0.996	0.997	0.998	0.998	0.998	0.999	0.9995
Aggregation number	69	69	69	65	65	64	64
Axial ratio	0.66	0.66	0.66	0.66	0.66	0.71	0.73
Micellar radius [Å]	34.0	34.0	34.0	32.5	32.5	32.0	31.5
Density profile sigma	0.18	0.18	0.18	0.19	0.19	0.20	0.2
Smearing	0.05	0.05	0.05	0.05	0.05	0.05	0.05
Fraction of TX-100 tails in core	0.37	0.37	0.37	0.39	0.39	0.4	0.41
SLD average head [cm^{-2}]	1.366 E+11	1.370 E+11	1.367 E+11	1.348 E+11	1.318 E+11	1.326 E+11	1.247 E+11
SLD average tail [cm^{-2}]	8.288 E+10	8.251 E+10	8.260 E+10	8.255 E+10	8.246 E+10	8.249 E+10	8.212 E+10

S4.7: DPPC:TX-100 mixture at 30°C

Table S49: *Model fit parameters from analysis of the kinetic SAXS data from 2.5mg/ml DPPC vesicles mixed with 2.5mg/ml of TX-100 at 30°C at different timepoints from mixing.*

**Model fits of SAXS data from 2.5mg/ml DPPC vesicles mixed with 2.5 mg/ml TX-100 at 30°C**			
	22ms	108ms	547ms
**Vesicle + TX-100 micelle model**			
Average radius [Å]	276	334	393
Thickness of i/o tail shell [Å]	15.2	13.3	12.6
Thickness of i/o head shell [Å]	9.6/8.8	9.7/8.8	10.3/9.2
Smearing (i/o tail region) [Å]	5.0	5.0	5.0
Smearing (i/o head region) [Å]	3.2/2.5	3.2/2.5	3.2/2.5
Hydration of i/o head shell*	0.203/0.331	0.177/0.260	0.218/0.261
Volume lipid (o/i) [Å^3]	1164	1183	1189
Fraction of TX-100 in micelles	0.93	0.73	0.60
SLD average head [cm^{-2}]	1.366E+11	1.306E+11	1.288E+11
SLD average tail [cm^{-2}]	8.183E+10	8.014E+10	7.971E+10

Table S50: *Model fit parameters from analysis of the kinetic SAXS data from 2.5mg/ml DPPC vesicles mixed with 2.5mg/ml of TX-100 at 30°C at different timepoints from mixing.*

**Model fits of SAXS data from 2.5mg/ml DMPC vesicles mixed with 2.5 mg/ml TX-100 at 30°C**				
	4.5s	30.2s	140.4s	5min
**Vesicle + bicelle model**				
Average radius [Å]	333	333	333	333
Thickness of i/o tail shell [Å]	10.5	10.5	10.5	10.5
Thickness of i/o head shell [Å]	11.0/10.0	11.0/10.0	11.0/10.0	11.0/10.0
Smearing (i/o tail region) [Å]	4.0	4.0	4.0	4.0
Smearing (i/o head region) [Å]	3.2/2.5	4.0/3.0	4.0/3.0	4.0/3.0
Hydration of i/o head shell*	0.355/ 0.407	0.365/ 0.415	0.221/ 0.283	0.239/ 0.300

Volume lipid (o/i) [Å^3]	1190	1190	1190	1190
Fraction of lipid in micelle	0.33	0.67	0.86	0.95
Fraction of TX-100 in micelles	0.68	0.85	0.88	0.96
Radius bicelle [Å]	37.0	37.0	37.0	43.0
Thickness of bicelle tail region disc [Å]	21.6	21.6	21.6	21.2
Thickness of bicelle head shell [Å]	19.6	17.6	17.6	17.6
Thickness of bicelle rim [Å]	12.2	12.2	12.2	12.2
Volume lipid in bicelle [Å^3]	1161	1176	1176	1174
Hydration of head shell/sim*	0.654/ 0.851	0.654/ 0.876	0.654/ 0.876	0.675/ 0.871
Fraction of lipid/TX-100 in rim	0.06/ 0.42	0.06/ 0.42	0.06/ 0.42	0.06/ 0.42
Interface smearing	4.0	4.0	4.0	4.0
SLD average head vesicle [cm^{-2}]	1.277E+11	1.280E+11	1.243E+11	1.247E+11
SLD average tail vesicle [cm^{-2}]	7.967E+10	7.966E+10	7.982E+10	7.980E+10
SLD average head bicelle surface [cm^{-2}]	1.232E+11	1.257E+11	1.269E+11	1.269E+11
SLD average head bicelle rim [cm^{-2}]	1.165E+11	1.170E+11	1.173E+11	1.173E+11
SLD average tail bicelle [cm^{-2}]	8.153E+10	8.075E+10	8.075E+10	8.088E+10

S4.8: DMPC:TX-100 mixture at 10°C

Table S51: *Model fit parameters from analysis of the kinetic SAXS data from 2.5mg/ml DMPC vesicles mixed with 2.5mg/ml of TX-100 at 10°C at different timepoints from mixing*

**Model fits of SAXS data from 2.5mg/ml DMPC vesicles mixed with 2.5 mg/ml TX-100 at 10°C**		
	20ms	137ms
**Vesicle + TX-100 micelle model**		
Average radius [Å]	242	274
Thickness of i/o tail shell [Å]	11.6	11.6
Thickness of i/o head shell [Å]	10.3/9.2	10.3/9.2
Smearing (i/o tail region) [Å]	4.0	4.0
Smearing (i/o head region) [Å]	3.2/2.5	3.2/2.5
Hydration of i/o head shell*	0.277/0.370	0.277/0.370
Volume lipid (o/i) [Å^3]	1071	1069
Fraction of unilamellar vesicles	0.9	1
Nu	0	NA
Number of bilayers	2.0	NA
Sigma_d	0.1	NA
Distance between lamella [Å]	64	NA
Fraction of TX-100 in micelles	0.79	0.79
SLD average head [cm^{-2}]	1.326E+11	1.326E+11
SLD average tail [cm^{-2}]	8.012E+10	8.032E+10

Table S52: *Model fit parameters from analysis of the kinetic SAXS data from 2.5mg/ml DMPC vesicles mixed with 2.5mg/ml of TX-100 at 10°C at different timepoints from mixing*

**Model fits of SAXS data from 2.5mg/ml DMPC vesicles mixed with 2.5 mg/ml TX-100 at 10°C**					
	286ms	479ms	1s	80s	3min
**Vesicle + bicelle model**					
Average radius [Å]	224	224	224	224	224
Thickness of i/o tail shell [Å]	11.6	11.6	11.6	11.1	11.6
Thickness of i/o head shell [Å]	10.3/9.2	10.3/9.2	10.3/9.2	10.3/9.2	10.3/9.2
Smearing (i/o tail region) [Å]	4.0	4.0	4.0	4.0	4.0
Smearing (i/o head region) [Å]	3.2/2.5	3.2/2.5	3.2/2.5	3.2/2.5	3.2/2.5
Hydration of i/o head shell*	0.355/ 0.407	0.355/ 0.407	0.361/ 0.453	0.008/ 0.096	0.008/ 0.096
Volume lipid (o/i) [$Å^3$]	1071	1071	1071	1071	1071
Polydispersity (gaussian model)	0.43	0.43	0.43	0.43	0.43
Fraction of lipid in bicelles	0.61	0.79	0.87	0.99	0.9999
Fraction of TX-100 in bicelles	0.82	0.94	0.99	0.99	0.9999
Radius bicelle [Å]	31.8	34.8	35.2	35.2	35.2
Thickness of bicelle tail region disc [Å]	23.0	23.7	23.7	23.7	23.7
Thickness of bicelle head shell [Å]	13.6	13.6	13.6	13.6	13.6
Thickness of bicelle rim [Å]	20.0	20.0	20.0	20.0	20.0
Volume lipid in bicelle [$Å^3$]	1044	1044	1044	1044	1044
Hydration of head shell/sim*	0.607/ 0.881	0.596/ 0.873	0.596/ 0.874	0.596/ 0.874	0.596/ 0.884
Fraction of lipid/TX-100 in rim	0/0.71	0/0.71	0/0.71	0/0.71	0/0.71
Interface smearing	1.1	1.1	1.1	1.1	1.1
SLD average head vesicle [cm^{-2}]	1.281E+11	1.309E+11	1.365E+11	1.235E+11	1.162E+11
SLD average tail vesicle [cm^{-2}]	8.02711E+10	8.017E+10	8.003E+10	8.050E+10	8.127E+10
SLD average head bicelle surface [cm^{-2}]	1.303E+11	1.308E+11	1.310E+11	1.318E+11	1.317E+11
SLD average head bicelle rim [cm^{-2}]	1.149E+11	1.149E+11	1.149E+11	1.149E+11	1.149E+11
SLD average tail bicelle [cm^{-2}]	8.244E+10	8.247E+10	8.248E+10	8.252E+10	8.252E+10

S5: Cryo-TEM images

The Cryo-TEM images were collected by Dr. Marie-Sousai Appavou at the TEM instrument at Jülich Centre for Neutron Science at MLZ. The samples were vitrified from 37°C. The Gatan G910 3 positions specimen holder at 179,99°C and S166-3 lacey carbon coated grid was used. The grid was held with tweezer in Grid plunger chamber thermalized at 37°C and 85% relative humidity for some

seconds, 5 microliter was dropped onto a freshly glow discharged lacey carbon coated grid, blotted by Leica EMGP during 1 second with filter paper and plunged into liquified ethane at -180 °C.

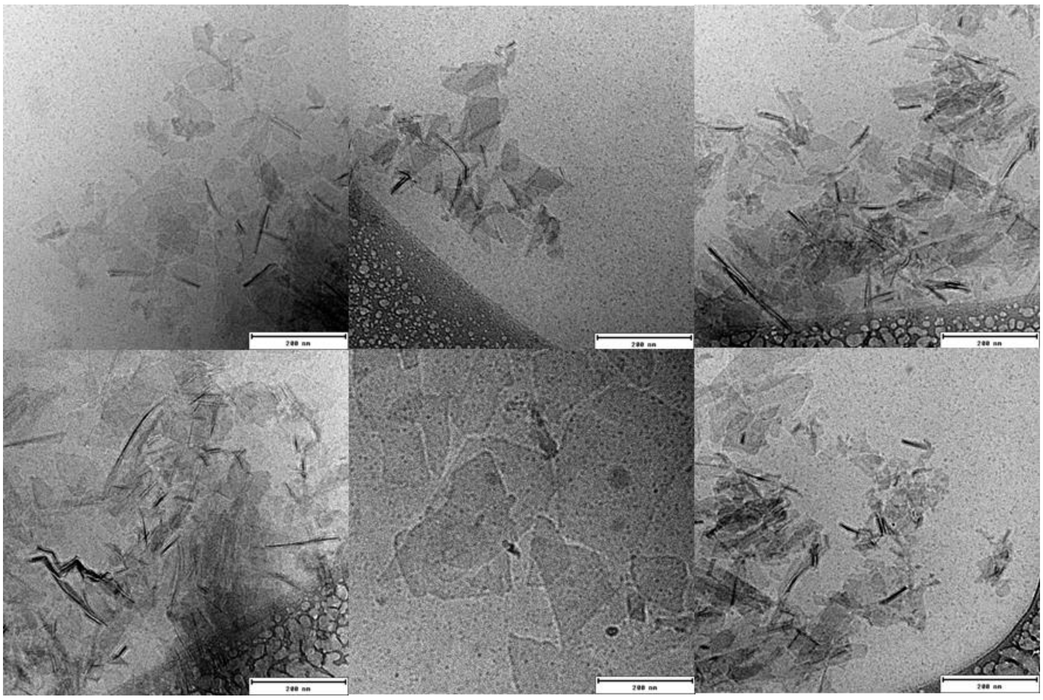

Figure S15: *Cryo-TEM images of DPPC vesicles at 2.5mg/ml mixed with 5 mg/ml TX-100 at 37°C.*

S6: References

(1) Pedersen, J. S.; Laso, M.; Schurtenberger, P. Monte Carlo study of excluded volume effects in wormlike micelles and semiflexible polymers. *Physical Review E* **1996**, *54* (6), R5917-R5920. DOI: 10.1103/PhysRevE.54.R5917. Pedersen, J. S.; Schurtenberger, P. Scattering Functions of Semiflexible Polymers with and without Excluded Volume Effects. *Macromolecules* **1996**, *29* (23), 7602-7612. DOI: 10.1021/ma9607630.

(2) Pedersen, J. S. Analysis of small-angle scattering data from colloids and polymer solutions: modeling and least-squares fitting. *Advances in Colloid and Interface Science* **1997**, *70*, 171-210. DOI: https://doi.org/10.1016/S0001-8686(97)00312-6.

(3) Lund, R.; Willner, L.; Richter, D. Kinetics of Block Copolymer Micelles Studied by Small-Angle Scattering Methods. In *Controlled Polymerization and Polymeric Structures: Flow Microreactor Polymerization, Micelles Kinetics, Polypeptide Ordering, Light Emitting Nanostructures*, Abe, A., Lee, K.-S., Leibler, L., Kobayashi, S. Eds.; Springer International Publishing, 2013; pp 51-158.
